# ATP2C2 Has Potential to Define Tumor Microenvironment in Breast Cancer

**DOI:** 10.3389/fimmu.2021.657950

**Published:** 2021-04-14

**Authors:** Jiazhou Liu, Yuxian Wei, Yushen Wu, Jie Li, Jiazheng Sun, Guosheng Ren, Hongzhong Li

**Affiliations:** ^1^Department of Endocrine and Breast Surgery, The First Affiliated Hospital of Chongqing Medical University, Chongqing, China; ^2^Key Laboratory of Molecular Oncology and Epigenetics, The First Affiliated Hospital of Chongqing Medical University, Chongqing, China

**Keywords:** breast cancer, ATP2C2, tumor microenvironment, tumor-infiltrating immune cells, LASSO, nomogram ATP2C2 modulates TME in BRCA

## Abstract

Tumor microenvironment (TME) is vital for the occurrence and development of breast cancer (BRCA). However, it remains challenging to understand the dynamic modulation of the stromal and immune components comprehensively in TME. Herein, we used ESTIMATE and CIBERSORT algorithm to estimate the number of stromal and immune components and the abundance of tumor-infiltrating immune cells (TICs) in 582 BRCA cases from gene expression omnibus (GEO) database. We employed three regression models including univariable Cox proportion, LASSO regression model and multivariate Cox regression, and identified 7 immune-specific genes related to BRCA survival. Of 7 genes, ATPase Secretory Pathway Ca^2+^ Transporting 2 (ATP2C2) attracts our attention for significantly predicting prognosis of BRCA patients. Further analysis indicated that ATP2C2 expression was closely related to the clinicopathological features (age, T- and N-staging) and negatively correlated with patients’ survival in BRCA. Gene Set Enrichment Analysis (GSEA) was performed to reveal pathway enrichment between ATP2C2^high^ and ATP2C2^low^ groups. The low ATP2C2 expression groups’ genes were mainly enriched for immune-related activities, while those in the ATP2C2 high-expression group were largely enriched in metabolic-related pathways. Notably, Pearson’s correlation analysis identified that ATP2C2 expression was positively correlated with T follicular helper (Tfh) cells, and negatively correlated with gamma delta (γδ) T cell, suggesting that ATP2C2 might be accountable for the maintenance of immune-dominant status for TME. To sum up, this study comprehensively analyzed the TME and shed light on prognostic immune-related biomarkers for BRCA. In particular, ATP2C2 might be helpful for predicting the prognosis of BRCA patients, which provided an extra insight for BRCA treatment.

## Introduction

Breast cancer (BRCA) with high aggressiveness is one of the most common malignant tumors that seriously endanger women’s health (1). However, the mechanism of its occurrence and development is not yet fully understood. Therefore, in-depth study of the molecular mechanisms of BRCA development and finding effective therapeutic targets are of great significance for improving the prognosis of BRCA patients.

The “seed-soil” theory of tumors believes that the growth of tumor cells requires the surrounding normal cells and extracellular matrix to provide a permissive environment, that is, the tumor microenvironment (TME) (2). TME is the local environment for tumor cells to survive. In addition to tumor cells, it also contains resident stromal cells and recruited immune cells. These components are crucial in the occurrence, development, and immune evasion of tumors (3). Although stromal cells can promote tumor angiogenesis, cancer cell proliferation, invasion and metastasis, the detailed molecular mechanism to promote cancer progression has not been fully elucidated. Meanwhile, increasing attention has been paid to the influence of the immune cells in TME on tumor development. It was revealed that high levels of immune cell infiltration into cancer tissues were correlated with favorable outcomes, suggesting that valuing TME heterogeneity and remodeling the immune microenvironment may hold promise for cancer treatment (4). Immune cells in the TME can affect the host immune response by secreting chemokines, cytokines and other factors that directly or indirectly suppress or support tumor progression (5). Therefore, to better understand the immune status of the TME and investigating the distribution pattern and function of immune cells are essential for improving the effectiveness of cancer immunotherapy.

In the present study, we used ESTIMATE and CIBERSORT algorithm to quantify the tumor-infiltrating immune cell (TIC) proportion and the ratio of immune and stromal components of BRCA samples from the gene expression omnibus (GEO) database and identified a novel predictive biomarker, ATPase Secretory Pathway Ca^2+^ Transporting 2 (ATP2C2). Gene dysregulation of ATP2C2 has been linked to breast cancers: ATP2C2 (also known as SPCA2) promotes tumor growth by increasing Ca^2+^ entry through activation of the Orai1 calcium channel (6). Recently, the literature has demonstrated a novel association among ATP2C2, Kv10.1 and Orai1 involved in mediating transduction signals from TME to the BRCA cells, suggesting that ATP2C2 may play an important role in TME (7). Here we set out to compare differentially expressed genes (DEGs) produced by comparison between stromal components and immune components in BRCA cases, revealing that the ATP2C2 may be a potential indicator for the alteration of TME status in BRCA.

## Materials and Methods

### BRCA Datasets and Samples

mRNA expression data for breast cancer (BRCA) were procured from GEO (https://www.ncbi.nlm.nih.gov/geo/). The keywords “Breast cancer” and “Survival” were used for retrieval. Finally, five gene expression microarray datasets were chosen as a training set, including (GSE42568, n=121), (GSE88770, n=117), (GSE16446, n=120), (GSE37751, n=108), and (GSE7390, n=198). 582 samples with the survival status and survival time were downloaded for DEG analysis from the five datasets. In addition, the GSE20711 cohort (n = 88) were used as a validation set.

Harmonized RNA sequencing data and related clinical information for BRCA were downloaded for clinical correlation and GSEA analysis from TCGA (https://portal.gdc.cancer.gov/, up to July 30, 2020), which included 1222 samples, 113 normal tissue samples, and 1109 tumor samples.

### ESTIMATE

The stromal, immune and ESTIMATE scores were outputted by the R package “estimate” (8).

### DEGs Identification Based on StromalScore and ImmuneScore

**Figure 1:**
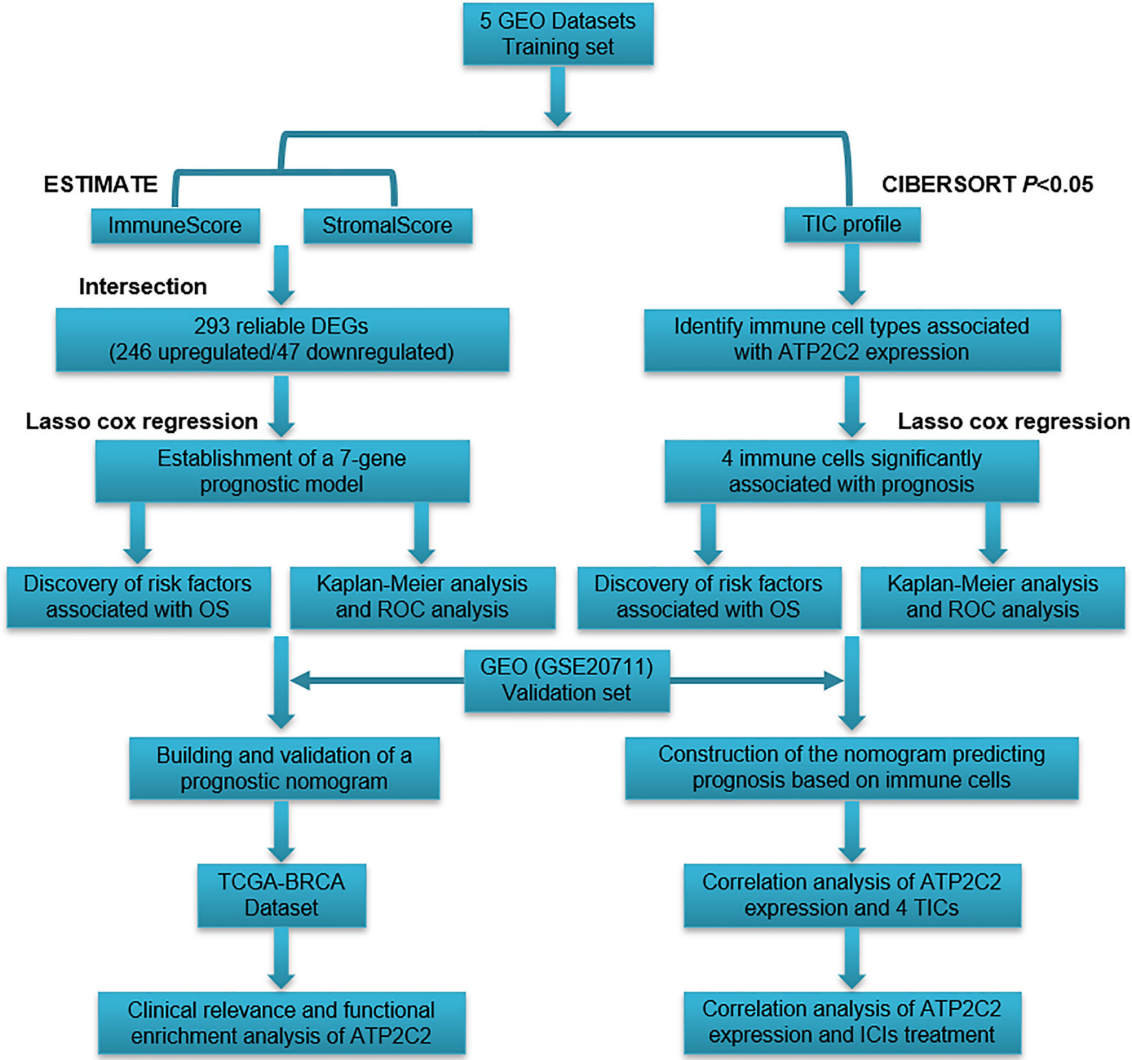
Experimental technical roadmap.

Based on the comparison with the median scores of StromalScore and ImmuneScore, 582 BRCA cases were divided into high or low score groups. Limma R package was applied to determine DEGs between high-scoring samples and low-scoring samples. Genes with *p* < 0.05 and |log2 FC (fold-change)| > 1 were screened DEGs. A flowchart for constructing DEG signature is displayed in **Figure 1**.

### Heatmaps

Heatmaps were plotted with the R package pheatmap (9).

### Functional Enrichment and Pathway Analysis

To understand the potential function of the common DEGs that stemmed from the immune scores and stromal scores, Gene Ontology (GO) and the Kyoto Encyclopedia of Genes and Genomes (KEGG) pathway enrichment analysis were performed by “clusterProfiler” package in R. GO and KEGG terms with a *p*- and *q*-value of both <0.05 were considered significantly enriched.

### Construction and Validation of the Prognostic Prediction Models

A univariate Cox model was conducted to screen potential prognostic immune-related genes (IRGs) and TICs. A LASSO Cox regression model was further applied to narrow the range of prognostic IRGs and TICs. Next, a multivariate Cox regression model was utilized to select IRGs and TICs most closely related to survival, and those IRGs and TICs were used to construct two risk models. Two formulas for the IRGs risk score and TICs risk score was established to predict patient survival: IRGs risk score = (−0.212 × expression level of ADRB2) + (0.208 × expression level of ATP2C2) + (−0.287 × expression level of CELF2) + (−0.129 × expression level of CXCL12) + (0.257 × expression level of LGMN) + (−0.204 × LIPA) + (0.214 × expression level of SLCO2B1), TICs risk score = (3.359 × abundance of naïve B cells) + (4.957 × abundance of T follicular helper (Tfh) cells) + (−4.349 × abundance of gamma delta (γδ) T cells) + (−3.966 × abundance of resting mast cells). The individual IRGs risk score and TICs risk score in both training set and validation set were calculated according to the formulas accordingly.

The Kaplan-Meier method and the ROC curves were used to analyze OS and to assess signature’s sensitivity and specificity of the models.

### Construction and Validation of Nomograms

Based on seven IRGs and four TICs, we separately plotted the nomograms to predict the probability of 1-, 2-, and 3-OS of BRCA patients. Validation of the nomograms was evaluated by the discrimination and calibration.

### Gene Set Enrichment Analysis (GSEA)

GSEA was performed between high- and low-ATP2C2 expression (10). The number of random sample permutations was set at 1000, and NOM *p*-value < 0.05, FDR *q*-value < 0.25, and | NES | > 1 were set as the significance threshold.

### Estimation of TIC Types

CIBERSORT algorithm was utilized to evaluate the relative abundance of 22 types of TICs. After the quality filtering (*p*-value < 0.05), 545 BRCA samples were selected for following analysis.

### Tumor Immune Dysfunction and Exclusion (TIDE) Analysis

TIDE is a computational framework construct to predict immune checkpoint inhibitors (ICIs) response (11).

## Results

### DEGs Based on ImmuneScore and StromalScore Were Mainly Presented as the Enrichment of IRGs

**Figure 2:**
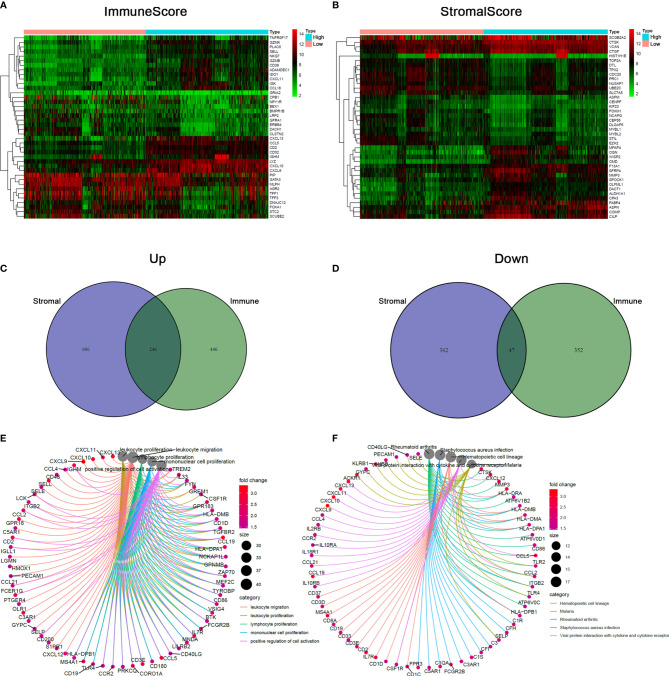
Comparison of gene expression profile with stromal and immune scores of BRCA. **(A, B)** The heatmap presenting the top 20 upregulated and downregulated DEGs in ImmuneScore and StromalScore. DEGs were detected by Wilcoxon rank sum test (*q* < 0.05 & |log2FC| > 0.5). **(C, D)** Venn diagram analysis of aberrantly expressed genes based on stromal and immune scores. **(E, F)** DEGs-related biological functions and pathways in BRCA, terms with *p* and *q* < 0.05 were considered to be significantly enriched.

To explore the accurate alterations of gene profile in TME regarding immune and stromal components, a comparative analysis of high- and low-scoring samples was performed. A total 1091 DEGs including 692 upregulated and 399 downregulated genes were obtained from ImmuneScore (**Figure 2A**). Similarly, a total of 1261 DEGs including 852 upregulated and 409 downregulated genes were obtained from StromalScore (**Figure 2B**). By intersecting these DEGs from ImmuneScore and StromalScore, a total of 246 up-regulated genes and 47 down-regulated genes (**Figures 2C, D**). These DEGs were probably key factors for the status of TME. GO enrichment analysis showed that the functions of the DEGs were predominantly associated with the immune response, such as leukocyte proliferation and leukocyte migration (**Figure 2E**; **Figure S1A**). The KEGG enrichment analysis also indicated that the DEGs were involved in hematopoietic cell lineage, Th1 and Th2 cell differentiation, and cytokine–cytokine receptor interaction (**Figure 2F**; **Figure S1B**). Therefore, immune-related activities tended to represent the main functions of DEGs, implying that the involvement of immune components was a predominant feature of TME of BRCA.

### Screening of Prognosis-Specific IRGs and Construction of Prognosis Prediction Model

**Figure 3:**
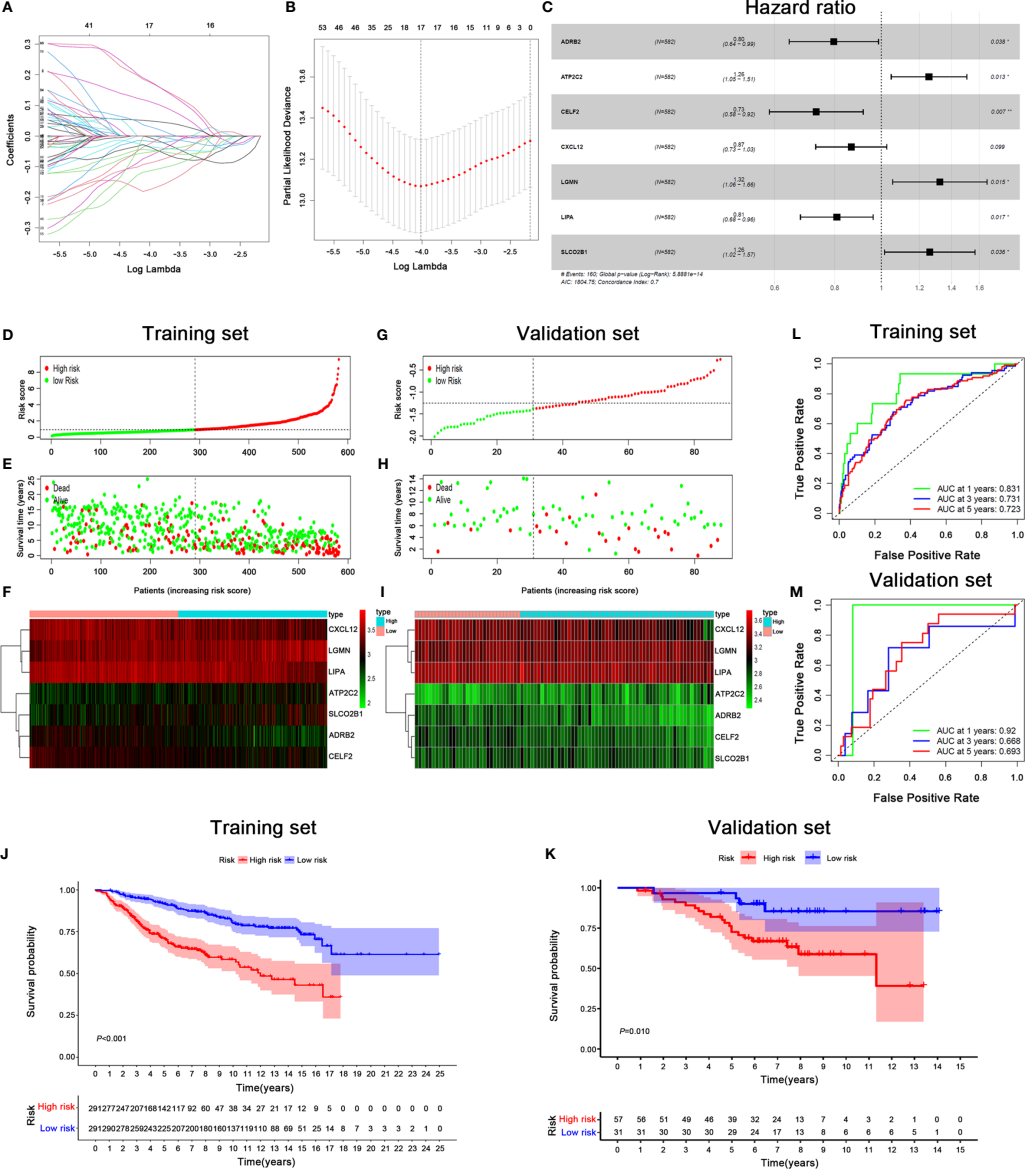
Signature-based risk score is a promising marker of survival in BRCA patients. **(A, B)** LASSO Cox analysis identified seven genes most correlated with OS, and 10-round cross validation was performed to prevent overfitting. **(C)** Multivariate Cox analysis of 7 immune-related hub genes. **(D, G)** Risk score distribution in training set **(D)** and validation set **(G)**. **(E, H)** Survival overview in training set **(E)** and validation set **(H)**. **(F, I)** Expression profile of 7 immune genes in training set **(F)** and validation set **(I)**. **(J, K)** Kaplan–Meier estimates of OS according to the seven-immune-related gene signature in training set **(J)** and validation set **(K)**. The differences between the two curves were evaluated by the two-side log-rank test. **(L, M)** The ROC curve analysis of the seven-immune-related gene signature for predicting OS in training set **(L)** and validation set **(M)**.

293 differentially expressed IRGs were further subjected to the univariate Cox regression model; we identified 67 IRGs significantly related with OS (**Table S1**). Then, these 67 IRGs were used in the LASSO regression for feature selection. A set of seventeen genes (ADD3, ADRB2, ALDH1A1, ATP2C2, CCR2, CELF2, CRTAM, CXCL12, EPN3, HOXC8, IL2RB, ITM2A, KLRG1, LGMN, LIPA, SLCO2B1, and TMEM243) and their coefficients were computed (**Figures 3A, B**). Then, the Akaike information criterion (AIC) was utilized to screen significant prognostic IRGs in multivariate Cox regression models (**Figure 3C**).

The risk score of each BRCA patient was calculated, and the patients were classified into the high-risk (n = 291) or low-risk (n = 291) group by the median cut-off value (**Figure 3D**). Remarkably, the number of deaths was significantly higher in the high-risk group (**Figure 3E**). A heatmap revealed that patients in the high-risk group tended to have increased LGMN, ATP2C2, and SLCO2B1 expression levels, as well as decreased expression levels of CXCL12, LIPA, ADRB2 and CELF2 (**Figure 3F**). The Kaplan–Meier analysis indicated that low-risk patients had a better OS than high-risk patients (*P* < 0.001) (**Figure 3J**). The accuracy of the prognostic model was displayed in the ROC curve. The AUCs under ROC curve for predicting OS in the first, third, and fifth year were 0.831, 0.731, and 0.723, respectively (**Figure 3L**). To demonstrate the robustness of the prognostic signature, the predictive ability was assessed in a GSE20711 cohort. The risk score for each patient was calculated based on the same formula, and the best threshold was chosen as the cut-off for patients stratified as high- and low-risk. The risk score profiles and gene expression are displayed in **Figures 3G–I**. Patients in high-risk group had a shorter OS than patients in the low-risk group in the GSE20711 cohort, which was consistent with the results of the training set (**Figure 3K**). The AUC of the signature for OS was 0.92, 0.668 and 0.693 at 1, 3 and 5 years, respectively (**Figure 3M**).

**Figure 4:**
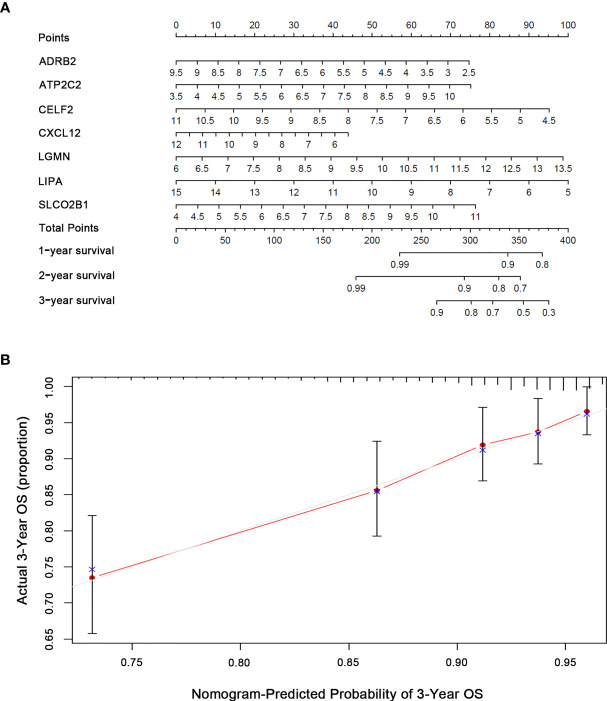
Nomogram predicting OS for BRCA patients based on risk score. **(A)** Nomogram model for predicting the probability of 1-, 2-, and 3-year OS of BRCA. Points are assigned for seven features. The sum of these points is located on the total points axis. The total points on the bottom scales correspond to the predicted 1-, 2-, and 3-year survival. **(B)** Calibration plots of the nomogram for predicting the probability of OS at 1, 2, and 3 years. The X-axis represents nomogram-predicted survival, and the Y-axis represents actual survival.

Based on the multivariate Cox analysis results, seven IRGs were integrated in the nomogram to predict the OS of BRCA patients (**Figures 4A, B**). The calibration curves revealed acceptable accuracy. These results indicated that the IRGs risk model nomogram had very appropriate calibration.

### ATP2C2 Is Highly Expressed in BRCA and Correlates With Unfavorable Prognosis

**Figure 5:**
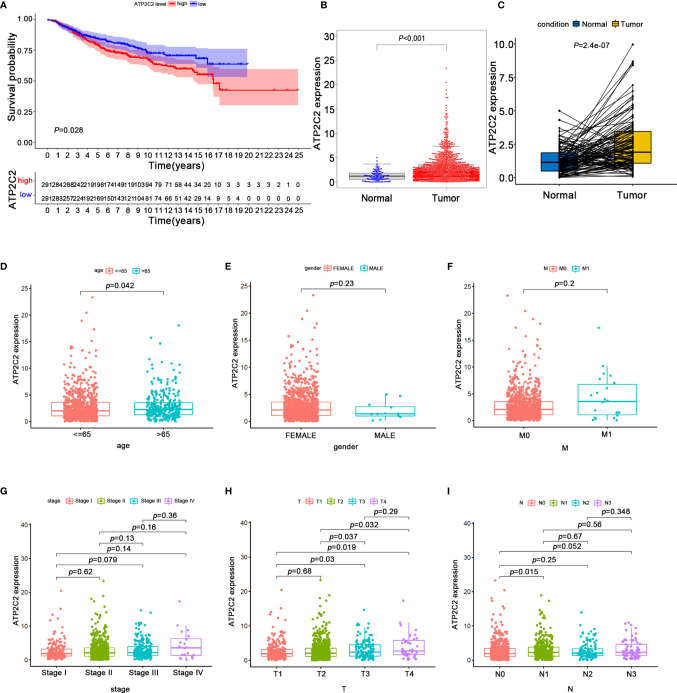
The expression of ATP2C2 and correlation with OS and clinicopathological features of BRCA patients revealed by bioinformatic analysis. **(A)** Survival outcome for BRCA patients with different ATP2C2 expression. The differences between the two curves were detected by the two-side log-rank test. **(B)** ATP2C2 mRNA is highly expressed in BRCA tissues. Statistical significance was evaluated using Wilcoxon-Mann-Whitney test. **(C)** ATP2C2 mRNA levels in the paired BRCA tissues were evaluated. *P*-values are based on the Wilcoxon Test. **(D–I)** The correlation of ATP2C2 expression with clinicopathological features. Statistical significance was evaluated using Wilcoxon rank sum test.

Ion channels have a key role in mediating TME signal (12). Previous studies had shown that ATP2C2 can regulate the localization and the activity of Kv10.1 and Orai1 channels, mediating transduction signals from TME to the BRCA cells (7). However, the clinical role of the ATP2C2 has not yet been determined. Here we divided all BRCA samples into ATP2C2 high and low expression groups based on ATP2C2 median expression. The Kaplan–Meier analysis elucidated that BRCA patients with ATP2C2 low expression had longer survival than those with ATP2C2 high expression (**Figure 5A**). In addition, we further found that ATP2C2 levels are associated with poor prognosis of various cancers, including thyroid carcinoma (THCA), head-neck squamous cell carcinoma (HNSC), kidney renal clear cell carcinoma (KIRC), lung squamous cell carcinoma (LUSC), and esophageal squamous cell carcinoma (ESCC) on a web platform Kaplan–Meier Plotter (http://kmplot.com/analysis/) (**Figures S2A–E**). After that, clinical pathologic features and ATP2C2 expression were downloaded from the TCGA database. The expression of ATP2C2 in the BRCA samples was significantly higher than that in normal breast tissues or paired breast non-tumor by Wilcoxon rank sum test (**Figures 5B, C**). We also observed a similar up-regulation of ATP2C2 expression in BRCA in the HPA dataset (https://www.proteinatlas.org/) (**Figures S3A–F**). Collectively, these outcomes clearly demonstrated that high ATP2C2 expression in TME was correlated with worse prognosis of BRCA patients. Remarkably, the levels of ATP2C2 were increased along with the progression of age, T- and N- staging (**Figures 5D–I**).

### ATP2C2 Had Potential to Be an Indicator for TME Modulation

**Figure 6:**
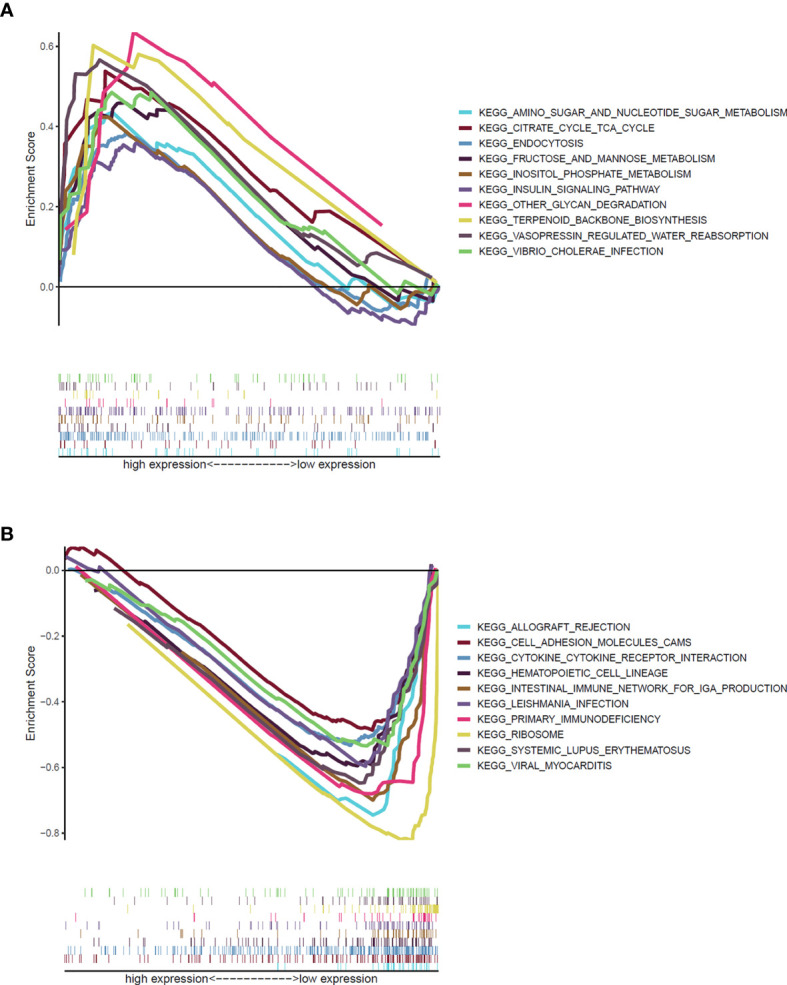
GSEA revealed biological pathways correlated with ATP2C2 in the cohort from TCGA. **(A)** Gene sets enriched in ATP2C2 high-expression group. **(B)** Gene sets enriched in ATP2C2 low-expression group. NOM *p* < 0.05, FDR *q* < 0.25, and |NES| > 1 are set as the significance threshold.

Given the expressions of ATP2C2 were negatively associated with the OS, T- and N- staging of patients with BRCA, GSEA was performed to explore the gene sets enriched in different ATP2C2 subgroups. As shown in **Figure 6A**, ATP2C2 low-expression group’ genes were significantly enriched in immune-related activities, such as cytokine–cytokine receptor interaction, hematopoietic cell lineage and primary immunodeficiency, while those in ATP2C2 high-expression group were mainly enriched in metabolic pathways, including citrate cycle TCA cycle, amino sugar and nucleotide sugar metabolism, and fructose and mannose metabolism (**Figure 6B**). Together, these findings implied that ATP2C2 might be a potential indicator for the status of TME.

### ATP2C2 Predicts the Infiltration of Immune Cells Into BRCA Microenvironment

**Figure 7:**
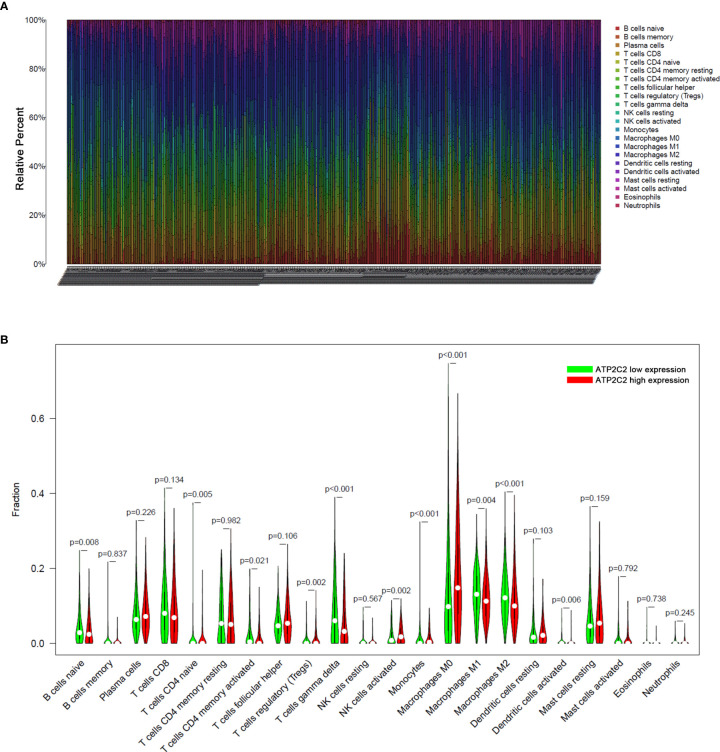
Analysis of immune cell infiltration. **(A)** Fraction data of the 22 types of immune cells. **(B)** Differential immune cell type expression was observed between the high and low-ATP2C2 groups. Significant statistical differences between the two subgroups were assessed using the Wilcoxon test.

To further explore the indicative roles of ATP2C2 on TME, we used the CIBERSORT algorithm to detect the proportions of 22 kinds of immune cells in the BRCA microenvironment (**Figure 7A**). Besides, the results of the Wilcoxon-Mann-Whitney test showed that the fractions of the naïve B cells, γδ T cells, activated memory CD4^+^ T cells, naïve CD4^+^ T cells, monocytes, M0 macrophages, M2 macrophages, and activated dendritic cells in ATP2C2 high-expression group was relatively less than that in ATP2C2 low-expression group, and regulatory T cells (Tregs), activated NK cells, and M1 macrophages were relatively greater in ATP2C2 high-expression group (**Figure 7B**).

### ATP2C2 Was Correlated With Distribution Pattern of T Cell Subsets

All TICs were integrated into a univariate Cox regression model (**Table 1**). The significative TICs, screened by univariate Cox regression analysis were subjected to LASSO Cox regression analysis. The results of the Lasso regression suggested that the model was not overfitting (**Figure S4A, B**). After that, multivariate Cox regression analysis revealed that the model composed of naïve B cells, Tfh cells, γδ T cells, and resting mast cells had the smallest AIC (**Figure S4C**). According to the median cut-off value of TICs risk score, BRCA patients were classified into high- and low-risk groups. The distribution of risk score, survival status and expression profile of the four TICs of each patient are presented in **Figures S4D–F** (training set) and **Figures S4G–I** (validation set). The Kaplan–Meier survival analysis in the two datasets revealed significantly worse prognosis in the high-risk group (**Figures S4J, K**). Next, the prognostic accuracy of the TICs risk score was examined in the training set and validation set by using time‐dependent ROC curves analysis (**Figures S4L, M**).

**Table 1:** Univariate analysis showing associations between different immune cell subsets and OS in BRCA. Unadjusted HRs are shown with 95 percent confidence intervals.

Variables	Univariate analysis			
	HR	HR.95L	HR.95H	*P*-value
Naïve B cells	51.91709028	1.393224791	1934.637024	0.032386271
T follicular helper cells	60.68385192	1.593168431	2311.450448	0.027054021
Gamma delta T cells	0.018187256	0.001155435	0.286278542	0.004380174
M0 macrophages,	5.614738081	1.866935106	16.88611651	0.002131852
Resting dendritic cells	0.003802593	1.49E-05	0.970254058	0.048764644
Resting mast cells	0.013876877	0.000785406	0.245182503	0.00350731

HR, hazard ratio.

To further improve the accuracy of the prognostic, the nomogram based on the multivariate analysis was constructed (**Figure S5A**). In addition, the calibration curve and the ROC demonstrated good discrimination and concordance (**Figure S5B**).

**Figure 8:**
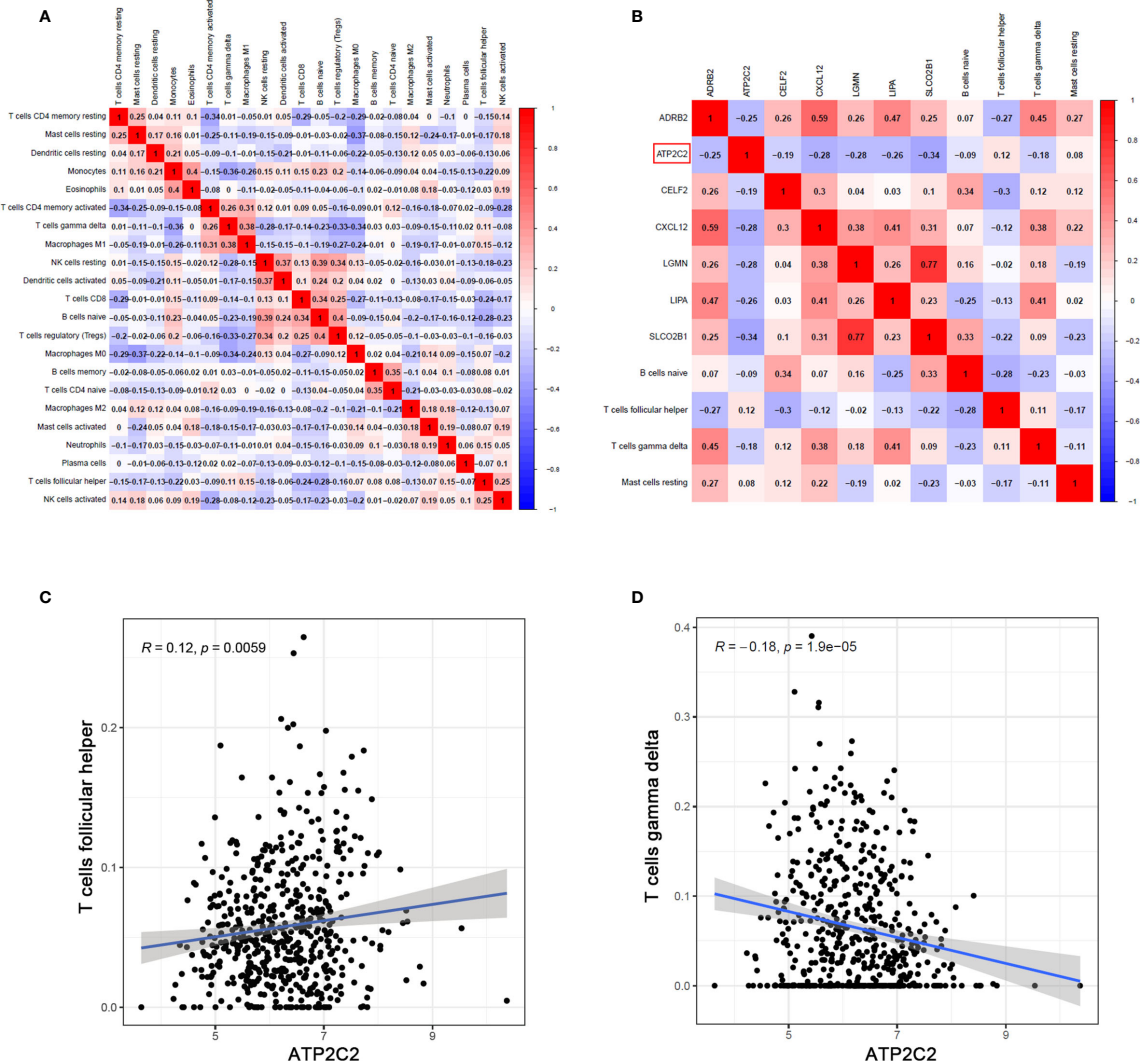
Correlation of TICs proportion with ATP2C2 expression. **(A, B)** Correlation analysis of different TICs and the relationships between different TICs and DEGs in tumor tissues of BRCA. Pearson’s correlation coefficient (*r*) was used for the significance test. **(C, D)** Correlation between Tfh cells, γδ T cells and ATP2C2 expression. Correlation test is conducted by the Pearson coefficient. *p*-value < 0.01 is the significance threshold.

Pearson analysis was applied to demonstrate the co-expression patterns among diversified immune cells (**Figure 8A**). Likewise, correlation relationship between immune cells and key genes was further analyzed and illustrated (**Figure 8B**). Here we emphatically analyzed the correlation between T cells and ATP2C2. Among them, Tfh cells were positively correlated with ATP2C2 expression (*R* = 0.12, *P* = 0.0059) (**Figure 8C**); γδ T cells was negatively correlated with ATP2C2 expression (*R* = -0.18, *P* = 1.9e-05) (**Figure 8D**). These outcomes further verified that the expressions of ATP2C2 influenced the immune activity of TME.

### ATP2C2 Promotes Immune Evasion and Resistance to ICIs

**Figure 9:**
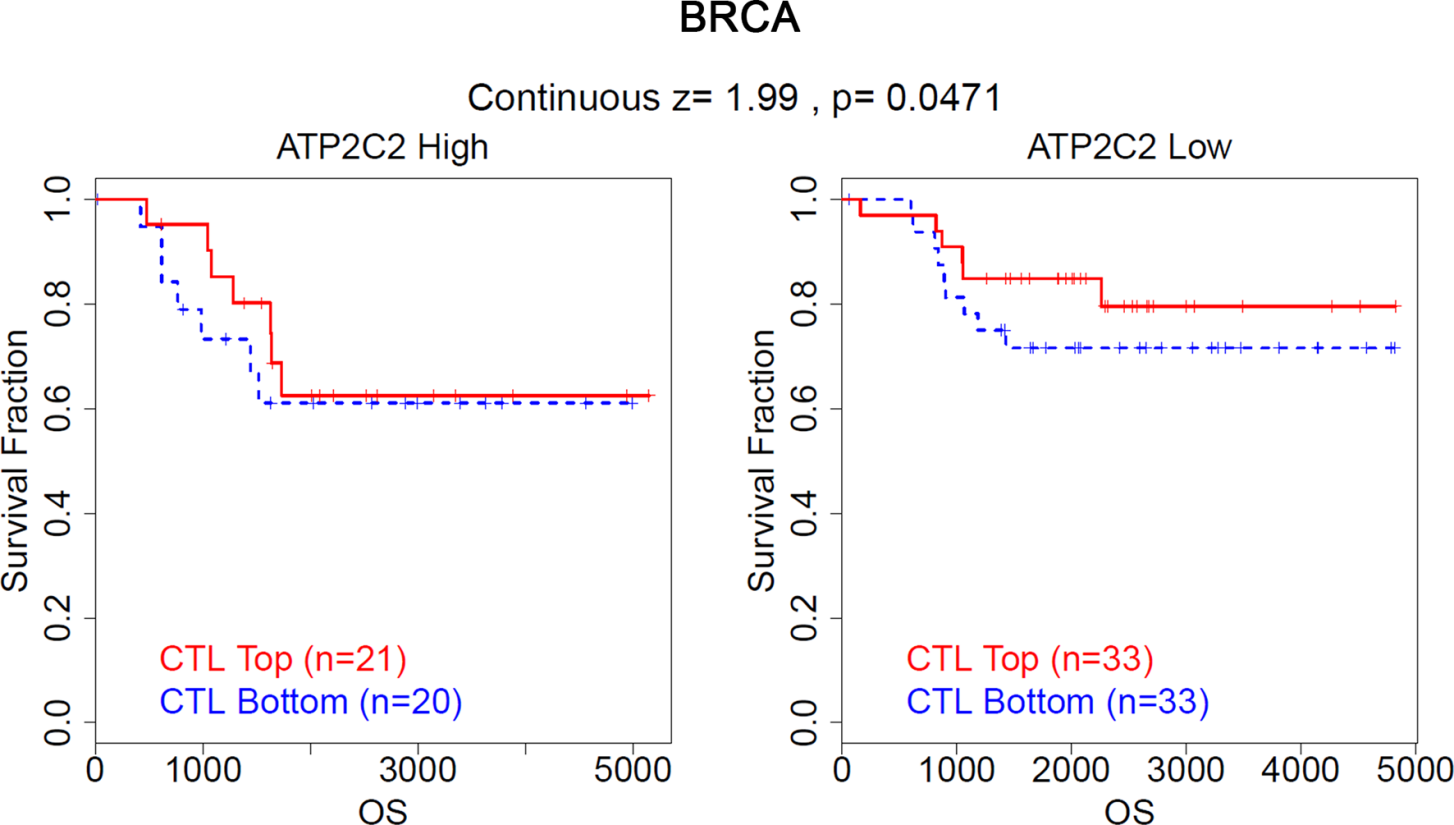
Validation of ATP2C2 as a regulator of tumor immune escape. The association between the CTL level and OS for BRCA with different ATP2C2 levels.

Accumulating evidence suggests that the level of cytotoxic T lymphocytes (CTL) is correlated with a better prognosis of patients. Through the analysis of the TIDE algorithm, we found that in BRCA patients with low ATP2C2 expression levels, high CTL levels indicate a better prognosis, while the above phenomenon is not observed in BRCA patients with high ATP2C2 expression levels (http://tide.dfci.harvard.edu/) (**Figure 9**). Similar results are observed in acute myeloid leukemia (AML), lung adenocarcinoma (LUAD), and ovarian serous cystadenocarcinoma (OV) patients (**Figures S6A–C**). In addition, we further used the TIDE algorithm to predict the efficacy of patients with ICIs. We found that the expression level of ATP2C2 is related to the enhanced efficacy of ICIs treatment in melanoma and KIRC (**Figures S6D, E**). These results suggested that ATP2C2 may be appropriate candidates for immunotherapy, especially ICIs.

## Discussion

Herein, we sought to mine TME-related genes that contributed to the classification of TNM stages and the OS in BRCA patients from the GEO and TCGA-BRCA datasets. ATP2C2 was determined to be involved in immune activities. Interestingly, we found that ATP2C2 might be an indicator for the status of TME in BRCA patients through bioinformatics analysis.

TME is the key regulator of carcinogenesis and consists of tumor cells, stromal cells, and immune cells (13, 14). TICs are closely associated with tumorigenesis, angiogenesis and the growth and metastatic potential of tumor cells, which could alternately modulate the pattern of immune cells (15–18). Now, immunotherapy has been widely used to treat a broad spectrum of cancers including BRCA (19–22). However, not all patients can benefit from it. Thus, it is of crucial significance to improve treatment efficacy of BRCA and to uncover strong prognostic biomarkers for BRCA. Here, 7 prognosis-specific IRGs were identified by a series of bioinformatics analysis. The risk score calculated using the 7 IRGs was an independent prognostic factor for BRCA. Among all 7 prognosis-specific IRGs, four (e.g., ADRB2, CXCL12, LGMN, LIPA) have been reported to be involved in the immune microenvironment-associated pathogenesis of BRCA, implying that our bioinformatics analysis using GEO cohorts has prognostic value. The remaining three genes including ATP2C2, CELF2, and SLCO2B1 have not been formerly reported to be associated with BRCA patients’ prognosis and could serve as novel potential biomarkers for BRCA. Here we gave special attention to ATP2C2 and then embarked from the transcriptomic analysis of BRCA in TCGA database, which revealed that increased ATP2C2 expression was significantly correlated with the advanced clinicopathological characteristics (age, T‐ and N‐staging) and unfavorable prognosis of BRCA patients.

ATP2C2 is mainly expressed in salivary glands, gastrointestinal and respiratory tracts, and mammary gland, and participates in Ca^2+^ transport in secretion (23). ATP2C2 has been shown to be involved in many important biological functions, such as the regulation of calcium homeostasis, and modulation of phonological short-term memory in language impairment (24, 25). In addition, it was shown that ATP2C2 helps colon cancer cells adapt to hypoxia, prevents cancer cells death, increases proliferation capacity and promotes tumor growth (26). ATP2C2 can serve as an independent prognostic factor and has better prediction for the survival of thyroid cancer patients (27). Therefore, ATP2C2 seems to play an antitumor role in BRCA. Here, relationship between ATP2C2 expression and TME were further studied. GSEA results revealed that high ATP2C2 expression was associated with metabolic-related signaling pathways, such as fructose and mannose metabolism, citrate cycle TCA cycle, and amino sugar and nucleotide sugar metabolism. In the ATP2C2 low-expression group, immune pathways including cytokine–cytokine receptor interaction, hematopoietic cell lineage and primary immunodeficiency were significantly enriched. Previous studies have validated that hypoxia-reprogrammed TCA cycle promotes breast tumorigenic cells growth (28). The expression of ATP2C2 is highly related to the TCA cycle, which may affect the prognosis of BRCA patients through the TCA cycle. Changes in cytosolic Ca^2+^ trigger events critical for tumorigenesis, such as cellular motility, proliferation, and apoptosis (6). Suppression of ATP2C2 attenuates basal intracellular Ca^2+^ levels and breast tumorigenicity (6). ATP2C2 can regulate Ca^2+^ metabolism and affect the prognosis of BRCA patients. Consistent with this, our analysis verified that BRCA patients with higher ATP2C2 expression had shorter OS time. In another study, the authors concluded that ATP2C2 antagonizes epithelial mesenchymal transition and suppresses BRCA cell migration and tumor metastasis (29). This study is inconsistent with our present observations and other studies (6, 7, 30). Perhaps it emphasizes that ATP2C2 can regulate epithelial to mesenchymal transition and BRCA metastasis, rather than studying the effect of ATP2C2 on the growth of BRCA. Together, our results suggested that ATP2C2 could participate in the conversion of TME from immune-dominant to metabolic-dominant status.

Considering the importance of immune cell infiltration in tumors (31–33), CIBERSORT was further applied to evaluate the abundance ratios of 22 types of immune cells in each BRCA specimen from GEO. Mounting evidence suggests that the interaction between the tumor and the microenvironment plays an important role in the progression of BRCA and immunotherapeutic efficacy. Therefore, we evaluated the potential of ATP2C2 to reflect the infiltration of immune cells. We found out the different proportions of numerous immune cells in different ATP2C2 subgroups. ATP2C2 low-expression group had significantly higher proportions of the naïve B cells, naïve CD4^+^ T cells, activated memory CD4^+^ T cells, monocytes, γδ T cells, M0 macrophages, M2 macrophages, and activated dendritic cells and significantly lower proportions of M1 macrophages, activated NK cells, and Tregs than ATP2C2 high-expression group. Tumor-infiltrating macrophages play a crucial role in tumor behavior and clinical outcome. Classically (M1) and alternatively activated (M2) macrophages exhibit different phenotypes and functions. M1 macrophages secrete cytokines, including TNF-α, IL-6, and IL-12 (34), killing tumor cells in the TME (35). M2 macrophages can secrete anti-inflammatory factors such as TGF-β and IL-10, and promote tumor growth and metastasis (36). We found that more M1 macrophages infiltrated in ATP2C2 high-expression group compared with ATP2C2 low-expression group, implying that tumor infiltrated macrophages exert immune response functions and exhibit anti-tumor effects.

With further use of LASSO Cox regression models, as a statistical method for screening cell variables to establish the TICs risk model, the predictive accuracy could be improved significantly. Moreover, a nomogram was made based on four immune infiltrating cells to establish a more accurate prognostic prediction model for BRCA. Finally, the correlation analysis revealed that there was a significantly positive correlation between Tfh cells and ATP2C2 expression, and a negative correlation between γδ T cells and ATP2C2 expression. The main function of Tfh cells is to help B cells produce antibodies. As early as 2013, Tfh cells were first found in BRCA (37). However, the role of these Tfh cells in BRCA is not clear. It is discovered that the original Tfh cells in BRCA secrete CXCL13 to promote the accumulation of Tregs in the tumor, inhibit the body’s anti-tumor immunity, and ultimately promote the development of BRCA (38). One small component of this microenvironment in humans is γδT lymphocytes, which display both innate and adaptive functions (39). Early studies have shown that γδ T lymphocytes in the BRCA cell lines have anti-tumor activity (40). Previous studies have shown that γδ T lymphocytes inhibit angiogenic signalling pathways associated with AKT and ERK, and increase apoptosis (41, 42). Interestingly, ATP2C2 promotes BRCA cell-cycle progression and cell proliferation *via* RAS-ERK pathway (6). Therefore, ATP2C2 might be responsible for the preservation of immune-active status in TME.

## Data Availability Statement

The datasets presented in this study can be found in online repositories. The names of the repository/repositories and accession number(s) can be found in the article/**Supplementary Material**.

## Author Contributions

HL and GR conceived and designed the study. JZL, YXW, and YSW searched and selected studies, analyzed the data, wrote and revised the paper. JL and JS prepared the figures and tables. All authors contributed to the article and approved the submitted version.

## Funding

This study was supported by National Natural Science Foundation of China (#31420103915, #81472475).

## Conflict of Interest

The authors declare that the research was conducted in the absence of any commercial or financial relationships that could be construed as a potential conflict of interest.
