# Supplementary material for: ATP2C2 Has Potential to Define Tumor Microenvironment in Breast Cancer
Source: Front Immunol. 2021 Apr 14;12:657950. doi: 10.3389/fimmu.2021.657950 (PMC8079766; doi:10.3389/fimmu.2021.657950)
Supplement: Supplementary file 1 [file DataSheet_1.pdf]

id	logFC	AveExpr	t	P.Value	adj.P.Val	B
HLA-DMB	1.286124	9.86216	25.8441	7.61E-99	9.11E-95	214.7438
CD2	1.944407	7.877363	24.97015	2.98E-94	1.26E-90	204.2268
SELL	2.0961	6.998906	24.96574	3.15E-94	1.26E-90	204.1737
CCL5	2.11547	8.520577	24.76508	3.58E-93	1.07E-89	201.7566
CD52	1.984791	8.337885	24.58263	3.26E-92	7.81E-89	199.5585
LCK	1.568475	6.711861	24.51093	7.78E-92	1.55E-88	198.6945
NKG7	1.951421	6.351292	24.31999	7.87E-91	1.35E-87	196.3934
IRF8	1.49214	8.384907	24.16993	4.85E-90	7.25E-87	194.5849
GZMK	2.422592	7.159408	23.57555	6.51E-87	8.66E-84	187.4217
PLEK	1.319732	7.424039	23.20234	5.98E-85	7.16E-82	182.9255
ITK	1.73523	6.159723	23.02555	5.09E-84	5.54E-81	180.7966
NCF4	1.248745	6.251626	22.82445	5.80E-83	5.79E-80	178.3757
CD247	1.572636	6.361144	22.40301	9.49E-81	8.74E-78	173.3064
PVRIG	1.484893	6.109053	22.28136	4.13E-80	3.53E-77	171.8443
RAC2	1.458748	7.951689	22.16356	1.71E-79	1.37E-76	170.4291
CD86	1.269315	6.603567	22.12797	2.63E-79	1.97E-76	170.0017
CORO1A	1.495244	8.33725	22.05342	6.47E-79	4.56E-76	169.1065
SLAMF7	1.763273	6.271421	21.95906	2.02E-78	1.34E-75	167.9738
LYZ	2.09268	9.984262	21.94597	2.37E-78	1.49E-75	167.8167
TRAT1	1.769833	4.863698	21.80352	1.32E-77	7.90E-75	166.1077
CXCL9	2.863315	9.271134	21.74136	2.79E-77	1.59E-74	165.3623
CD8A	1.619313	7.314496	21.67255	6.40E-77	3.48E-74	164.5374
IL2RB	1.484689	7.414917	21.58585	1.82E-76	9.46E-74	163.4985
IL7R	1.87331	8.160879	21.58123	1.92E-76	9.59E-74	163.4431
SLAMF8	1.570605	6.905166	21.38237	2.11E-75	1.01E-72	161.062
GIMAP4	1.507128	7.738433	21.09481	6.69E-74	3.08E-71	157.6233
IL10RA	1.312374	8.100197	20.93281	4.68E-73	2.07E-70	155.6888
STAT4	1.338085	6.23142	20.86423	1.06E-72	4.55E-70	154.8705
CST7	1.42525	6.924711	20.84516	1.34E-72	5.52E-70	154.6431
CD38	2.022663	6.259326	20.82142	1.78E-72	7.10E-70	154.3598
CCR7	1.641965	6.604924	20.78656	2.70E-72	1.04E-69	153.9442
PLAC8	2.011271	6.53997	20.77956	2.94E-72	1.10E-69	153.8608
LAPTM5	1.346842	10.47466	20.37902	3.55E-70	1.29E-67	149.0925
THEMIS2	1.111826	6.991203	20.19522	3.19E-69	1.12E-66	146.9095
LST1	0.865559	7.540641	20.1581	4.96E-69	1.70E-66	146.4689
CYTIP	1.533163	7.323165	20.09758	1.02E-68	3.39E-66	145.7512
CXCL10	2.686763	9.387481	20.03114	2.25E-68	7.29E-66	144.9636
CD27	1.36726	7.193872	20.02095	2.54E-68	8.01E-66	144.8428
BIN2	1.640771	6.142946	19.98968	3.69E-68	1.13E-65	144.4724
ARHGAP25	0.934011	6.133084	19.97062	4.63E-68	1.39E-65	144.2465
CTSS	1.507939	8.304086	19.93105	7.42E-68	2.17E-65	143.7781
FYB	1.110663	6.542518	19.90881	9.67E-68	2.76E-65	143.5147
EVI2B	1.712366	8.232598	19.80676	3.26E-67	9.06E-65	142.3074

HLA-DMA	1.354799	9.656969	19.78199	4.37E-67	1.19E-64	142.0145
C1QB	1.389893	9.444823	19.72445	8.66E-67	2.30E-64	141.3344
CSF2RB	1.602223	7.431417	19.68974	1.31E-66	3.40E-64	140.9244
LILRB1	1.144444	6.509686	19.68033	1.46E-66	3.73E-64	140.8132
HLA-DRA	1.864505	11.70093	19.5832	4.63E-66	1.16E-63	139.6666
LYN	1.125388	8.273131	19.55608	6.39E-66	1.56E-63	139.3467
LILRB2	1.208976	5.857635	19.42639	2.97E-65	7.11E-63	137.8181
GMFG	1.127517	8.317252	19.39886	4.12E-65	9.66E-63	137.4938
ADAMDEC1	2.310146	6.90541	19.23179	2.97E-64	6.84E-62	135.5284
FGL2	1.540521	8.267236	19.19163	4.78E-64	1.08E-61	135.0565
GZMB	1.990855	6.387936	19.1886	4.95E-64	1.10E-61	135.0208
GPR183	1.554543	6.979719	19.16773	6.33E-64	1.38E-61	134.7757
HLA-F	0.926742	9.622957	19.10874	1.27E-63	2.72E-61	134.0832
PTPRC	1.353641	7.140303	19.08684	1.65E-63	3.45E-61	133.8262
SH2D1A	1.083614	5.245442	19.04665	2.64E-63	5.45E-61	133.3548
CTSW	1.748638	5.502985	18.93768	9.55E-63	1.94E-60	132.0776
BTN3A3	1.167529	7.379521	18.8234	3.66E-62	7.31E-60	130.7403
LAIR1	1.157194	6.728	18.78753	5.59E-62	1.10E-59	130.3208
CD3D	1.723894	8.023247	18.73665	1.02E-61	1.96E-59	129.7263
MS4A4A	1.505719	7.178131	18.54485	9.64E-61	1.83E-58	127.4887
TRAF3IP3	1.019907	5.818837	18.35125	9.28E-60	1.74E-57	125.2361
LAMP3	1.929175	7.105349	18.34016	1.06E-59	1.95E-57	125.1073
CSF2RA	1.030156	5.589357	18.2269	3.96E-59	7.19E-57	123.7926
FPR3	1.479962	6.985719	18.19649	5.65E-59	1.01E-56	123.44
GNLY	1.537777	5.846409	18.09115	1.93E-58	3.39E-56	122.2198
ZAP70	1.204401	5.468953	18.05364	2.98E-58	5.17E-56	121.7858
GPR171	1.828738	5.585343	17.93296	1.21E-57	2.07E-55	120.3911
HCLS1	1.384999	8.454018	17.9101	1.58E-57	2.66E-55	120.1273
VAV1	0.834487	6.338725	17.88765	2.05E-57	3.41E-55	119.8682
TNFAIP3	0.985894	8.336424	17.84858	3.23E-57	5.29E-55	119.4176
SLA	1.260542	7.710599	17.8423	3.47E-57	5.55E-55	119.3452
CSF1R	1.012611	8.09538	17.84205	3.48E-57	5.55E-55	119.3423
NCKAP1L	1.02391	6.965537	17.7587	9.14E-57	1.44E-54	118.3822
SLC15A3	1.143114	7.515096	17.74943	1.02E-56	1.58E-54	118.2755
TMEM140	0.828844	7.591097	17.67213	2.49E-56	3.82E-54	117.3862
GPR18	1.733012	5.497298	17.62284	4.40E-56	6.67E-54	116.8199
IL2RG	1.606692	7.436872	17.5561	9.51E-56	1.42E-53	116.0537
CECR1	1.47761	8.404794	17.43014	4.06E-55	6.00E-53	114.6103
SPOCK2	1.067685	6.574666	17.30897	1.64E-54	2.39E-52	113.2248
BCL2A1	1.845516	6.350907	17.28903	2.06E-54	2.97E-52	112.9972
ITGB2	1.291475	7.97072	17.23921	3.64E-54	5.19E-52	112.4286
P2RX5	1.599502	5.839978	17.23603	3.78E-54	5.32E-52	112.3923
IL21R	1.08841	5.373156	17.23083	4.01E-54	5.58E-52	112.3329
UBASH3A	1.187646	4.478202	17.19815	5.83E-54	8.03E-52	111.9604

C1QA	1.591542	8.749876	17.19598	5.98E-54	8.14E-52	111.9356
LRMP	1.297627	6.067823	17.14892	1.03E-53	1.38E-51	111.3995
IDO1	1.950694	6.832901	17.13085	1.26E-53	1.68E-51	111.1938
PRKCQ	1.258384	5.388456	17.0745	2.40E-53	3.16E-51	110.5527
CASP1	1.366075	7.551502	17.05654	2.95E-53	3.84E-51	110.3486
RASSF2	1.02046	7.811549	17.04726	3.28E-53	4.22E-51	110.243
CXCL11	2.298366	6.352282	17.04198	3.49E-53	4.44E-51	110.183
PTPRCAP	1.201226	6.909064	17.01183	4.92E-53	6.20E-51	109.8406
CD79A	1.332229	6.331446	16.99488	5.97E-53	7.45E-51	109.6481
ITGA4	1.014776	5.574225	16.97638	7.38E-53	9.10E-51	109.4381
CCR2	1.20631	5.467009	16.97465	7.52E-53	9.19E-51	109.4184
DENND1C	0.767594	6.243366	16.95663	9.24E-53	1.12E-50	109.2139
PRF1	1.109908	6.678126	16.86896	2.51E-52	3.01E-50	108.2203
SASH3	0.912585	7.354997	16.80196	5.38E-52	6.38E-50	107.462
CD163	1.161455	6.989613	16.75685	8.99E-52	1.06E-49	106.9522
GPR65	1.664806	6.950557	16.6854	2.02E-51	2.35E-49	106.1455
PPP1R16B	1.198769	6.289627	16.50526	1.56E-50	1.79E-48	104.1172
IGKC	1.103012	7.004671	16.49119	1.83E-50	2.08E-48	103.9592
LCP1	1.362572	8.894776	16.41724	4.21E-50	4.75E-48	103.129
CD180	1.060592	6.288285	16.40902	4.62E-50	5.16E-48	103.0368
IL15RA	0.784237	7.435523	16.30242	1.53E-49	1.70E-47	101.8428
CXCR3	1.006129	5.622929	16.29739	1.62E-49	1.78E-47	101.7866
PSMB9	1.47861	8.967019	16.29302	1.71E-49	1.86E-47	101.7377
FYN	0.788011	7.394268	16.26566	2.32E-49	2.50E-47	101.4319
BIRC3	1.305735	6.906873	16.23387	3.32E-49	3.55E-47	101.0767
MS4A1	1.738497	5.981247	16.22505	3.66E-49	3.88E-47	100.9782
AKR1B1	0.837247	9.239688	16.16033	7.58E-49	7.95E-47	100.256
WARS	1.113613	8.9505	16.14444	9.05E-49	9.42E-47	100.0788
CPVL	1.236251	7.548229	16.14089	9.42E-49	9.72E-47	100.0393
GZMA	1.64071	6.892182	16.12583	1.12E-48	1.14E-46	99.87149
SH2B3	0.687379	7.651047	16.11909	1.20E-48	1.22E-46	99.79643
TRIM22	1.350763	9.461829	16.11405	1.27E-48	1.28E-46	99.7403
HLA-DPA1	1.367541	10.78595	16.0857	1.75E-48	1.74E-46	99.42462
IL32	1.65741	7.68651	15.96583	6.68E-48	6.61E-46	98.09238
STK10	0.63698	7.073862	15.95319	7.69E-48	7.55E-46	97.95218
GBP1	1.333718	8.932846	15.89916	1.41E-47	1.37E-45	97.35313
CD48	1.456968	7.468759	15.79252	4.61E-47	4.45E-45	96.17318
BTN3A1	0.715062	7.642172	15.74252	8.03E-47	7.69E-45	95.62093
SEMA4D	0.780878	6.854583	15.71932	1.04E-46	9.87E-45	95.36507
P2RY13	1.20024	5.284071	15.59773	4.00E-46	3.77E-44	94.02606
CCL18	2.050245	7.132253	15.58292	4.71E-46	4.40E-44	93.86329
CCL19	1.934853	8.532582	15.54961	6.80E-46	6.31E-44	93.49738
MS4A6A	1.206772	8.185482	15.54312	7.31E-46	6.73E-44	93.42603
MYO1F	0.65043	7.409432	15.53622	7.89E-46	7.21E-44	93.35038

P2RY10	1.111464	4.763593	15.46426	1.75E-45	1.58E-43	92.56125
TLR1	1.163477	6.195387	15.46169	1.80E-45	1.62E-43	92.53307
FAM65B	1.012095	5.741029	15.44871	2.07E-45	1.85E-43	92.39088
HLA-B	1.004054	12.72965	15.42414	2.72E-45	2.41E-43	92.12195
HLA-DOB	1.06757	5.657835	15.42299	2.75E-45	2.42E-43	92.10935
HLA-G	1.113071	10.74949	15.38303	4.27E-45	3.73E-43	91.67228
TNFRSF1B	0.928532	7.731856	15.35849	5.59E-45	4.85E-43	91.40411
CTSC	1.23915	8.815854	15.3549	5.82E-45	5.01E-43	91.36485
TAP1	1.183593	9.398516	15.29903	1.07E-44	9.19E-43	90.75515
CD19	1.271773	5.278504	15.26982	1.48E-44	1.26E-42	90.43667
PSTPIP1	1.0896	5.593223	15.26605	1.54E-44	1.30E-42	90.39563
PLEKHO2	0.664096	8.101243	15.26031	1.64E-44	1.38E-42	90.33307
RNASE6	1.471034	7.625096	15.22363	2.46E-44	2.04E-42	89.93363
IGK	1.986458	6.551243	15.16774	4.53E-44	3.74E-42	89.32594
LGALS2	1.413723	5.496478	15.14532	5.79E-44	4.74E-42	89.08234
BCL11B	0.921419	6.066316	15.11028	8.49E-44	6.91E-42	88.70203
NCF2	1.08103	7.178926	15.1057	8.92E-44	7.21E-42	88.65238
GIMAP6	1.177868	7.718523	15.05685	1.52E-43	1.22E-41	88.12293
ICOS	1.353566	4.824563	15.05018	1.63E-43	1.30E-41	88.05068
CD14	0.983819	9.505546	15.04353	1.76E-43	1.39E-41	87.97868
PIM2	1.331166	6.771044	15.02789	2.08E-43	1.64E-41	87.80945
NPL	0.827335	6.693349	14.98719	3.25E-43	2.54E-41	87.36929
ACSL5	0.871996	7.098747	14.98339	3.38E-43	2.63E-41	87.3282
TFEC	1.151264	5.637968	14.94255	5.27E-43	4.07E-41	86.88704
BTK	0.856034	6.195699	14.94079	5.38E-43	4.12E-41	86.86801
ADA	0.813293	6.772457	14.88375	9.98E-43	7.61E-41	86.2529
ICAM2	0.826148	7.859025	14.81657	2.07E-42	1.57E-40	85.52976
LTB	0.962557	6.879059	14.79775	2.53E-42	1.91E-40	85.32744
HCK	0.868571	7.491012	14.79044	2.74E-42	2.05E-40	85.24886
C3AR1	0.997609	8.517678	14.75129	4.19E-42	3.11E-40	84.82846
BTN3A2	1.132994	7.966241	14.7431	4.58E-42	3.38E-40	84.74062
FGR	0.721335	6.721592	14.66904	1.02E-41	7.47E-40	83.94692
IPCEF1	0.919222	5.589347	14.65417	1.19E-41	8.67E-40	83.78773
MAP4K1	1.070667	5.857263	14.65403	1.20E-41	8.67E-40	83.78631
MX2	1.067127	8.136261	14.64333	1.34E-41	9.67E-40	83.67178
CXCR6	0.827438	5.559232	14.63129	1.53E-41	1.09E-39	83.54307
IKZF1	0.648326	5.254925	14.56214	3.21E-41	2.29E-39	82.80456
BANK1	1.15536	5.116789	14.54883	3.71E-41	2.62E-39	82.66261
HLA-A	0.662152	13.29201	14.50087	6.20E-41	4.36E-39	82.15154
STAT1	1.267198	8.895016	14.48661	7.22E-41	5.06E-39	81.99972
GPSM3	0.808285	6.707945	14.47799	7.92E-41	5.51E-39	81.90803
PNOC	1.182805	5.24906	14.4643	9.17E-41	6.35E-39	81.76236
EMP3	1.03158	8.489506	14.33197	3.77E-40	2.59E-38	80.35818
LAG3	1.1498	4.808974	14.32594	4.02E-40	2.75E-38	80.29437

XCL1	0.755232	5.024408	14.27213	7.13E-40	4.85E-38	79.72519
LAX1	1.132856	6.347219	14.24954	9.07E-40	6.13E-38	79.48659
FCER1G	1.402688	8.672303	14.24353	9.67E-40	6.50E-38	79.42318
MRC1	1.449131	7.329689	14.2318	1.10E-39	7.32E-38	79.29931
BIN1	0.720916	7.055811	14.21338	1.33E-39	8.86E-38	79.10504
SLC7A7	0.989836	8.27169	14.20266	1.49E-39	9.87E-38	78.99198
TYROBP	1.054129	9.839423	14.19952	1.54E-39	1.01E-37	78.9589
SIGLEC1	0.942674	6.546926	14.18936	1.72E-39	1.12E-37	78.85182
CRTAM	1.254426	5.055819	14.14131	2.86E-39	1.86E-37	78.34597
SRGN	1.170669	9.298116	14.11799	3.66E-39	2.37E-37	78.1007
CMKLR1	0.779425	5.822935	14.11639	3.72E-39	2.40E-37	78.08394
CCL2	1.492287	8.79973	14.11385	3.83E-39	2.45E-37	78.05721
PLEKHO1	0.957817	7.801611	14.09625	4.61E-39	2.93E-37	77.8723
CLEC4A	0.986694	5.959257	14.08957	4.95E-39	3.13E-37	77.80212
CXCL13	2.580152	7.689947	14.06351	6.51E-39	4.10E-37	77.52859
TNFRSF17	2.069853	5.496112	14.02096	1.02E-38	6.40E-37	77.08253
CCR1	1.004437	7.305346	14.00031	1.27E-38	7.91E-37	76.86632
NCF1	0.956161	6.589617	13.94627	2.24E-38	1.39E-36	76.30109
HLA-E	0.711944	11.22901	13.89954	3.66E-38	2.26E-36	75.81335
MPP1	0.794593	7.602457	13.89643	3.79E-38	2.32E-36	75.78093
LY9	0.916464	5.121926	13.89118	4.00E-38	2.44E-36	75.72614
PILRA	0.626172	6.21754	13.86313	5.37E-38	3.26E-36	75.43385
GZMH	1.217444	5.646569	13.77481	1.35E-37	8.19E-36	74.51534
CD3G	1.234919	5.692021	13.76047	1.57E-37	9.46E-36	74.36647
WAS	0.545056	6.803692	13.75006	1.75E-37	1.05E-35	74.25839
SH2D2A	1.1601	5.839148	13.74882	1.78E-37	1.06E-35	74.24558
TCN2	0.917763	6.975998	13.7459	1.83E-37	1.09E-35	74.21534
CD72	1.053422	5.472937	13.73436	2.07E-37	1.22E-35	74.09558
CD40	0.598824	6.29912	13.72938	2.18E-37	1.28E-35	74.04398
CD3E	1.446483	5.95934	13.6871	3.38E-37	1.98E-35	73.60612
TLR7	1.021067	6.224212	13.67077	4.01E-37	2.33E-35	73.43719
RASSF4	0.774789	6.110873	13.63844	5.62E-37	3.25E-35	73.10296
KLRB1	1.318134	6.156242	13.49143	2.58E-36	1.49E-34	71.58862
PRKCB	0.841184	5.65728	13.45406	3.80E-36	2.18E-34	71.20505
TCL1A	1.1808	5.239016	13.38289	7.92E-36	4.51E-34	70.47603
KIAA0125	0.962828	4.682209	13.36316	9.71E-36	5.51E-34	70.27428
HCP5	1.163635	7.396144	13.31805	1.54E-35	8.72E-34	69.81362
PSAP	0.701581	11.97065	13.30599	1.75E-35	9.82E-34	69.69068
FAM49A	0.705293	6.227763	13.27259	2.46E-35	1.38E-33	69.35035
SELPLG	0.822534	7.096908	13.27121	2.50E-35	1.39E-33	69.33621
IGFLR1	0.657824	7.488918	13.24111	3.40E-35	1.88E-33	69.02996
SAMHD1	0.76463	8.236121	13.211	4.63E-35	2.55E-33	68.72389
ADCY7	0.663566	6.800824	13.1848	6.05E-35	3.32E-33	68.45788
AOAH	0.969786	6.777379	13.17241	6.87E-35	3.75E-33	68.33222

ALOX5AP	1.472606	8.579316	13.16674	7.28E-35	3.96E-33	68.2747
IRF1	0.832871	8.185441	13.13688	9.87E-35	5.35E-33	67.97212
RUNX3	0.835589	6.853039	13.13257	1.03E-34	5.56E-33	67.92846
PTAFR	0.731815	6.643397	13.13031	1.06E-34	5.67E-33	67.90562
PLA2G7	1.299617	7.284885	13.08054	1.75E-34	9.37E-33	67.4022
PLA2G2D	1.281648	5.113293	13.07167	1.92E-34	1.02E-32	67.3126
P2RY14	0.883306	6.840744	13.05916	2.18E-34	1.15E-32	67.18628
COTL1	0.887039	8.318114	12.99079	4.36E-34	2.30E-32	66.49713
FAS	0.811029	7.085151	12.98566	4.60E-34	2.41E-32	66.44556
TBX21	0.597728	5.529436	12.96055	5.93E-34	3.10E-32	66.19296
IFI44L	1.607878	8.886971	12.95954	5.99E-34	3.12E-32	66.1828
SIT1	0.970395	5.615921	12.95734	6.13E-34	3.17E-32	66.1607
CLIC2	1.115816	6.722283	12.92013	8.93E-34	4.60E-32	65.78704
SYK	0.746175	6.61529	12.91801	9.12E-34	4.68E-32	65.7657
SLAMF1	0.876609	5.133441	12.91176	9.71E-34	4.97E-32	65.70305
DOK2	0.829193	6.503698	12.90637	1.03E-33	5.22E-32	65.64893
RHOG	0.614753	8.860682	12.797	3.09E-33	1.57E-31	64.55469
SERPINB9	0.710765	6.532165	12.79269	3.23E-33	1.63E-31	64.5116
PARP8	0.853797	6.949741	12.7598	4.49E-33	2.26E-31	64.18363
ZBED2	1.073191	4.782919	12.75125	4.90E-33	2.45E-31	64.09843
IL16	0.657079	6.13015	12.71736	6.88E-33	3.43E-31	63.76117
HPSE	0.995759	5.453117	12.71257	7.22E-33	3.58E-31	63.71353
EPHB2	0.556606	5.804422	12.66117	1.21E-32	5.97E-31	63.20293
TLR2	1.067154	7.582062	12.64173	1.47E-32	7.22E-31	63.0101
CCDC69	0.835204	6.213028	12.59045	2.45E-32	1.19E-30	62.50235
APOL6	0.865557	6.92682	12.55564	3.46E-32	1.68E-30	62.1584
KLRD1	0.870558	4.933373	12.49834	6.12E-32	2.95E-30	61.59332
FCN1	0.880849	5.804245	12.49833	6.12E-32	2.95E-30	61.59326
STAP1	1.000277	4.699849	12.4506	9.82E-32	4.72E-30	61.12374
AIM2	1.280237	6.263361	12.41233	1.43E-31	6.86E-30	60.74802
CLEC10A	0.848806	6.273214	12.39382	1.72E-31	8.21E-30	60.56654
PTGDS	1.148188	8.212446	12.37014	2.18E-31	1.03E-29	60.3345
KIF21B	1.128096	5.076025	12.32696	3.33E-31	1.58E-29	59.9123
CTSL	0.698114	9.75692	12.2327	8.41E-31	3.96E-29	58.99347
IL18	1.042361	6.026139	12.2056	1.10E-30	5.15E-29	58.73011
RPS6KA3	0.755435	7.727269	12.18262	1.37E-30	6.42E-29	58.507
HLA-C	0.724905	12.60462	12.14284	2.03E-30	9.44E-29	58.12145
KIAA0226L	0.785855	6.268882	12.10755	2.86E-30	1.33E-28	57.78007
CD5	0.651819	5.837857	12.05337	4.85E-30	2.24E-28	57.25701
CD96	1.078034	5.138617	12.01772	6.85E-30	3.15E-28	56.91363
SLCO2B1	0.989582	6.62974	12.00234	7.95E-30	3.65E-28	56.76573
CD1E	0.765425	5.036696	11.98775	9.16E-30	4.18E-28	56.62551
CD69	1.221423	6.448771	11.88979	2.36E-29	1.07E-27	55.68659
IGHM	1.997345	7.975014	11.88877	2.39E-29	1.08E-27	55.67683

CCRL2	0.877147	5.411484	11.86884	2.89E-29	1.31E-27	55.4864
FLI1	0.600654	5.773134	11.83365	4.06E-29	1.82E-27	55.15055
VSIG4	0.865693	7.911736	11.83245	4.10E-29	1.84E-27	55.13918
TOX	0.725638	4.882574	11.80603	5.29E-29	2.36E-27	54.88751
ITGAL	0.95259	6.482961	11.80454	5.36E-29	2.39E-27	54.87332
OVOL2	-0.54336	7.043521	-11.8015	5.52E-29	2.45E-27	54.84471
CXCR4	0.782896	9.034791	11.78015	6.78E-29	2.99E-27	54.64131
MNDA	1.187497	7.561543	11.7781	6.91E-29	3.04E-27	54.62181
C5AR1	0.96115	6.558453	11.74397	9.59E-29	4.20E-27	54.29765
PLEKHF1	0.884968	7.017451	11.73261	1.07E-28	4.67E-27	54.18989
CTLA4	0.690486	4.706124	11.72525	1.15E-28	4.99E-27	54.12011
AIF1	0.816238	7.738463	11.71376	1.28E-28	5.55E-27	54.01127
SEL1L3	1.051726	6.590429	11.65982	2.14E-28	9.22E-27	53.50092
SP110	0.671484	8.253479	11.59664	3.91E-28	1.68E-26	52.90516
RARRES1	1.621038	8.085224	11.57412	4.84E-28	2.07E-26	52.69321
6-Sep	0.727741	6.918112	11.526	7.63E-28	3.24E-26	52.24141
ARPC1B	0.683998	10.57326	11.51456	8.51E-28	3.60E-26	52.13408
MARCKS	0.639432	9.456232	11.49972	9.79E-28	4.12E-26	51.99512
PTPN22	0.67114	4.483579	11.49136	1.06E-27	4.45E-26	51.91678
ITM2C	0.83406	8.354065	11.41011	2.28E-27	9.50E-26	51.15795
RGL2	-0.60513	9.320523	-11.4055	2.38E-27	9.89E-26	51.1146
KIAA0556	-0.56517	7.844217	-11.3718	3.26E-27	1.35E-25	50.80166
ME2	0.800116	7.897878	11.37124	3.28E-27	1.35E-25	50.79612
CD84	0.689672	5.745122	11.37065	3.30E-27	1.36E-25	50.7906
ITGAM	0.730692	7.641199	11.32713	4.96E-27	2.03E-25	50.38646
APOE	0.692795	9.554045	11.3219	5.21E-27	2.13E-25	50.33798
CD37	1.53692	6.114857	11.31897	5.36E-27	2.18E-25	50.31076
SH3BGRL3	0.664088	9.550418	11.31744	5.43E-27	2.20E-25	50.2966
OAS2	0.922362	6.922851	11.31548	5.54E-27	2.24E-25	50.27841
NAGK	0.581778	10.04048	11.3101	5.82E-27	2.35E-25	50.22854
KDM4B	-0.67483	7.473402	-11.2931	6.82E-27	2.74E-25	50.07135
MAPT	-1.12874	6.720071	-11.2382	1.14E-26	4.56E-25	49.56367
CD28	0.51616	4.537355	11.22398	1.30E-26	5.19E-25	49.43252
DLG5	-0.80062	8.168199	-11.2129	1.44E-26	5.73E-25	49.33036
HHAT	-0.84595	6.640451	-11.2111	1.47E-26	5.81E-25	49.31385
APOBEC3C	0.531389	7.15226	11.18829	1.81E-26	7.16E-25	49.10371
CFLAR	0.544311	7.42684	11.08761	4.60E-26	1.81E-24	48.17994
P2RX7	0.711253	5.917433	11.08258	4.82E-26	1.89E-24	48.13396
RAB8B	0.599152	7.101873	11.03646	7.38E-26	2.89E-24	47.71266
AQP9	1.266872	5.184195	11.03182	7.70E-26	3.00E-24	47.67041
IRS1	-0.914	7.308637	-11.0315	7.72E-26	3.00E-24	47.66739
SIRPG	0.884937	4.759387	11.01764	8.78E-26	3.40E-24	47.5411
IFI16	0.702596	9.782539	11.00343	1.00E-25	3.86E-24	47.41171
SAMSN1	0.869591	6.157614	10.99308	1.10E-25	4.23E-24	47.31758

PAX5	0.676333	5.65993	10.98007	1.24E-25	4.75E-24	47.19927
EFHC1	-0.65296	7.125399	-10.9779	1.26E-25	4.84E-24	47.17919
PTGER4	0.743872	6.864432	10.93938	1.80E-25	6.86E-24	46.8298
LIPA	0.821505	10.23624	10.91355	2.28E-25	8.63E-24	46.59573
LY96	1.162383	8.279378	10.88457	2.97E-25	1.12E-23	46.3336
ATP6V1B2	0.824185	9.206137	10.87872	3.13E-25	1.18E-23	46.28073
ADRB2	0.792224	6.486513	10.83618	4.62E-25	1.73E-23	45.89689
MAP3K14	0.593374	6.128145	10.83229	4.78E-25	1.79E-23	45.86187
FLNB	-0.72013	9.520844	-10.8208	5.31E-25	1.98E-23	45.758
SMCO4	0.883662	8.674454	10.8127	5.72E-25	2.12E-23	45.68547
TLR4	0.869206	5.980448	10.80883	5.92E-25	2.19E-23	45.65062
MFHAS1	0.74118	7.388941	10.80085	6.37E-25	2.35E-23	45.57887
TNF	0.854124	5.273787	10.77213	8.26E-25	3.04E-23	45.32082
PCLO	-0.71806	5.213441	-10.7706	8.37E-25	3.07E-23	45.30755
CD1D	0.873677	5.046299	10.76376	8.91E-25	3.26E-23	45.24577
C1S	1.120193	9.823386	10.73572	1.15E-24	4.18E-23	44.99441
APOL3	0.757096	7.569849	10.71996	1.32E-24	4.80E-23	44.85332
RSAD2	1.222645	7.763392	10.71663	1.37E-24	4.94E-23	44.82354
PTPN7	0.660127	5.762142	10.7119	1.42E-24	5.14E-23	44.78125
FCGR2B	1.38586	7.527058	10.69668	1.63E-24	5.88E-23	44.64521
RRNAD1	-0.64055	7.257051	-10.6416	2.68E-24	9.62E-23	44.15405
ST8SIA4	0.670472	5.493691	10.6264	3.08E-24	1.10E-22	44.0187
ZMYND10	-0.63996	5.660713	-10.6254	3.11E-24	1.11E-22	44.00957
CD40LG	0.880151	4.645338	10.59505	4.08E-24	1.44E-22	43.74017
RAB26	-0.87559	6.920704	-10.5791	4.71E-24	1.66E-22	43.59869
ARRB2	0.932835	6.568259	10.48233	1.12E-23	3.93E-22	42.74328
IL2RA	0.676706	5.151479	10.48002	1.14E-23	4.00E-22	42.72293
CCL8	1.401343	7.604182	10.46989	1.25E-23	4.37E-22	42.63373
IGLL3P	1.259523	9.768072	10.45819	1.38E-23	4.83E-22	42.53071
STK17A	0.60778	6.64477	10.45059	1.48E-23	5.15E-22	42.46387
DPEP2	0.901483	4.878161	10.43876	1.65E-23	5.71E-22	42.35986
EGFL6	1.18421	6.54786	10.43612	1.68E-23	5.83E-22	42.33672
ASTN2	-0.67878	5.625031	-10.4346	1.71E-23	5.89E-22	42.32339
DACH1	-1.684	6.860367	-10.3951	2.42E-23	8.31E-22	41.97655
PIP4K2A	0.601689	7.431925	10.38958	2.54E-23	8.70E-22	41.92843
OAS1	0.92285	7.811822	10.36979	3.03E-23	1.03E-21	41.75526
CD1C	0.957339	5.984288	10.3527	3.53E-23	1.20E-21	41.60579
ATP8B1	-0.8117	8.888248	-10.3341	4.15E-23	1.40E-21	41.44347
ICAM1	0.821498	7.000355	10.33272	4.21E-23	1.42E-21	41.43135
CACNA1D	-1.01101	6.01591	-10.3046	5.39E-23	1.81E-21	41.18587
RELB	0.64552	6.549376	10.30425	5.40E-23	1.81E-21	41.18317
ENPP2	1.002582	8.315384	10.30216	5.50E-23	1.84E-21	41.16495
FLVCR2	0.652158	5.656633	10.29763	5.73E-23	1.91E-21	41.12553
IFNG	0.901043	4.732764	10.2902	6.12E-23	2.03E-21	41.06088

GLIPR1	0.830178	8.068217	10.24852	8.82E-23	2.91E-21	40.69878
SPIB	0.917051	5.220852	10.23323	1.01E-22	3.32E-21	40.56614
LCP2	0.979199	6.548532	10.1682	1.78E-22	5.84E-21	40.00383
BLK	0.530548	4.481943	10.16582	1.82E-22	5.93E-21	39.98327
CCL11	0.758599	5.560795	10.16329	1.86E-22	6.03E-21	39.96147
C1R	0.727101	10.08006	10.15517	1.99E-22	6.45E-21	39.89138
MYB	-1.02324	6.872349	-10.1388	2.30E-22	7.42E-21	39.74995
SLC31A2	0.609727	7.985464	10.12367	2.62E-22	8.44E-21	39.62019
GYPC	0.74073	8.043856	10.10969	2.96E-22	9.51E-21	39.49992
SLC9A6	0.599366	7.602089	10.0993	3.24E-22	1.04E-20	39.41069
ERBB4	-1.22196	6.204085	-10.0914	3.47E-22	1.11E-20	39.34329
PDCD1LG2	0.824373	4.479941	10.08731	3.60E-22	1.15E-20	39.30777
CCND1	-1.04875	9.543378	-10.0747	4.02E-22	1.27E-20	39.19936
IRF9	0.850929	9.0503	10.07103	4.14E-22	1.31E-20	39.16815
FXYD5	0.789276	9.203715	10.04525	5.18E-22	1.64E-20	38.9474
RAB29	0.579322	7.392771	10.02457	6.20E-22	1.95E-20	38.77057
PLTP	0.915969	8.314298	10.01451	6.76E-22	2.12E-20	38.68474
DRAM1	0.582665	8.043196	10.0049	7.34E-22	2.29E-20	38.60271
LGMN	0.845017	9.833427	9.956423	1.11E-21	3.47E-20	38.18984
P2RY6	0.683188	6.411158	9.948785	1.19E-21	3.70E-20	38.12491
IL15	0.70774	5.6687	9.919854	1.53E-21	4.73E-20	37.87934
NEDD4L	-0.79257	7.269003	-9.91393	1.61E-21	4.96E-20	37.82915
CSPP1	-0.55454	6.455561	-9.87243	2.29E-21	7.05E-20	37.47793
MMP9	1.280575	9.195613	9.864776	2.45E-21	7.50E-20	37.41323
KIAA0895	-0.80925	6.053871	-9.86185	2.51E-21	7.67E-20	37.38856
IGLJ3	1.671249	7.400054	9.846917	2.85E-21	8.69E-20	37.26249
LIMD2	1.016412	5.398586	9.82526	3.43E-21	1.04E-19	37.07996
MAGED2	-0.68782	9.723465	-9.82478	3.44E-21	1.04E-19	37.07589
MALT1	0.568113	6.790268	9.808569	3.95E-21	1.20E-19	36.93948
TRIM45	-0.69102	6.878089	-9.80298	4.14E-21	1.25E-19	36.89244
LRRC8E	-0.60733	6.42579	-9.79827	4.31E-21	1.30E-19	36.85287
PNRC1	0.699859	9.82703	9.795634	4.41E-21	1.33E-19	36.83073
KCNAB2	0.541981	5.94592	9.789879	4.63E-21	1.39E-19	36.78238
FAAH	-0.6927	6.711078	-9.78713	4.74E-21	1.41E-19	36.75927
ZNF587B	-0.64162	8.403257	-9.77927	5.07E-21	1.51E-19	36.69332
IKZF3	0.843273	5.103238	9.762277	5.85E-21	1.74E-19	36.55075
TRAF1	0.560502	5.374989	9.752417	6.36E-21	1.88E-19	36.46812
ITGB7	0.858447	6.273489	9.749696	6.51E-21	1.92E-19	36.44533
SGPP1	0.70515	6.88505	9.732382	7.54E-21	2.22E-19	36.30041
KIAA0232	-0.6551	8.28808	-9.73008	7.69E-21	2.26E-19	36.28113
STC2	-1.4166	8.093765	-9.64179	1.62E-20	4.74E-19	35.5452
APOBEC3G	0.844839	6.577476	9.636888	1.69E-20	4.92E-19	35.50449
PHACTR1	0.514436	5.144699	9.627326	1.83E-20	5.32E-19	35.42511
CHIT1	1.049594	5.163226	9.62038	1.94E-20	5.63E-19	35.36748

CELF2	0.597008	7.087281	9.618661	1.97E-20	5.70E-19	35.35321
SPEF1	-0.55364	5.938259	-9.61254	2.07E-20	5.98E-19	35.30245
UBE2L6	0.653173	9.952229	9.60869	2.14E-20	6.16E-19	35.27055
TMEM176A	0.783925	8.939823	9.597152	2.35E-20	6.77E-19	35.17497
ELOVL2	-1.06754	5.67572	-9.58244	2.66E-20	7.64E-19	35.05321
SECTM1	0.696762	7.659016	9.563078	3.13E-20	8.97E-19	34.8932
CCR5	1.197206	7.556884	9.560982	3.19E-20	9.10E-19	34.87589
IL10RB	0.596417	8.043628	9.560236	3.21E-20	9.14E-19	34.86973
HSD11B1	0.756646	5.969453	9.557696	3.28E-20	9.31E-19	34.84876
MAST4	-0.65457	7.154633	-9.54837	3.54E-20	1.00E-18	34.7718
TIGD6	-0.73317	4.32891	-9.54265	3.71E-20	1.05E-18	34.72459
CD300A	0.584807	5.474948	9.529663	4.14E-20	1.17E-18	34.61758
IRX5	-0.82755	9.133433	-9.52469	4.31E-20	1.21E-18	34.57667
IFI27	1.141515	10.35457	9.494641	5.54E-20	1.56E-18	34.32947
IFT140	-0.52484	5.563155	-9.49093	5.71E-20	1.60E-18	34.29901
GBP2	0.666696	8.818895	9.467177	6.96E-20	1.94E-18	34.10408
SEMA3F	-0.56281	7.176694	-9.45458	7.73E-20	2.15E-18	34.00083
MZB1	1.051813	7.340408	9.453604	7.79E-20	2.16E-18	33.99286
FMO5	-0.91279	5.434598	-9.38537	1.37E-19	3.79E-18	33.43555
CCL21	1.034266	6.549081	9.378692	1.45E-19	3.99E-18	33.38116
AGBL2	-0.69516	4.835022	-9.37564	1.48E-19	4.08E-18	33.35634
AKR7A3	-0.79752	7.986906	-9.35625	1.74E-19	4.78E-18	33.19864
MARCO	1.124456	5.287798	9.342913	1.94E-19	5.32E-18	33.09027
LAP3	0.728705	10.41726	9.325899	2.23E-19	6.11E-18	32.95223
ZNF606	-0.54604	7.015331	-9.30331	2.69E-19	7.33E-18	32.76923
ERBB3	-0.55506	7.316523	-9.25248	4.08E-19	1.11E-17	32.35867
CLSTN2	-1.34242	6.824158	-9.2308	4.86E-19	1.32E-17	32.1841
FAM63A	-0.72378	8.652428	-9.2183	5.39E-19	1.45E-17	32.08358
TNFAIP8	0.760719	7.510889	9.217987	5.40E-19	1.45E-17	32.08107
APOL1	0.709831	7.303695	9.203219	6.09E-19	1.63E-17	31.96244
DZANK1	-0.65663	5.77918	-9.18253	7.20E-19	1.92E-17	31.79645
PLXNC1	0.543143	6.953429	9.17744	7.51E-19	2.00E-17	31.75569
FOLR2	0.653393	6.549024	9.170517	7.94E-19	2.11E-17	31.70024
KRT8	-0.83822	11.02682	-9.16657	8.20E-19	2.17E-17	31.66864
LAMB2	-0.77368	8.948108	-9.16367	8.39E-19	2.22E-17	31.64546
FAM174B	-0.82025	7.9194	-9.16284	8.45E-19	2.23E-17	31.63881
KLRG1	0.876644	5.75025	9.160582	8.61E-19	2.26E-17	31.62072
ISG20	0.891059	8.189343	9.157695	8.81E-19	2.31E-17	31.59763
SLC19A2	-0.96547	8.687517	-9.15738	8.83E-19	2.31E-17	31.59511
INPP5D	0.814866	5.787248	9.15421	9.06E-19	2.37E-17	31.56975
MMP16	-0.50685	4.973092	-9.13253	1.08E-18	2.82E-17	31.39652
TAP2	0.630781	5.950824	9.132232	1.08E-18	2.82E-17	31.39417
C11orf21	0.550321	5.100962	9.121792	1.18E-18	3.06E-17	31.31088
IL1R2	0.73871	4.947157	9.108155	1.32E-18	3.40E-17	31.20218

JUP	-0.56918	9.641677	-9.0966	1.44E-18	3.72E-17	31.11016
RASGRP1	0.895535	7.477232	9.087104	1.56E-18	4.01E-17	31.03463
DNAJC12	-1.35418	7.63508	-9.06547	1.86E-18	4.76E-17	30.86273
JAK2	0.568431	5.874825	9.059888	1.94E-18	4.96E-17	30.81844
CD33	0.810044	5.337465	9.046584	2.16E-18	5.51E-17	30.71294
ELMO1	0.684619	6.61512	9.039531	2.29E-18	5.82E-17	30.65706
ABCA3	-0.70373	7.36499	-9.03629	2.35E-18	5.96E-17	30.63138
NFKB1	0.544534	8.336646	9.035777	2.36E-18	5.97E-17	30.62733
FGFR3	-0.78548	5.794196	-9.02049	2.66E-18	6.72E-17	30.50637
RNF43	-0.77309	6.894295	-8.98747	3.47E-18	8.72E-17	30.24557
THSD4	-0.91675	5.675904	-8.98683	3.49E-18	8.74E-17	30.24056
HLA-J	1.031172	10.3547	8.979678	3.69E-18	9.22E-17	30.18415
LAMA5	-0.65494	8.292193	-8.97527	3.82E-18	9.53E-17	30.14942
MILR1	0.613459	5.308711	8.973065	3.89E-18	9.68E-17	30.13205
USP18	0.710238	7.350474	8.959889	4.32E-18	1.07E-16	30.02832
GATA3	-1.36119	10.4147	-8.95943	4.34E-18	1.07E-16	30.02469
TRIM21	0.537993	7.308346	8.952121	4.60E-18	1.14E-16	29.96722
PTOV1	-0.50559	9.021933	-8.95132	4.63E-18	1.14E-16	29.96094
VPREB3	0.577317	5.265812	8.944404	4.89E-18	1.20E-16	29.90656
KCND3	-0.59362	4.97647	-8.9435	4.93E-18	1.21E-16	29.89943
VCAM1	0.980051	8.92874	8.932404	5.38E-18	1.32E-16	29.81231
RTP4	1.037246	7.049217	8.930368	5.47E-18	1.34E-16	29.79633
FASLG	0.555264	4.83097	8.924134	5.75E-18	1.40E-16	29.74742
ABAT	-0.92908	7.294141	-8.92155	5.86E-18	1.43E-16	29.72716
PRDM1	0.741448	6.699525	8.917611	6.05E-18	1.47E-16	29.69627
OASL	0.788834	6.782762	8.917405	6.06E-18	1.47E-16	29.69465
NFE2L3	0.680044	6.329605	8.913839	6.24E-18	1.51E-16	29.6667
KIAA1107	-0.5065	5.458963	-8.91166	6.34E-18	1.53E-16	29.64961
C10orf10	0.605822	7.802361	8.899268	7.00E-18	1.68E-16	29.55257
CEP83	-0.50049	5.683326	-8.8928	7.37E-18	1.76E-16	29.50197
ZNF587	-0.64411	8.692127	-8.8662	9.10E-18	2.15E-16	29.29408
IFIT3	1.101591	8.440376	8.866132	9.10E-18	2.15E-16	29.29356
OLR1	1.110674	6.311515	8.864309	9.23E-18	2.18E-16	29.27933
LAIR2	0.504917	5.912622	8.863212	9.31E-18	2.19E-16	29.27077
PLAUR	0.570497	7.575153	8.845001	1.08E-17	2.52E-16	29.12878
SDC4	-0.58399	10.45082	-8.82992	1.21E-17	2.84E-16	29.01138
CASZ1	-0.57291	6.762707	-8.80453	1.48E-17	3.44E-16	28.81401
IFI44	0.817998	7.365636	8.79544	1.59E-17	3.69E-16	28.74346
MAST1	0.623394	4.365393	8.769529	1.95E-17	4.49E-16	28.54267
STAG3	0.586325	5.485549	8.76914	1.95E-17	4.50E-16	28.53966
PYHIN1	0.555012	5.058842	8.765577	2.01E-17	4.62E-16	28.51209
ST6GALNAC2	-0.95217	8.133598	-8.7633	2.05E-17	4.69E-16	28.49444
TNFSF14	0.563093	4.712529	8.760906	2.09E-17	4.77E-16	28.47595
PRKX	0.703125	6.652168	8.743394	2.39E-17	5.45E-16	28.34061

PLA1A	0.742366	5.201216	8.740284	2.45E-17	5.58E-16	28.3166
ARNTL2	0.845788	5.459252	8.736481	2.53E-17	5.73E-16	28.28724
IL18R1	0.617355	4.944204	8.726438	2.73E-17	6.18E-16	28.20976
POLI	-0.62366	7.04843	-8.72334	2.80E-17	6.32E-16	28.1859
P4HTM	-0.68085	8.60651	-8.70117	3.33E-17	7.49E-16	28.01515
HLA-DQB2	0.835968	7.059134	8.700378	3.35E-17	7.52E-16	28.00904
XAF1	0.764924	7.9887	8.685278	3.77E-17	8.43E-16	27.89295
ARHGAP32	-0.86682	7.273874	-8.6689	4.28E-17	9.54E-16	27.76719
FBXW4	-0.5295	8.752695	-8.66588	4.38E-17	9.75E-16	27.74401
ZNF500	-0.54963	6.692638	-8.66512	4.41E-17	9.79E-16	27.73821
IL18RAP	0.590357	5.189676	8.658025	4.66E-17	1.03E-15	27.68382
LSP1	0.523001	7.633699	8.614665	6.52E-17	1.43E-15	27.35212
HK3	0.748709	5.062635	8.613408	6.58E-17	1.44E-15	27.34253
SLC39A6	-0.97813	10.36094	-8.59491	7.60E-17	1.66E-15	27.20143
PSMB8	0.952142	9.042488	8.593704	7.67E-17	1.67E-15	27.19225
LRP2	-1.29924	6.727776	-8.57958	8.55E-17	1.86E-15	27.08466
RNF19B	0.956385	6.980945	8.5662	9.48E-17	2.06E-15	26.98292
TLR8	0.87393	4.601072	8.560838	9.88E-17	2.14E-15	26.94216
BCL11A	0.960395	6.598435	8.557922	1.01E-16	2.18E-15	26.92001
EBI3	0.570978	5.237621	8.536499	1.19E-16	2.57E-15	26.75745
ODC1	0.767327	9.236884	8.533514	1.22E-16	2.63E-15	26.73483
WIPF1	0.797093	7.515518	8.512891	1.43E-16	3.07E-15	26.57867
RAB33A	0.531282	4.992919	8.511696	1.44E-16	3.09E-15	26.56963
SHANK2	-0.5698	6.403834	-8.47639	1.89E-16	4.03E-15	26.30304
TFF3	-1.62947	9.011572	-8.47219	1.95E-16	4.15E-15	26.27136
ADAM28	0.582437	5.480543	8.471158	1.97E-16	4.18E-15	26.26358
C5orf30	-0.87473	7.941355	-8.4702	1.98E-16	4.20E-15	26.25637
PPP1R13B	-0.53834	7.475697	-8.46912	2.00E-16	4.23E-15	26.24823
SOAT1	0.762311	6.992984	8.464129	2.08E-16	4.38E-15	26.21063
GLI3	-0.64765	7.77984	-8.46394	2.08E-16	4.38E-15	26.20919
ZNF223	-0.66468	5.461581	-8.46092	2.13E-16	4.47E-15	26.18646
DYNC2LI1	-0.55814	6.38083	-8.44781	2.35E-16	4.92E-15	26.08785
MYO5C	-0.77971	9.195867	-8.44313	2.44E-16	5.09E-15	26.05262
ATP6V0D1	0.535596	9.83224	8.436215	2.57E-16	5.36E-15	26.00068
FAM134B	-0.53257	5.682484	-8.42335	2.83E-16	5.90E-15	25.90413
HPCAL1	0.559047	7.631541	8.414735	3.03E-16	6.29E-15	25.8395
WDR78	-0.53634	4.972174	-8.41449	3.03E-16	6.29E-15	25.83766
TRANK1	0.67075	6.363373	8.410091	3.14E-16	6.49E-15	25.80469
SERPING1	0.717272	10.08071	8.394331	3.53E-16	7.30E-15	25.68669
CRELD1	-0.50206	6.814611	-8.39089	3.63E-16	7.47E-15	25.66093
ESR1	-0.6087	5.46107	-8.3888	3.69E-16	7.57E-15	25.64533
NR1H3	0.592669	7.369547	8.377153	4.03E-16	8.22E-15	25.55827
IQCK	-0.51062	6.418108	-8.36916	4.28E-16	8.69E-15	25.49856
RBMS1	0.558116	9.497623	8.368488	4.30E-16	8.72E-15	25.49357

CCDC121	-0.63498	4.94505	-8.36223	4.51E-16	9.13E-15	25.44687
CD1B	0.606864	4.141226	8.35671	4.70E-16	9.50E-15	25.4057
LRBA	-0.54353	8.929102	-8.35556	4.74E-16	9.57E-15	25.39712
TM6SF1	0.545358	6.716347	8.340425	5.32E-16	1.07E-14	25.28437
ELF4	0.623446	7.084041	8.337459	5.44E-16	1.09E-14	25.2623
ME1	0.617219	6.237818	8.335859	5.50E-16	1.11E-14	25.2504
TAPBPL	0.501353	7.632666	8.329033	5.79E-16	1.16E-14	25.19962
C3	0.856269	11.45328	8.31787	6.30E-16	1.26E-14	25.11665
HLA-DOA	0.500427	5.643125	8.315397	6.42E-16	1.28E-14	25.09828
CELSR2	-0.66561	7.743583	-8.31035	6.67E-16	1.33E-14	25.06083
IL1B	0.623429	6.385828	8.302065	7.10E-16	1.41E-14	24.99933
EVI2A	0.921629	7.87693	8.280928	8.33E-16	1.65E-14	24.84271
LILRB5	0.613553	4.870514	8.277383	8.55E-16	1.69E-14	24.81647
TBC1D9	-0.89945	9.211928	-8.25542	1.01E-15	1.98E-14	24.65413
SAMD9	0.721803	7.026845	8.255203	1.01E-15	1.98E-14	24.65251
VNN2	0.805751	4.844785	8.254422	1.02E-15	1.99E-14	24.64675
SREBF1	-0.6274	7.747455	-8.24541	1.09E-15	2.13E-14	24.58021
ASCC3	0.505526	7.454452	8.238519	1.14E-15	2.23E-14	24.52941
PPP1R9A	-0.56856	6.410426	-8.22072	1.31E-15	2.54E-14	24.39827
CCL4	1.085261	7.374806	8.220333	1.31E-15	2.55E-14	24.39545
S100A9	1.454122	8.35662	8.219621	1.32E-15	2.56E-14	24.39021
NSUN7	-0.53887	4.766377	-8.20321	1.49E-15	2.88E-14	24.26952
SLC1A2	-0.71482	4.95263	-8.20309	1.49E-15	2.88E-14	24.26869
RIPPLY3	-0.66823	5.091476	-8.19915	1.54E-15	2.96E-14	24.23971
IER3	-0.69313	11.14124	-8.19593	1.57E-15	3.02E-14	24.2161
SERPINF1	0.666009	10.65813	8.194932	1.59E-15	3.04E-14	24.20874
RHOF	0.58105	6.195498	8.194207	1.59E-15	3.05E-14	24.20342
LRRC6	-0.70148	6.422854	-8.17904	1.78E-15	3.39E-14	24.09217
DDX58	0.561017	7.673387	8.176366	1.82E-15	3.45E-14	24.07256
DUSP2	0.696487	6.222316	8.172783	1.87E-15	3.54E-14	24.0463
SLC4A8	-0.8033	5.679002	-8.17014	1.91E-15	3.60E-14	24.02693
MRPS30	-0.70834	8.473792	-8.1545	2.14E-15	4.04E-14	23.91249
MAL	0.531059	5.43716	8.126174	2.64E-15	4.95E-14	23.70563
LILRA3	0.527216	4.212113	8.115322	2.86E-15	5.36E-14	23.62654
NOTCH2NL	-0.591	11.44167	-8.09933	3.22E-15	6.01E-14	23.51011
GFRA1	-1.30484	6.687957	-8.09751	3.27E-15	6.08E-14	23.49691
CHST7	0.610204	5.478688	8.091212	3.42E-15	6.35E-14	23.45111
CA12	-1.07314	8.584319	-8.07738	3.79E-15	6.98E-14	23.35068
TNFSF8	0.542478	5.547281	8.064183	4.18E-15	7.66E-14	23.25494
DYNC2H1	-0.55606	5.837378	-8.06125	4.27E-15	7.81E-14	23.23369
RNASE2	0.632981	5.262287	8.054429	4.49E-15	8.19E-14	23.18427
ACP5	0.690985	8.140405	8.048731	4.68E-15	8.53E-14	23.14303
HERC5	0.831589	7.418406	8.04277	4.89E-15	8.90E-14	23.0999
MYO6	-0.64015	8.939239	-8.02569	5.54E-15	1.01E-13	22.97647

GLRB	-0.76169	5.321444	-8.02158	5.71E-15	1.03E-13	22.9468
IGSF6	0.59817	6.573826	8.015636	5.97E-15	1.08E-13	22.90391
MX1	0.986697	9.684253	8.00224	6.58E-15	1.19E-13	22.80735
NRBF2	0.567836	7.405778	7.995404	6.92E-15	1.25E-13	22.75813
PAF1	-0.60106	8.049607	-7.9877	7.32E-15	1.32E-13	22.70267
KCNA3	0.602365	4.602734	7.987555	7.33E-15	1.32E-13	22.70164
CLUL1	-0.5984	5.268554	-7.98706	7.36E-15	1.32E-13	22.69809
KIR2DS5	0.583723	4.387889	7.980221	7.74E-15	1.38E-13	22.64891
C14orf79	-0.61271	6.50893	-7.97945	7.78E-15	1.39E-13	22.64335
ATP8B4	0.623526	4.85657	7.979177	7.79E-15	1.39E-13	22.64141
RPGR	-0.62428	6.151939	-7.9679	8.46E-15	1.51E-13	22.56038
REPS2	-0.747	6.535091	-7.96603	8.58E-15	1.52E-13	22.54698
MAGI2	-0.58818	5.244665	-7.96458	8.67E-15	1.54E-13	22.53658
DDX60	0.785799	8.230809	7.954596	9.33E-15	1.65E-13	22.46497
CCL20	0.929902	4.543353	7.952689	9.46E-15	1.67E-13	22.4513
NME3	-0.85168	8.931653	-7.94014	1.04E-14	1.83E-13	22.36142
ZFYVE16	-0.55713	6.776244	-7.93954	1.04E-14	1.83E-13	22.35712
ENO2	-0.72663	6.945098	-7.93371	1.09E-14	1.91E-13	22.31539
DOCK2	0.646921	6.188774	7.925877	1.15E-14	2.01E-13	22.25938
GPX7	0.635904	7.665777	7.924077	1.16E-14	2.03E-13	22.24652
RBM39	-0.5227	9.722751	-7.91941	1.20E-14	2.10E-13	22.21319
LUC7L3	-0.58829	8.893184	-7.90898	1.30E-14	2.25E-13	22.13867
CHST11	0.868408	7.146263	7.902199	1.36E-14	2.36E-13	22.09033
TBX3	-0.81082	6.730377	-7.89968	1.39E-14	2.40E-13	22.07238
UCN	-0.89304	5.463488	-7.88581	1.54E-14	2.65E-13	21.97355
FMO1	0.863123	7.157991	7.865903	1.78E-14	3.05E-13	21.83199
KIF16B	-0.5221	7.408785	-7.85068	1.98E-14	3.40E-13	21.72394
HMOX1	0.620349	8.143358	7.846929	2.04E-14	3.49E-13	21.69732
NOD2	0.567671	6.142136	7.845368	2.06E-14	3.52E-13	21.68625
SYCP2	-0.74864	5.437829	-7.8445	2.07E-14	3.54E-13	21.68011
CCDC170	-0.67453	5.6483	-7.84349	2.09E-14	3.56E-13	21.67291
ALDH1A1	0.903745	7.156802	7.813219	2.59E-14	4.38E-13	21.45871
WDR60	-0.51138	5.802135	-7.80524	2.75E-14	4.61E-13	21.40232
COL4A5	-0.80849	6.788243	-7.79584	2.94E-14	4.92E-13	21.336
SLC43A3	0.77388	7.968901	7.783638	3.21E-14	5.36E-13	21.25002
LCMT1	-0.50329	9.247036	-7.7749	3.42E-14	5.69E-13	21.18846
CD200	0.517901	6.893425	7.767675	3.60E-14	5.98E-13	21.13766
PBLD	-0.5005	5.916819	-7.76242	3.73E-14	6.20E-13	21.10071
ATP2C2	-0.54333	6.26424	-7.75056	4.07E-14	6.71E-13	21.01741
PRNP	0.557707	9.024242	7.749448	4.10E-14	6.75E-13	21.0096
KRT18	-0.70971	11.90498	-7.74408	4.26E-14	7.00E-13	20.97196
S1PR1	0.536903	6.646851	7.741879	4.33E-14	7.10E-13	20.95649
DNALI1	-0.99055	6.597496	-7.73761	4.46E-14	7.31E-13	20.92658
ENTPD1	0.647815	7.373242	7.734219	4.57E-14	7.48E-13	20.9028

CD4	0.552301	5.503996	7.722294	4.97E-14	8.13E-13	20.81928
SPN	0.518569	4.738928	7.707072	5.54E-14	9.04E-13	20.71282
LY6E	0.703746	9.416323	7.699593	5.84E-14	9.49E-13	20.66059
KCNJ3	-0.96757	4.417163	-7.69717	5.95E-14	9.63E-13	20.64365
PAFAH1B3	-0.51832	9.152935	-7.6956	6.01E-14	9.72E-13	20.63273
GPNMB	0.678736	10.4162	7.694471	6.06E-14	9.79E-13	20.62483
PFN1	0.589777	11.01893	7.693582	6.10E-14	9.84E-13	20.61863
ATP6V0E2	-0.53486	8.984904	-7.69061	6.23E-14	1.00E-12	20.59788
TNFRSF21	0.67316	7.49447	7.689979	6.26E-14	1.01E-12	20.59349
IGLL1	0.552537	5.326669	7.688436	6.33E-14	1.02E-12	20.58273
UCP2	0.564505	9.460369	7.677588	6.83E-14	1.10E-12	20.50712
PNP	0.627253	8.210106	7.666365	7.40E-14	1.19E-12	20.42898
PSAT1	0.935346	6.38702	7.665127	7.46E-14	1.19E-12	20.42038
DOCK10	0.72448	5.902218	7.656008	7.96E-14	1.27E-12	20.35697
GRAP2	0.557368	4.612652	7.655775	7.97E-14	1.27E-12	20.35535
KYNU	0.750753	6.124009	7.638292	9.02E-14	1.44E-12	20.23395
CD7	0.701393	5.328721	7.633295	9.35E-14	1.49E-12	20.1993
RENBP	0.545348	4.878682	7.623055	1.00E-13	1.59E-12	20.12834
IQCH	-0.51133	3.945333	-7.61897	1.03E-13	1.63E-12	20.10004
IFIH1	0.568703	5.791478	7.616676	1.05E-13	1.65E-12	20.08418
KLRF1	0.689853	4.279611	7.612503	1.08E-13	1.70E-12	20.05531
BCAS1	-0.81904	6.162268	-7.6088	1.11E-13	1.74E-12	20.02969
IFT88	-0.73589	7.928524	-7.59878	1.19E-13	1.86E-12	19.96043
FRY	-0.55047	6.001698	-7.598	1.20E-13	1.87E-12	19.95508
UBE2E3	0.840842	9.610461	7.589274	1.27E-13	1.98E-12	19.89482
HPN	-0.59906	7.121593	-7.57322	1.43E-13	2.21E-12	19.78413
MACROD1	-0.52973	6.700135	-7.57281	1.43E-13	2.21E-12	19.78128
EPS8L1	-0.53	6.978312	-7.5592	1.57E-13	2.43E-12	19.68766
TFF1	-1.94347	9.039274	-7.55266	1.65E-13	2.53E-12	19.6427
MLPH	-1.22001	10.35431	-7.54967	1.68E-13	2.58E-12	19.62209
E2F4	0.639695	6.845433	7.549446	1.68E-13	2.58E-12	19.62059
SPI1	0.854911	5.280446	7.545324	1.73E-13	2.65E-12	19.59227
CERS4	-0.75291	7.017411	-7.54509	1.74E-13	2.65E-12	19.59066
GRIA2	-1.22533	4.037961	-7.54175	1.78E-13	2.71E-12	19.56771
MDFIC	0.521601	7.062985	7.53818	1.82E-13	2.78E-12	19.54323
BMPR1B	-1.3348	6.099847	-7.50967	2.22E-13	3.38E-12	19.34791
KCTD3	-0.71629	8.762185	-7.50104	2.36E-13	3.58E-12	19.28887
NFIL3	0.62066	8.050275	7.500006	2.38E-13	3.60E-12	19.28182
RAB11FIP3	-0.50076	6.860391	-7.49858	2.40E-13	3.63E-12	19.27209
GREB1	-0.75185	5.236742	-7.4984	2.41E-13	3.63E-12	19.27086
DHX38	0.598001	6.460043	7.497302	2.42E-13	3.64E-12	19.26334
PGR	-1.16113	5.63403	-7.49511	2.46E-13	3.70E-12	19.24837
RNF2	-0.55734	6.337205	-7.48616	2.62E-13	3.93E-12	19.18725
PPM1H	-0.5694	6.483339	-7.48366	2.67E-13	3.99E-12	19.17024

GM2A	0.58898	7.021691	7.477354	2.78E-13	4.16E-12	19.12722
IQCA1	-0.60235	4.787903	-7.47167	2.90E-13	4.31E-12	19.08848
FAM110B	-0.59513	6.360518	-7.46705	2.99E-13	4.45E-12	19.05704
S100A8	1.35971	6.753451	7.459515	3.15E-13	4.68E-12	19.00574
HLA-DPB1	1.015142	9.999116	7.441449	3.57E-13	5.28E-12	18.88296
SPATA6	-0.54636	5.645951	-7.4406	3.59E-13	5.31E-12	18.87722
PECAM1	0.619978	8.388427	7.439154	3.63E-13	5.35E-12	18.86738
GAMT	-0.53425	7.080521	-7.4384	3.65E-13	5.38E-12	18.86225
DHRS9	0.539003	5.450887	7.423989	4.03E-13	5.91E-12	18.76453
TMEM243	0.580792	8.574119	7.407656	4.51E-13	6.58E-12	18.65395
ANXA9	-0.87029	6.439234	-7.40679	4.54E-13	6.61E-12	18.64811
KCNE4	-0.95694	6.499794	-7.39791	4.82E-13	7.02E-12	18.5881
CCDC106	-0.53439	5.92239	-7.38671	5.21E-13	7.55E-12	18.51246
CSAD	-0.59163	6.811607	-7.38086	5.42E-13	7.84E-12	18.47295
LY86	0.769826	7.992506	7.366052	6.00E-13	8.65E-12	18.3732
CNIH3	-0.54128	5.217838	-7.35164	6.63E-13	9.52E-12	18.27626
IL20RA	-0.70044	5.522175	-7.35092	6.66E-13	9.56E-12	18.27142
SYT17	-0.86755	7.331674	-7.34807	6.79E-13	9.73E-12	18.25225
IFITM1	0.557871	11.80669	7.332262	7.57E-13	1.08E-11	18.14614
NOVA1	-0.74905	4.857875	-7.32296	8.06E-13	1.15E-11	18.08376
PLAT	-0.85625	8.932192	-7.31807	8.34E-13	1.18E-11	18.05106
KAT6B	-0.52104	7.704202	-7.31197	8.69E-13	1.23E-11	18.01019
SPDEF	-0.57328	8.209574	-7.31165	8.71E-13	1.23E-11	18.00808
GPRC5A	-0.8213	8.067916	-7.31165	8.71E-13	1.23E-11	18.00808
PLEKHA5	-0.58821	7.027614	-7.3097	8.83E-13	1.25E-11	17.99504
AP1M2	-0.60395	8.0269	-7.30176	9.32E-13	1.31E-11	17.94196
GRP	-0.96189	6.910225	-7.30174	9.32E-13	1.31E-11	17.94178
FOXA1	-1.24598	8.130778	-7.27582	1.11E-12	1.56E-11	17.76882
BSPRY	-0.632	8.363437	-7.26979	1.16E-12	1.63E-11	17.72864
CACNA2D2	-0.64761	6.530739	-7.26912	1.16E-12	1.63E-11	17.7242
ITM2A	0.800861	8.34982	7.261623	1.22E-12	1.72E-11	17.67428
SPATA20	-0.53343	8.427507	-7.25739	1.26E-12	1.76E-11	17.64613
APBB1IP	0.525631	5.948655	7.249971	1.32E-12	1.84E-11	17.59682
GALR3	0.722632	5.546484	7.24758	1.35E-12	1.87E-11	17.58094
NPY1R	-1.52066	6.910767	-7.2467	1.35E-12	1.88E-11	17.57506
TRIM9	-0.56783	4.50141	-7.24034	1.41E-12	1.96E-11	17.53288
CASC1	-0.69693	4.202543	-7.23739	1.44E-12	1.99E-11	17.51331
INPP4B	-0.83505	6.753673	-7.23541	1.46E-12	2.02E-11	17.50014
IFIT1	0.961487	9.099821	7.234462	1.47E-12	2.03E-11	17.49387
CD82	0.546609	7.894499	7.229783	1.52E-12	2.09E-11	17.46284
WDR19	-0.61057	6.197034	-7.2138	1.69E-12	2.32E-11	17.35702
WWP1	-0.59046	9.572824	-7.20824	1.76E-12	2.40E-11	17.32024
ANKEF1	-0.60017	5.637827	-7.20786	1.76E-12	2.41E-11	17.31774
KIF3A	-0.56502	7.063907	-7.20505	1.79E-12	2.45E-11	17.29912

ADAM15	0.768903	6.804429	7.202366	1.83E-12	2.49E-11	17.28139
F13A1	0.800935	8.422962	7.200749	1.85E-12	2.51E-11	17.27072
ASS1	0.80208	9.497055	7.193568	1.94E-12	2.62E-11	17.22329
DLX2	-0.56136	5.138417	-7.19252	1.95E-12	2.64E-11	17.21635
AGR2	-1.59369	9.43119	-7.17762	2.16E-12	2.91E-11	17.11812
TUFT1	-0.56268	8.992239	-7.17056	2.26E-12	3.04E-11	17.0716
MLLT4-AS1	-0.58747	5.109676	-7.17049	2.26E-12	3.04E-11	17.07119
NKAIN1	-0.75415	6.095097	-7.16974	2.28E-12	3.06E-11	17.06623
CYBA	0.588176	8.529424	7.155308	2.51E-12	3.36E-11	16.97131
CXCL12	0.681226	9.340667	7.148605	2.62E-12	3.50E-11	16.92728
CEP290	-0.89184	6.234633	-7.14645	2.66E-12	3.55E-11	16.91311
C3orf52	-0.59905	6.029602	-7.14392	2.71E-12	3.61E-11	16.89652
PTGIR	0.542417	5.720321	7.138426	2.81E-12	3.74E-11	16.86048
GPR132	0.512038	4.213116	7.130181	2.97E-12	3.94E-11	16.80644
ALOX5	0.543233	6.534555	7.12586	3.05E-12	4.05E-11	16.77813
CHIC2	0.666517	8.016657	7.109548	3.40E-12	4.50E-11	16.67141
LAT	0.549961	6.649516	7.10847	3.43E-12	4.53E-11	16.66437
CCL22	0.553106	6.012783	7.09834	3.67E-12	4.84E-11	16.5982
XYLT2	-0.5788	5.808582	-7.08846	3.92E-12	5.16E-11	16.53373
GREB1L	-0.72931	5.755823	-7.08532	4.00E-12	5.25E-11	16.51328
IL7	0.538573	4.501838	7.08261	4.07E-12	5.34E-11	16.49563
SH3BP1	0.583186	5.634444	7.079308	4.16E-12	5.44E-11	16.47412
ACSL1	0.550208	8.737784	7.057092	4.83E-12	6.26E-11	16.32962
HLA-DQB1	0.739889	7.130697	7.051459	5.01E-12	6.49E-11	16.29304
MEF2C	0.52231	7.701813	7.050211	5.05E-12	6.54E-11	16.28495
IGF1R	-0.65835	7.49375	-7.04382	5.27E-12	6.82E-11	16.24349
EPB41L5	-0.50208	6.372901	-7.04045	5.39E-12	6.96E-11	16.22165
PAN2	-0.61178	7.343652	-7.03121	5.73E-12	7.39E-11	16.16176
SCUBE2	-1.374	8.748075	-7.02856	5.83E-12	7.51E-11	16.14462
GLS2	-0.5215	4.474307	-7.02796	5.85E-12	7.53E-11	16.14076
KIF13B	-0.51765	7.819739	-7.01947	6.19E-12	7.95E-11	16.0858
DNAJC22	-0.59592	5.937933	-7.01897	6.21E-12	7.96E-11	16.08254
FAM173A	-0.55532	8.126171	-7.01818	6.24E-12	8.00E-11	16.07748
LILRB3	0.555645	7.01224	6.997918	7.13E-12	9.08E-11	15.94662
KMO	0.753889	6.429842	6.976406	8.22E-12	1.04E-10	15.80806
MEGF9	-0.52581	7.42122	-6.97573	8.25E-12	1.04E-10	15.80375
CYP1B1	0.634497	8.108801	6.970781	8.53E-12	1.08E-10	15.77189
NME5	-0.90907	6.422475	-6.96953	8.60E-12	1.08E-10	15.76384
SELP	0.53396	6.592037	6.966224	8.79E-12	1.11E-10	15.7426
PLK1	0.876549	5.932608	6.957546	9.30E-12	1.17E-10	15.68688
ARID3B	0.566424	5.537937	6.947506	9.93E-12	1.24E-10	15.62248
TJP3	-0.62159	7.118142	-6.92188	1.17E-11	1.46E-10	15.45845
CRLF3	0.523249	8.684561	6.91918	1.20E-11	1.49E-10	15.44122
APOBR	0.561812	5.246877	6.914101	1.24E-11	1.54E-10	15.40879

SPDYE2	-0.5565	6.844103	-6.90771	1.29E-11	1.60E-10	15.36801
EEF1A2	-0.86742	7.020195	-6.90722	1.29E-11	1.60E-10	15.36485
CAP2	-0.60974	8.298148	-6.90622	1.30E-11	1.61E-10	15.35848
TRPS1	-0.61966	10.42191	-6.89433	1.41E-11	1.73E-10	15.28275
STIP1	0.611943	8.029883	6.890237	1.44E-11	1.77E-10	15.25667
POU2F3	-0.60595	4.565421	-6.8886	1.46E-11	1.79E-10	15.24624
SLC5A2	0.576544	5.237496	6.883058	1.51E-11	1.85E-10	15.21099
LGALS9	0.590424	7.673239	6.872926	1.61E-11	1.97E-10	15.1466
PPIF	0.568836	8.367806	6.865028	1.70E-11	2.07E-10	15.09646
HIST2H2BE	-0.69943	8.51546	-6.86397	1.71E-11	2.08E-10	15.08973
CD300C	0.546833	5.292487	6.861116	1.74E-11	2.11E-10	15.07164
GSTP1	0.705941	9.808945	6.856679	1.79E-11	2.17E-10	15.0435
CIRBP	-0.52268	9.502559	-6.84838	1.89E-11	2.28E-10	14.99095
RAB17	-0.55204	7.052644	-6.84414	1.95E-11	2.35E-10	14.96408
CD83	0.578545	8.088324	6.841865	1.97E-11	2.38E-10	14.94969
TBX19	0.58102	5.510966	6.832308	2.10E-11	2.51E-10	14.88926
FXYD3	-0.56402	9.85804	-6.81989	2.28E-11	2.71E-10	14.81081
FAM46C	0.628114	7.212951	6.804188	2.52E-11	2.99E-10	14.71186
CDC45	0.690169	5.565476	6.801877	2.56E-11	3.03E-10	14.6973
SORD	-0.57078	8.032957	-6.79172	2.73E-11	3.22E-10	14.63341
PLBD1	0.573369	8.917634	6.789018	2.78E-11	3.27E-10	14.61641
HES1	-0.52826	7.511615	-6.78482	2.85E-11	3.36E-10	14.59005
SELE	0.665041	5.406289	6.776933	3.00E-11	3.53E-10	14.54051
LZTFL1	-0.58208	6.562465	-6.77657	3.01E-11	3.53E-10	14.53821
FMO3	0.65951	4.939158	6.771168	3.11E-11	3.65E-10	14.50434
CBR1	0.594137	7.343301	6.765351	3.23E-11	3.78E-10	14.46787
MZT2B	-0.70182	8.290073	-6.76253	3.29E-11	3.84E-10	14.45018
MMP12	1.279636	6.152958	6.748916	3.59E-11	4.17E-10	14.36499
KEL	0.528149	4.830056	6.73623	3.89E-11	4.50E-10	14.28571
ASCL1	-0.51921	4.331878	-6.73153	4.01E-11	4.63E-10	14.25635
PBX1	-0.51425	9.823045	-6.72949	4.06E-11	4.69E-10	14.24366
LDHB	0.689837	11.36167	6.726455	4.14E-11	4.77E-10	14.22472
HOOK1	-0.68587	7.082697	-6.72256	4.25E-11	4.88E-10	14.20042
CFI	0.563919	6.697277	6.720345	4.31E-11	4.93E-10	14.18664
MAGI1	-0.54118	5.997584	-6.71374	4.49E-11	5.14E-10	14.14551
CAV3	0.609834	5.191344	6.712738	4.52E-11	5.17E-10	14.13925
C1QTNF3	-0.60144	6.684159	-6.68866	5.27E-11	5.96E-10	13.9896
YTHDC2	-0.50279	6.708732	-6.67301	5.82E-11	6.54E-10	13.89259
SLC16A6	-0.75624	7.210531	-6.66802	6.01E-11	6.73E-10	13.86166
OAS3	0.664029	7.388687	6.660106	6.31E-11	7.07E-10	13.81272
GDF15	-0.5165	5.132903	-6.65502	6.52E-11	7.28E-10	13.7813
ZNF552	-0.53066	6.813269	-6.65342	6.59E-11	7.35E-10	13.77137
COASY	-0.51116	8.618348	-6.64852	6.79E-11	7.56E-10	13.74116
ARL2BP	0.512084	8.41265	6.638939	7.22E-11	8.00E-10	13.68202

SELENBP1	-0.59391	9.171905	-6.63609	7.35E-11	8.14E-10	13.66446
OLFML1	0.635449	6.930069	6.634928	7.40E-11	8.19E-10	13.65729
GPX3	0.645244	8.707543	6.626298	7.82E-11	8.61E-10	13.60413
PP14571	-0.83272	5.27656	-6.62607	7.83E-11	8.62E-10	13.60273
FBLN5	0.664756	8.115462	6.625817	7.84E-11	8.62E-10	13.60117
GVINP1	0.551903	5.291874	6.610087	8.65E-11	9.48E-10	13.50444
TMEM106C	-0.52447	8.566508	-6.58072	1.04E-10	1.13E-09	13.3244
TMA16	-0.58965	6.191781	-6.57146	1.10E-10	1.19E-09	13.26773
PLA2G2A	0.787512	7.056723	6.564454	1.15E-10	1.24E-09	13.22494
CSGALNACT1	0.569518	7.483483	6.560388	1.18E-10	1.27E-09	13.20012
CD53	0.892595	8.012975	6.559807	1.19E-10	1.28E-09	13.19657
GCH1	0.725999	8.324871	6.544006	1.31E-10	1.40E-09	13.10024
FABP5	0.778141	9.381477	6.539633	1.35E-10	1.44E-09	13.07361
ANKS1B	-0.58942	4.344597	-6.53311	1.40E-10	1.50E-09	13.03395
DPEP1	0.543549	5.338106	6.526628	1.46E-10	1.55E-09	12.99452
BBS4	-0.58637	7.390756	-6.52439	1.48E-10	1.57E-09	12.98095
SYT2	0.554163	4.921316	6.516488	1.55E-10	1.65E-09	12.93295
ZNF562	-0.53435	6.895565	-6.4935	1.79E-10	1.89E-09	12.79368
SCNN1A	-0.50949	6.901653	-6.48839	1.85E-10	1.94E-09	12.76274
NR0B2	0.523833	4.842912	6.472818	2.04E-10	2.13E-09	12.66872
KIAA1324	-0.8929	8.573663	-6.47068	2.06E-10	2.16E-09	12.65584
BEX1	-1.16623	6.070419	-6.46974	2.08E-10	2.17E-09	12.65018
BCAS4	-0.50714	6.460557	-6.46362	2.15E-10	2.25E-09	12.61328
MYRIP	-0.54582	4.687121	-6.44672	2.39E-10	2.49E-09	12.51156
TGFBR2	0.606711	7.677086	6.436791	2.54E-10	2.63E-09	12.4519
AHSA2	-0.54833	7.09055	-6.43268	2.61E-10	2.69E-09	12.42723
ATRNL1	-0.50144	4.207426	-6.43049	2.64E-10	2.72E-09	12.41409
UGDH	-0.58975	9.83304	-6.42724	2.70E-10	2.77E-09	12.39458
ALDH8A1	-0.67935	4.638781	-6.40625	3.07E-10	3.13E-09	12.26892
MPPED2	-0.62951	5.055446	-6.40034	3.18E-10	3.24E-09	12.23358
HSPA2	-0.73007	8.256636	-6.39147	3.36E-10	3.40E-09	12.18061
PDE1B	0.541325	4.809859	6.372045	3.78E-10	3.81E-09	12.06487
CENPA	0.511403	6.426169	6.369578	3.84E-10	3.87E-09	12.05019
LIPG	0.568218	4.856156	6.367765	3.88E-10	3.91E-09	12.03941
HOXC8	-0.61258	5.750634	-6.36574	3.93E-10	3.95E-09	12.02738
SPINK2	0.540913	4.353528	6.364809	3.95E-10	3.97E-09	12.02183
IMPDH2	-0.52415	10.98472	-6.36235	4.01E-10	4.03E-09	12.00719
KIAA1614	0.535869	4.800731	6.340539	4.58E-10	4.57E-09	11.87777
RRAGD	0.521151	7.36211	6.336866	4.68E-10	4.66E-09	11.85601
CTSK	0.629721	11.00417	6.334635	4.75E-10	4.72E-09	11.8428
SALL2	-0.5601	7.341555	-6.32458	5.04E-10	4.99E-09	11.78328
PDCD1	0.506083	5.660213	6.319492	5.20E-10	5.14E-09	11.75324
TDO2	0.697495	5.328405	6.314625	5.36E-10	5.29E-09	11.72448
STX11	0.679097	4.565412	6.30933	5.53E-10	5.45E-09	11.69323

FLRT3	-0.82571	6.147809	-6.30424	5.70E-10	5.61E-09	11.6632
AFF3	-0.81551	4.933381	-6.30232	5.77E-10	5.67E-09	11.65191
CLIC4	0.549995	8.607894	6.27828	6.67E-10	6.52E-09	11.51042
SEC14L2	-0.60058	6.08801	-6.26498	7.23E-10	7.04E-09	11.43237
TSPAN7	0.663283	6.552023	6.264571	7.25E-10	7.05E-09	11.42996
PPP1R3C	-0.83182	6.81865	-6.25004	7.91E-10	7.62E-09	11.34485
EML4	0.517394	7.043505	6.25004	7.91E-10	7.62E-09	11.34484
CTNND2	-0.54394	5.007385	-6.24242	8.28E-10	7.94E-09	11.30027
SERPINA5	-0.91347	6.76251	-6.23869	8.46E-10	8.11E-09	11.27845
TPBG	-0.65965	9.25765	-6.23837	8.48E-10	8.12E-09	11.27663
PLK2	-0.70483	9.1861	-6.22374	9.26E-10	8.83E-09	11.19121
CHODL	0.652343	4.735356	6.215216	9.74E-10	9.27E-09	11.14155
LPXN	0.506706	7.663138	6.214376	9.79E-10	9.31E-09	11.13666
GSTM3	-0.90545	8.933573	-6.20494	1.04E-09	9.81E-09	11.08177
PTPRT	-0.78022	5.920532	-6.19613	1.09E-09	1.03E-08	11.03057
SLC27A2	-0.77242	5.960176	-6.19597	1.09E-09	1.03E-08	11.02961
ITGAX	0.522861	5.657167	6.19432	1.10E-09	1.04E-08	11.02005
LINC00472	-0.73509	5.285789	-6.19394	1.11E-09	1.04E-08	11.01784
ARHGEF26	-0.55327	5.512523	-6.19051	1.13E-09	1.06E-08	10.9979
NCAPH	0.658168	5.617909	6.18997	1.13E-09	1.06E-08	10.9948
MMP7	0.948441	8.487309	6.170769	1.27E-09	1.18E-08	10.88353
RALGPS2	-0.56138	6.374713	-6.16881	1.28E-09	1.19E-08	10.87221
SLC2A6	0.552817	5.911493	6.167852	1.29E-09	1.20E-08	10.86666
CFH	0.620671	7.883996	6.164555	1.32E-09	1.22E-08	10.84759
SLC6A7	0.542754	4.508266	6.16415	1.32E-09	1.22E-08	10.84525
CYP2B7P	-1.02403	6.339642	-6.15857	1.36E-09	1.26E-08	10.81299
ACADSB	-0.79381	6.954945	-6.1549	1.39E-09	1.28E-08	10.79182
YTHDC1	-0.50609	7.047745	-6.14479	1.48E-09	1.36E-08	10.73348
TOX3	-0.88426	7.302694	-6.14107	1.51E-09	1.38E-08	10.71202
AMPD1	0.519317	4.927151	6.133333	1.58E-09	1.44E-08	10.66748
ADD3	0.536657	9.719276	6.130814	1.61E-09	1.46E-08	10.65299
SLC22A5	-0.51214	6.640063	-6.12844	1.63E-09	1.48E-08	10.63931
CITED1	-0.66507	5.665165	-6.11633	1.75E-09	1.59E-08	10.56972
GRAMD1C	-0.65965	6.271798	-6.11016	1.82E-09	1.64E-08	10.53434
PIK3CG	0.607772	5.134923	6.083066	2.13E-09	1.91E-08	10.37919
ENPP1	-0.68764	6.489794	-6.07841	2.19E-09	1.95E-08	10.35257
PDZK1	-1.1174	5.535991	-6.07711	2.21E-09	1.97E-08	10.34516
REEP1	-0.71701	7.090087	-6.07293	2.26E-09	2.01E-08	10.32132
AR	-0.78189	8.029001	-6.06458	2.37E-09	2.11E-08	10.27367
HIST1H3E	-0.52549	5.469911	-6.05454	2.52E-09	2.22E-08	10.21653
PRKAA2	-0.54445	5.447837	-6.05432	2.52E-09	2.22E-08	10.21528
FLT3LG	0.61934	5.293559	6.04302	2.69E-09	2.36E-08	10.15102
NMI	0.656233	9.173052	6.013734	3.19E-09	2.79E-08	9.984989
LRP8	0.520623	6.386336	6.010315	3.26E-09	2.84E-08	9.965653

ERI2	-0.6805	6.277366	-6.00548	3.35E-09	2.92E-08	9.938317
FAM189A2	-0.54801	6.826926	-5.99845	3.49E-09	3.04E-08	9.898629
PRLR	-0.59736	6.583531	-5.9904	3.66E-09	3.17E-08	9.853225
TEX14	-0.57173	4.170827	-5.98938	3.68E-09	3.19E-08	9.847475
ADORA2B	0.626852	5.616594	5.968153	4.16E-09	3.58E-08	9.728006
CPB1	-1.51247	6.71993	-5.95619	4.46E-09	3.82E-08	9.660854
AREG	-0.7968	5.39176	-5.94799	4.67E-09	4.00E-08	9.614872
RET	-0.53174	6.173307	-5.94678	4.70E-09	4.02E-08	9.608104
NR4A2	-0.67571	7.311767	-5.94285	4.81E-09	4.11E-08	9.586101
TSPAN1	-0.84592	7.536096	-5.93637	4.99E-09	4.26E-08	9.549822
PRSS8	-0.52159	8.82921	-5.9267	5.28E-09	4.48E-08	9.495783
DECR2	-0.50662	8.340086	-5.9258	5.31E-09	4.50E-08	9.490787
CD74	1.098383	9.986057	5.919593	5.50E-09	4.66E-08	9.456135
AZGP1	-0.9609	11.16275	-5.91762	5.56E-09	4.70E-08	9.445125
CYBB	0.701865	6.271097	5.91287	5.72E-09	4.82E-08	9.418651
GLCE	-0.53394	7.067168	-5.91264	5.72E-09	4.82E-08	9.417362
MTCL1	-0.5329	7.039337	-5.90139	6.10E-09	5.12E-08	9.354739
C2	0.539218	6.330116	5.894386	6.35E-09	5.31E-08	9.315785
VAV3	-0.61921	8.354401	-5.89127	6.47E-09	5.39E-08	9.298481
SYBU	-0.70955	7.525791	-5.88704	6.63E-09	5.52E-08	9.274987
TSPAN13	-0.62675	11.87235	-5.8836	6.76E-09	5.62E-08	9.255873
STK32B	-0.65805	5.853765	-5.8771	7.01E-09	5.81E-08	9.219867
TMC5	-0.9592	6.862738	-5.86863	7.36E-09	6.08E-08	9.172923
SMA4	-0.67277	7.803931	-5.8521	8.08E-09	6.64E-08	9.081557
AGTR1	-0.8258	6.358693	-5.84531	8.40E-09	6.87E-08	9.044048
SLC34A2	0.75058	5.87127	5.843922	8.47E-09	6.92E-08	9.036401
LRRC17	-0.73382	6.493014	-5.8436	8.48E-09	6.93E-08	9.03462
SYT1	-0.77859	5.284675	-5.84042	8.64E-09	7.05E-08	9.017086
MATN3	-0.80654	5.307735	-5.83684	8.82E-09	7.18E-08	8.997366
CELSR1	-0.5319	5.669987	-5.81991	9.70E-09	7.86E-08	8.904229
GRIK3	-0.55011	4.174688	-5.81362	1.01E-08	8.13E-08	8.869674
PADI3	0.515137	4.39797	5.79918	1.09E-08	8.76E-08	8.790489
STK17B	0.630988	6.426709	5.795048	1.12E-08	8.96E-08	8.767863
MED13L	-0.54563	8.197842	-5.79492	1.12E-08	8.96E-08	8.767174
PROM1	1.130079	8.42349	5.793723	1.12E-08	9.00E-08	8.760608
C5orf42	-0.56347	4.712352	-5.79359	1.13E-08	9.00E-08	8.759906
E2F5	0.533995	6.872447	5.792802	1.13E-08	9.03E-08	8.755569
CLEC2B	0.713097	7.969741	5.788911	1.16E-08	9.21E-08	8.73428
SERPINA3	-0.91528	11.24023	-5.775	1.25E-08	9.89E-08	8.65826
RHCG	0.547666	4.350289	5.774203	1.26E-08	9.92E-08	8.653936
ANP32E	0.652924	8.16277	5.770492	1.28E-08	1.01E-07	8.633697
ANKRD36B	-0.74837	8.759789	-5.76923	1.29E-08	1.02E-07	8.626797
HLA-DQA1	1.449384	7.84189	5.769047	1.29E-08	1.02E-07	8.625816
DSPP	-0.57715	4.882938	-5.75359	1.41E-08	1.10E-07	8.541651

TREM2	0.582512	7.262986	5.750519	1.43E-08	1.12E-07	8.524947
ARHGDIB	0.562637	9.9135	5.749184	1.44E-08	1.13E-07	8.517689
RAPGEF4	-0.61605	5.421129	-5.74399	1.49E-08	1.16E-07	8.489474
FAM171A1	0.535379	7.975861	5.740207	1.52E-08	1.18E-07	8.46893
IFIT2	0.578254	7.452968	5.740167	1.52E-08	1.18E-07	8.468713
EPN3	-0.53371	6.95686	-5.73352	1.58E-08	1.23E-07	8.432633
CROT	-0.63718	6.919093	-5.72588	1.65E-08	1.28E-07	8.391247
CD80	0.523724	5.005119	5.717127	1.73E-08	1.34E-07	8.34388
TMEM212	-0.56921	5.190617	-5.71332	1.76E-08	1.36E-07	8.323308
LBR	0.560815	9.120327	5.711946	1.78E-08	1.37E-07	8.315869
CERS6	-0.51187	8.809715	-5.70677	1.83E-08	1.41E-07	8.287926
CDC42EP1	0.591446	6.543693	5.693319	1.97E-08	1.51E-07	8.215359
PER3	-0.54095	5.98869	-5.68494	2.07E-08	1.58E-07	8.170249
EVL	-0.55765	9.291981	-5.67519	2.18E-08	1.65E-07	8.117843
SNX10	0.580362	8.173105	5.667954	2.27E-08	1.72E-07	8.078962
EFHD1	-0.54975	10.86273	-5.65801	2.40E-08	1.81E-07	8.025643
RNF103	-0.51586	8.186911	-5.64096	2.63E-08	1.98E-07	7.934402
KCNN4	0.554755	6.889098	5.637908	2.68E-08	2.01E-07	7.918103
MYOD1	0.502705	4.669786	5.632061	2.77E-08	2.06E-07	7.886885
SLC1A3	0.523062	6.432123	5.596176	3.37E-08	2.48E-07	7.695947
IL33	0.730383	5.675567	5.573125	3.82E-08	2.79E-07	7.573874
FCGR2A	0.542471	6.597296	5.571543	3.85E-08	2.81E-07	7.565511
TGM2	0.612433	6.137263	5.549397	4.35E-08	3.15E-07	7.448686
ACKR1	0.737791	7.126615	5.547038	4.40E-08	3.19E-07	7.43627
SH3BP4	-0.62062	8.331376	-5.54353	4.49E-08	3.24E-07	7.417833
SLPI	0.845645	8.802569	5.536277	4.67E-08	3.36E-07	7.379675
DPY19L2P2	-0.50987	4.311874	-5.53067	4.81E-08	3.46E-07	7.350214
DHRS2	-1.02544	6.239486	-5.51626	5.20E-08	3.72E-07	7.274657
C1orf21	-0.57151	6.588472	-5.50902	5.41E-08	3.85E-07	7.236761
CDCA8	0.586513	6.665567	5.492077	5.93E-08	4.20E-07	7.148261
HOXC6	-0.70262	9.305785	-5.47751	6.41E-08	4.50E-07	7.072357
MST1	-0.50071	6.566642	-5.47628	6.45E-08	4.53E-07	7.065982
SYT13	-0.54822	5.668631	-5.46904	6.71E-08	4.70E-07	7.028324
EP300	-0.52155	7.814556	-5.46599	6.82E-08	4.76E-07	7.012471
SLC12A5	-0.508	4.162669	-5.44991	7.43E-08	5.18E-07	6.929071
SLC6A4	-0.68428	4.894918	-5.43996	7.84E-08	5.45E-07	6.877553
PRR15L	-0.55811	7.991785	-5.40101	9.65E-08	6.57E-07	6.676738
HBB	0.798604	8.256196	5.400316	9.68E-08	6.59E-07	6.673178
LXN	0.626337	8.516794	5.39177	1.01E-07	6.87E-07	6.6293
GPR1	-0.50349	5.221055	-5.36345	1.18E-07	7.87E-07	6.484335
PRAME	0.793437	5.759882	5.358246	1.21E-07	8.06E-07	6.457789
NBEA	-0.52359	6.690772	-5.34642	1.29E-07	8.54E-07	6.397536
NRIP3	-0.67215	6.984059	-5.33209	1.39E-07	9.17E-07	6.324638
PARD6B	-0.66639	5.339925	-5.2664	1.96E-07	1.26E-06	5.992857

ATP1B1	-0.6068	11.22322	-5.25767	2.05E-07	1.32E-06	5.94905
CCL7	0.553151	4.549381	5.253394	2.09E-07	1.34E-06	5.927624
FKBP5	0.70806	7.13466	5.239796	2.25E-07	1.43E-06	5.859562
PLA2G4A	0.566737	5.852598	5.226013	2.41E-07	1.52E-06	5.790743
GALNT14	0.552207	5.886418	5.211735	2.60E-07	1.63E-06	5.719626
IL12RB2	0.524883	4.597323	5.178471	3.08E-07	1.90E-06	5.554627
ZNF611	-0.60521	7.20135	-5.16032	3.38E-07	2.07E-06	5.465005
ACTL8	0.537514	5.351793	5.160266	3.38E-07	2.07E-06	5.464735
PLEKHA6	-0.52708	6.412325	-5.14071	3.74E-07	2.27E-06	5.368478
TMEM176B	0.564746	7.889482	5.128726	3.97E-07	2.40E-06	5.309675
DPT	0.522275	7.815564	5.1175	4.20E-07	2.53E-06	5.254695
CRIP1	-0.76563	10.28649	-5.11658	4.22E-07	2.54E-06	5.250208
UCHL1	0.552478	6.200967	5.099904	4.60E-07	2.75E-06	5.168741
GALNT6	-0.67252	9.163863	-5.09721	4.66E-07	2.78E-06	5.155603
LAPTM4B	0.53902	10.10842	5.094403	4.73E-07	2.81E-06	5.141924
NBR2	-0.50812	6.082854	-5.08514	4.95E-07	2.94E-06	5.096833
CLEC7A	0.591104	6.025197	5.083618	4.99E-07	2.96E-06	5.089426
ARNT2	-0.55052	7.036176	-5.07676	5.17E-07	3.05E-06	5.056105
MKL2	-0.81498	7.302046	-5.07314	5.26E-07	3.10E-06	5.038504
ERAP2	0.537316	6.030608	5.062172	5.56E-07	3.26E-06	4.985337
PGBD5	0.603433	5.424218	5.05498	5.76E-07	3.38E-06	4.950524
ALCAM	-0.53763	9.102062	-5.04266	6.13E-07	3.57E-06	4.890979
ADIRF	-0.6976	9.354447	-5.01926	6.89E-07	3.97E-06	4.778255
MCCC2	-0.51408	7.534161	-5.014	7.08E-07	4.07E-06	4.753011
OXTR	-0.55211	6.556961	-4.96512	9.02E-07	5.11E-06	4.51932
MUC1	-0.65593	9.323031	-4.95187	9.63E-07	5.43E-06	4.456347
QPCT	0.618503	6.970843	4.937855	1.03E-06	5.75E-06	4.389914
TCEAL1	-0.51057	8.342897	-4.9135	1.16E-06	6.40E-06	4.274863
GALNT7	-0.5465	9.38539	-4.8771	1.39E-06	7.52E-06	4.103895
CLGN	-0.86735	6.151551	-4.8279	1.76E-06	9.32E-06	3.874641
LAMA3	-0.58501	5.62173	-4.82412	1.80E-06	9.47E-06	3.85711
SLC2A10	-0.50698	9.421205	-4.81727	1.86E-06	9.73E-06	3.825366
ATP6V0C	0.533686	9.903233	4.794575	2.07E-06	1.07E-05	3.720575
ST6GAL1	0.513518	6.265182	4.788202	2.13E-06	1.10E-05	3.691225
FXYD1	0.527019	5.975948	4.786423	2.15E-06	1.11E-05	3.683039
LBP	0.67264	5.247215	4.765163	2.38E-06	1.22E-05	3.585429
PIP	-1.1624	10.10035	-4.75243	2.53E-06	1.29E-05	3.527152
HMGCS2	-0.74843	5.573948	-4.74739	2.59E-06	1.31E-05	3.504118
PVALB	-0.77583	4.928333	-4.74644	2.61E-06	1.32E-05	3.499795
PI3	0.578996	4.868344	4.733262	2.77E-06	1.40E-05	3.439711
NDP	-0.66319	5.389872	-4.71041	3.09E-06	1.53E-05	3.335886
SLC2A3	0.556952	7.144457	4.705475	3.16E-06	1.56E-05	3.313531
STEAP1	0.509494	7.874875	4.681692	3.54E-06	1.73E-05	3.206077
SLC44A4	-0.71867	6.733189	-4.6666	3.80E-06	1.85E-05	3.138169

DKK1	0.789601	5.845321	4.643057	4.24E-06	2.05E-05	3.032592
NAT1	-1.04664	9.3357	-4.63665	4.37E-06	2.11E-05	3.003954
HSPB8	-0.50284	6.950239	-4.60914	4.97E-06	2.37E-05	2.881412
ACYP1	-0.50428	7.432462	-4.59982	5.19E-06	2.46E-05	2.840026
NF1	-0.50326	4.16304	-4.58523	5.55E-06	2.61E-05	2.775431
PDK1	0.502309	6.084478	4.582947	5.61E-06	2.64E-05	2.765343
SLC7A2	-0.60769	5.397817	-4.57124	5.92E-06	2.77E-05	2.713675
ACOX2	-0.59319	7.252951	-4.56448	6.11E-06	2.85E-05	2.683904
KLK6	0.663135	5.778328	4.557147	6.31E-06	2.94E-05	2.651628
MORF4L2	-0.65603	9.987908	-4.52743	7.24E-06	3.33E-05	2.521393
DEPTOR	-0.52265	7.373494	-4.52188	7.42E-06	3.40E-05	2.497175
HTR2B	0.530154	5.656085	4.510844	7.80E-06	3.55E-05	2.449047
MMP10	-0.5565	5.108256	-4.498	8.27E-06	3.75E-05	2.393197
GREM1	0.512664	7.927461	4.49059	8.56E-06	3.86E-05	2.361041
MFAP4	0.723406	6.856066	4.478662	9.03E-06	4.06E-05	2.309382
NABP1	0.61067	6.056002	4.469948	9.40E-06	4.21E-05	2.271724
ANKRD36	-0.72029	6.425487	-4.4693	9.42E-06	4.22E-05	2.268919
C1orf106	0.551366	7.356544	4.44861	1.03E-05	4.60E-05	2.179807
PTHLH	-0.55719	5.204841	-4.44641	1.04E-05	4.64E-05	2.170358
HSD17B2	0.636494	4.840338	4.434195	1.10E-05	4.89E-05	2.117939
CAMLG	-0.50052	9.862967	-4.4223	1.16E-05	5.13E-05	2.06705
KCNK15	-0.50993	4.908147	-4.35679	1.56E-05	6.70E-05	1.788949
WISP2	0.564829	7.135452	4.35653	1.56E-05	6.70E-05	1.787859
HSD17B4	-0.50927	8.053039	-4.3351	1.72E-05	7.29E-05	1.697756
CYP4B1	-0.65763	6.275682	-4.33484	1.72E-05	7.30E-05	1.69664
MMP3	0.669492	7.771158	4.330918	1.75E-05	7.42E-05	1.680206
UGT2B4	-0.55538	2.869604	-4.30674	1.94E-05	8.19E-05	1.579146
RNF128	-0.57364	6.18218	-4.30012	2.00E-05	8.41E-05	1.55156
LRIG1	-0.60417	7.029012	-4.29198	2.07E-05	8.67E-05	1.517695
COX6C	-0.56584	12.51407	-4.28795	2.11E-05	8.80E-05	1.500921
LCN2	0.6199	6.230564	4.285233	2.13E-05	8.90E-05	1.489658
CHI3L1	0.595135	7.258556	4.262952	2.35E-05	9.71E-05	1.397416
APOBEC3B	0.519462	7.485678	4.259666	2.39E-05	9.83E-05	1.383848
C1orf54	0.535941	8.710455	4.208896	2.97E-05	0.00012	1.175502
FOXM1	0.521601	6.603657	4.207822	2.98E-05	0.00012	1.17112
CGA	-0.53832	4.304083	-4.19235	3.19E-05	0.000128	1.10809
ESRRG	-0.51864	5.950861	-4.19067	3.21E-05	0.000129	1.101283
LTF	0.896988	9.738503	4.172233	3.47E-05	0.000138	1.026508
RPL27A	-0.50872	8.736489	-4.12578	4.23E-05	0.000165	0.839473
CAMK2N1	-0.53175	8.430173	-4.12555	4.23E-05	0.000165	0.838562
FCGBP	-0.52013	7.110536	-4.11522	4.42E-05	0.000172	0.797247
CDKN3	0.521475	7.083685	4.099818	4.72E-05	0.000183	0.735833
ZNF813	-0.51056	7.051223	-4.07203	5.30E-05	0.000203	0.625546
MMP1	0.85801	6.826843	4.036856	6.14E-05	0.000231	0.487006

GGH	0.592522	8.098832	3.999879	7.15E-05	0.000265	0.342572
TCEAL4	-0.6256	10.43129	-3.95354	8.64E-05	0.000312	0.163349
DIO1	-0.65525	5.362581	-3.8549	0.000129	0.000449	-0.21155
COL11A1	-0.70237	8.453076	-3.84031	0.000136	0.000473	-0.26627
H2AFJ	-0.52926	7.392434	-3.83924	0.000137	0.000475	-0.27028
CALML5	0.738645	6.704357	3.823949	0.000145	0.000501	-0.32735
TNNT1	-0.59211	6.647875	-3.82181	0.000147	0.000505	-0.33531
ZNF160	-0.57924	7.659492	-3.79779	0.000161	0.000549	-0.42454
SERPINA6	-0.55483	4.662846	-3.76054	0.000187	0.000626	-0.5618
MSMB	-0.53796	4.81721	-3.75926	0.000188	0.000629	-0.56649
COL2A1	-0.51562	5.200284	-3.75623	0.00019	0.000636	-0.57759
XBP1	-0.52869	10.33811	-3.68241	0.000252	0.000819	-0.84551
BBOX1	0.537772	5.836361	3.679912	0.000255	0.000826	-0.85448
FBXW12	-0.5846	7.203081	-3.66425	0.000271	0.000871	-0.91065
CP	0.638164	6.576715	3.629831	0.000308	0.000979	-1.03324
ARHGAP29	-0.52591	7.163747	-3.62338	0.000316	0.001	-1.05608
CHRDL1	0.511032	7.425124	3.609998	0.000332	0.001045	-1.10338
DUSP4	-0.52089	8.497033	-3.60088	0.000344	0.001077	-1.13551
CSTA	0.548675	8.559299	3.569418	0.000387	0.001195	-1.24576
MGP	-0.53534	11.66693	-3.54287	0.000427	0.001306	-1.33805
KRT19	-0.6099	10.13604	-3.47586	0.000547	0.001631	-1.56812
DDAH1	-0.50065	7.787114	-3.4475	0.000606	0.00179	-1.66423
SERPINB6	-0.54462	7.436353	-3.41636	0.000679	0.001976	-1.76887
SOX11	0.508135	6.091456	3.392678	0.000739	0.002137	-1.84784
EN1	0.513469	5.675737	3.316451	0.000968	0.00272	-2.09845
TCN1	-0.60963	6.27866	-3.27623	0.001114	0.003076	-2.22848
ELF5	0.566468	6.227691	3.273804	0.001124	0.003101	-2.23626
ZNF91	-0.52769	7.863352	-3.26749	0.001149	0.003165	-2.25651
GABRP	0.71202	7.709075	3.166686	0.001622	0.004309	-2.57483
PMP22	-0.58306	8.514176	-3.15874	0.001666	0.00441	-2.5995
IGHG1	0.736176	5.242477	2.999973	0.002815	0.007007	-3.08008
KRT6A	0.569758	5.040791	2.828407	0.004838	0.011294	-3.57236
S100A7	0.716491	5.980586	2.826762	0.004863	0.01134	-3.57694
SCGB1D2	-0.74562	7.859455	-2.7662	0.00585	0.013321	-3.74388
SCGB2A2	-0.78924	10.11289	-2.71399	0.006844	0.015332	-3.88498
RHOB	-0.53635	7.794269	-2.47131	0.013745	0.028159	-4.50634
