# Supplementary material for: ATP2C2 Has Potential to Define Tumor Microenvironment in Breast Cancer
Source: Front Immunol. 2021 Apr 14;12:657950. doi: 10.3389/fimmu.2021.657950 (PMC8079766; doi:10.3389/fimmu.2021.657950)
Supplement: Supplementary file 2 [file DataSheet_2.pdf]

id	logFC	AveExpr	t	P.Value	adj.P.Val	B
OLFML1	1.649707	6.930069	22.84731	4.41E-83	5.28E-79	178.4841
GLT8D2	1.623757	8.313142	22.16376	1.71E-79	1.02E-75	170.2868
CTSK	1.65795	11.00417	21.64999	8.42E-77	2.67E-73	164.1385
SERPINF1	1.38423	10.65813	21.64527	8.91E-77	2.67E-73	164.0821
CXCL12	1.594582	9.340667	21.44154	1.04E-75	2.48E-72	161.6482
OMD	2.210011	6.21798	21.1445	3.69E-74	7.36E-71	158.1041
SFRP4	2.272276	8.627152	21.09915	6.36E-74	1.09E-70	157.5636
GAS7	1.011704	7.076307	21.00986	1.86E-73	2.78E-70	156.4997
LRRC32	1.392355	7.791579	20.83428	1.53E-72	2.03E-69	154.4097
ECM2	1.326506	7.204347	20.12438	7.43E-69	8.48E-66	145.9863
C1R	1.20125	10.08006	20.12008	7.82E-69	8.48E-66	145.9355
ZCCHC24	1.363531	8.791582	20.11313	8.50E-69	8.48E-66	145.8532
VCAN	1.667853	10.22492	19.86981	1.54E-67	1.42E-64	142.9778
FBN1	1.471981	9.184823	19.59044	4.26E-66	3.64E-63	139.6846
NID2	1.494307	8.633354	19.37692	5.35E-65	4.27E-62	137.1741
LOXL1	1.342528	9.219215	19.30532	1.25E-64	9.34E-62	136.3335
DACT1	1.740125	7.007571	18.79792	4.95E-62	3.49E-59	130.3968
CD93	1.060401	8.240615	18.51495	1.37E-60	9.11E-58	127.1024
HTRA1	1.513344	10.63465	18.40866	4.75E-60	2.99E-57	125.8683
SPARCL1	1.568823	11.3198	18.3325	1.16E-59	6.92E-57	124.9852
MFAP4	2.395064	6.856066	18.26949	2.42E-59	1.38E-56	124.2552
PDGFRL	1.549523	8.180361	18.25277	2.94E-59	1.60E-56	124.0617
F13A1	1.661031	8.422962	17.75178	9.92E-57	5.16E-54	118.2845
SRPX	1.560379	8.820557	17.42216	4.46E-55	2.22E-52	114.5093
PTRF	1.247069	8.513412	17.3869	6.69E-55	3.20E-52	114.1067
ZFPM2	1.389467	6.567842	17.34571	1.07E-54	4.94E-52	113.6368
FAP	1.511734	8.536775	17.13855	1.16E-53	5.13E-51	111.2788
DPT	1.452926	7.815564	17.03337	3.85E-53	1.65E-50	110.0852
OGN	2.71015	6.72219	16.76472	8.23E-52	3.40E-49	107.0476
OLFML2B	1.189139	8.601703	16.6933	1.85E-51	7.39E-49	106.2429
IGFBP6	1.129438	7.805281	16.65891	2.74E-51	1.06E-48	105.8558
ALDH1A1	1.665625	7.156802	16.62644	3.96E-51	1.48E-48	105.4905
COL15A1	1.370792	9.443026	16.45981	2.61E-50	9.45E-48	103.6205
TGFB1I1	1.158445	8.947354	16.41196	4.47E-50	1.57E-47	103.0847
SPOCK1	1.761337	7.85046	16.27029	2.21E-49	7.54E-47	101.5019
WISP2	1.776462	7.135452	16.24092	3.07E-49	1.02E-46	101.1744
THBS2	1.420034	10.86733	16.20265	4.72E-49	1.53E-46	100.7479
JAM2	1.126261	7.196279	16.17418	6.49E-49	2.04E-46	100.4309
CFH	1.395304	7.883996	16.14393	9.11E-49	2.80E-46	100.0944
F2R	0.941804	7.446266	16.12543	1.12E-48	3.36E-46	99.88866
BICC1	0.893851	6.32647	16.07648	1.94E-48	5.66E-46	99.34476
ANGPTL2	1.096324	7.563802	16.06563	2.19E-48	6.16E-46	99.22428
HTR2B	1.600063	5.656085	16.06471	2.21E-48	6.16E-46	99.2141

LUM	1.363773	10.9703	15.97058	6.34E-48	1.73E-45	98.17027
GEM	1.340559	9.152872	15.92153	1.10E-47	2.92E-45	97.6273
CPA3	2.183863	7.621577	15.83169	2.98E-47	7.76E-45	96.63459
CILP	2.153293	9.405533	15.73608	8.63E-47	2.20E-44	95.58046
TCF4	0.977729	9.438209	15.57785	4.99E-46	1.24E-43	93.84161
IGF1	1.371943	7.342674	15.52168	9.27E-46	2.26E-43	93.22617
NAP1L3	1.418527	6.238253	15.38369	4.24E-45	1.02E-42	91.71795
S1PR1	0.944068	6.646851	15.35651	5.72E-45	1.34E-42	91.42152
CD99	0.86942	10.62502	15.34604	6.42E-45	1.48E-42	91.30736
EMCN	1.428556	6.836727	15.33567	7.19E-45	1.62E-42	91.19443
OLFML3	1.263743	9.415555	15.24518	1.94E-44	4.30E-42	90.2098
ITGBL1	1.222461	7.615078	15.0485	1.67E-43	3.63E-41	88.0783
HEG1	0.93312	8.566096	14.97526	3.70E-43	7.90E-41	87.28767
ASPN	1.911329	9.293933	14.9601	4.36E-43	9.16E-41	87.12419
COL8A2	1.287642	7.532454	14.95737	4.49E-43	9.27E-41	87.09476
AXL	0.957623	7.459112	14.89028	9.31E-43	1.89E-40	86.37244
COL6A3	1.32548	12.14755	14.85105	1.42E-42	2.84E-40	85.95081
MN1	1.448213	6.667697	14.83502	1.69E-42	3.32E-40	85.77853
CTGF	1.730136	10.30943	14.82921	1.80E-42	3.48E-40	85.71617
CD200	0.883416	6.893425	14.78452	2.93E-42	5.56E-40	85.23673
CD248	0.845364	8.343162	14.74685	4.40E-42	8.22E-40	84.83311
FBLN1	0.963718	7.340457	14.70367	7.01E-42	1.29E-39	84.37096
AEBP1	1.419481	10.54764	14.67712	9.33E-42	1.69E-39	84.0871
LTBP2	0.954304	9.09677	14.67113	9.95E-42	1.78E-39	84.02316
FAT4	1.207524	5.940509	14.62934	1.56E-41	2.75E-39	83.5769
PRRX1	1.181316	8.170053	14.56396	3.15E-41	5.47E-39	82.88001
LHFP	1.314879	8.497961	14.49993	6.27E-41	1.07E-38	82.19896
ISLR	0.974757	8.51182	14.48514	7.34E-41	1.24E-38	82.0419
THY1	0.821012	9.064003	14.45976	9.64E-41	1.60E-38	81.77238
EML1	0.836345	6.118813	14.41287	1.59E-40	2.61E-38	81.27522
FOXF2	1.014036	6.293215	14.37491	2.39E-40	3.86E-38	80.87324
TSPAN4	0.886909	8.129949	14.36835	2.56E-40	4.09E-38	80.80378
CDH11	1.244155	8.678355	14.35726	2.88E-40	4.54E-38	80.68654
CAV1	1.374342	9.490003	14.25082	8.95E-40	1.39E-37	79.56278
SNAI2	1.086398	9.201338	14.18674	1.77E-39	2.71E-37	78.88813
PDGFRB	0.956473	8.675755	14.16056	2.33E-39	3.54E-37	78.61292
KIAA1462	0.783199	6.181118	14.11343	3.85E-39	5.75E-37	78.11818
DPYSL3	1.10004	7.362114	14.10853	4.05E-39	5.98E-37	78.06679
NBL1	1.137203	8.942319	14.10165	4.36E-39	6.36E-37	77.99467
SPON1	1.107218	6.929197	13.96355	1.87E-38	2.70E-36	76.55015
LAMP5	1.461806	7.20202	13.86698	5.16E-38	7.35E-36	75.54425
TPST1	0.72585	7.303598	13.8575	5.70E-38	8.03E-36	75.44572
TSPAN7	1.314804	6.552023	13.85221	6.03E-38	8.33E-36	75.39069
ACKR1	1.640402	7.126615	13.85173	6.06E-38	8.33E-36	75.38574

RCAN2	0.824479	7.469698	13.84606	6.43E-38	8.74E-36	75.32684
CPE	1.341455	8.918289	13.79243	1.13E-37	1.52E-35	74.77011
PDE2A	0.904324	6.042953	13.71966	2.41E-37	3.21E-35	74.0165
EDNRA	1.007716	7.93938	13.64219	5.41E-37	7.11E-35	73.21651
ANXA5	0.73004	11.39893	13.63934	5.57E-37	7.24E-35	73.18719
RUNX1T1	0.77227	5.573909	13.60624	7.86E-37	1.01E-34	72.84602
COL3A1	1.064987	12.35495	13.60341	8.09E-37	1.03E-34	72.8169
SEMA5A	1.139722	7.275675	13.58626	9.67E-37	1.22E-34	72.64037
FHL1	1.088711	7.21407	13.54395	1.50E-36	1.87E-34	72.20539
ID3	1.119016	8.489312	13.50431	2.26E-36	2.79E-34	71.79845
MFAP2	1.147068	9.088731	13.50173	2.32E-36	2.84E-34	71.77199
ADRB2	0.942649	6.486513	13.46763	3.31E-36	4.00E-34	71.42246
AOX1	0.940672	5.645684	13.46046	3.56E-36	4.26E-34	71.34903
MFAP5	1.442968	8.320578	13.38161	8.03E-36	9.52E-34	70.54282
MXRA5	1.182057	11.11449	13.37805	8.33E-36	9.78E-34	70.50647
HRH1	0.628931	5.918239	13.36872	9.17E-36	1.07E-33	70.41123
COLEC12	1.180282	8.282792	13.33425	1.31E-35	1.50E-33	70.0598
LAMA2	0.950882	6.166487	13.32122	1.50E-35	1.70E-33	69.92705
PKD2	0.885773	7.915691	13.28354	2.20E-35	2.49E-33	69.54364
MAGEL2	0.9749	4.466605	13.26126	2.77E-35	3.10E-33	69.31709
CH25H	1.273671	6.196395	13.23663	3.56E-35	3.95E-33	69.06707
DPYSL2	0.802988	10.36904	13.20836	4.76E-35	5.22E-33	68.78029
PROS1	1.255001	7.572309	13.20152	5.10E-35	5.55E-33	68.71089
SLIT2	0.970515	7.902494	13.18389	6.11E-35	6.59E-33	68.53228
PCOLCE	1.064474	9.586613	13.16667	7.29E-35	7.79E-33	68.35802
POLD2	-0.70107	8.768545	-13.1597	7.83E-35	8.29E-33	68.28722
MRC1	1.36197	7.329689	13.11359	1.25E-34	1.32E-32	67.82132
BACE1	0.640678	7.069548	13.09093	1.58E-34	1.64E-32	67.5926
ADRA2A	1.305919	7.228974	13.08372	1.70E-34	1.75E-32	67.51995
SHOX2	0.667552	5.705759	13.07987	1.77E-34	1.81E-32	67.48104
EFEMP2	0.737562	8.666211	13.06569	2.04E-34	2.07E-32	67.33816
FBLN5	1.194283	8.115462	13.04278	2.58E-34	2.59E-32	67.10738
NRN1	1.025403	7.366767	12.93884	7.39E-34	7.37E-32	66.06314
PLN	1.053868	5.387819	12.7352	5.75E-33	5.69E-31	64.03065
TM6SF1	0.779144	6.716347	12.72938	6.10E-33	5.98E-31	63.97286
SERPING1	1.016492	10.08071	12.69114	8.95E-33	8.71E-31	63.59331
PRELP	1.038038	7.512336	12.62088	1.81E-32	1.74E-30	62.89768
CPED1	0.676717	6.557829	12.60084	2.21E-32	2.11E-30	62.69966
CORO2B	0.981309	4.709103	12.58864	2.49E-32	2.37E-30	62.57913
FXYD1	1.250829	5.975948	12.55493	3.49E-32	3.28E-30	62.24667
PLSCR4	1.083298	8.115726	12.54406	3.88E-32	3.63E-30	62.13961
HSD17B11	0.866695	8.672787	12.49499	6.33E-32	5.87E-30	61.65672
SLC25A10	-0.66735	7.004878	-12.4492	9.96E-32	9.17E-30	61.20759
LAMB1	0.981538	8.946306	12.44649	1.02E-31	9.35E-30	61.18056

SGCD	0.823046	5.61752	12.40531	1.54E-31	1.39E-29	60.77716
MXRA8	0.952243	9.622028	12.39732	1.66E-31	1.50E-29	60.69888
CLIC2	1.077033	6.722283	12.35131	2.62E-31	2.34E-29	60.24928
TREM2	1.143905	7.262986	12.33086	3.21E-31	2.84E-29	60.04971
COMP	1.688342	8.640525	12.31577	3.72E-31	3.27E-29	59.90251
ZMAT3	0.71129	7.570403	12.30942	3.96E-31	3.46E-29	59.84067
FGL2	1.11846	8.267236	12.23233	8.44E-31	7.32E-29	59.09097
COL1A2	1.049392	12.61474	12.22885	8.74E-31	7.52E-29	59.05716
IL33	1.466526	5.675567	12.21535	9.97E-31	8.53E-29	58.92615
COL6A2	0.809158	9.160295	12.2082	1.07E-30	9.08E-29	58.85683
LPAR1	0.636651	7.522495	12.18649	1.32E-30	1.11E-28	58.64639
TMEM255A	1.054527	5.329792	12.18648	1.32E-30	1.11E-28	58.6463
KERA	1.340365	4.530507	12.1701	1.55E-30	1.29E-28	58.48771
IFFO1	0.645789	7.152858	12.16442	1.64E-30	1.36E-28	58.43277
DCHS1	0.678834	6.644312	12.11281	2.72E-30	2.23E-28	57.934
CENPF	-1.00295	6.149881	-12.0989	3.11E-30	2.53E-28	57.79948
ELK3	0.51046	7.343788	12.0793	3.77E-30	3.05E-28	57.61087
MEIS1	0.708721	6.54511	12.05227	4.90E-30	3.91E-28	57.35064
SOD3	0.868693	6.815416	12.02287	6.52E-30	5.17E-28	57.06791
MARCKS	0.662748	9.456232	12.02067	6.66E-30	5.24E-28	57.0468
FMO1	1.241298	7.157991	12.01044	7.35E-30	5.75E-28	56.94857
GFPT2	0.695531	7.266697	11.99708	8.37E-30	6.51E-28	56.82034
SPARC	1.03831	12.03765	11.98585	9.33E-30	7.21E-28	56.71257
GPR65	1.29704	6.950557	11.93208	1.57E-29	1.21E-27	56.19758
FERMT2	0.712828	8.191084	11.92356	1.71E-29	1.30E-27	56.11612
PAICS	-0.56103	7.798346	-11.8972	2.20E-29	1.67E-27	55.86461
RASSF2	0.781658	7.811549	11.89499	2.25E-29	1.69E-27	55.84321
FYN	0.622901	7.394268	11.88967	2.37E-29	1.77E-27	55.79238
PDGFD	0.934577	7.343994	11.88912	2.38E-29	1.77E-27	55.78717
SELP	0.849834	6.592037	11.86691	2.95E-29	2.18E-27	55.57535
TNFAIP6	0.978363	8.23309	11.84943	3.49E-29	2.56E-27	55.40879
COL5A1	1.105429	9.149537	11.84571	3.61E-29	2.64E-27	55.37331
ELN	1.242033	6.450521	11.82278	4.50E-29	3.27E-27	55.15511
RNASE6	1.212178	7.625096	11.81635	4.79E-29	3.45E-27	55.09403
THBS4	1.464789	8.189721	11.7036	1.41E-28	1.01E-26	54.02519
ZFP36	0.966741	9.483728	11.70289	1.42E-28	1.01E-26	54.01843
NOV	1.007335	6.440402	11.70264	1.42E-28	1.01E-26	54.0161
AQP1	0.939892	8.348531	11.67355	1.88E-28	1.32E-26	53.7414
CYTL1	0.955624	5.036593	11.65897	2.16E-28	1.51E-26	53.60381
KCTD12	0.887126	9.923752	11.65709	2.20E-28	1.53E-26	53.5861
MMP3	1.646991	7.771158	11.63739	2.65E-28	1.84E-26	53.4005
COL5A2	1.338127	10.57919	11.58744	4.27E-28	2.93E-26	52.93065
TLR7	0.895763	6.224212	11.57507	4.80E-28	3.28E-26	52.81452
DDR2	0.792505	7.61267	11.57279	4.90E-28	3.33E-26	52.79308

ITM2A	1.201889	8.34982	11.56968	5.05E-28	3.41E-26	52.76392
TOP2A	-1.22716	7.478104	-11.558	5.64E-28	3.79E-26	52.65421
ADD3	0.941318	9.719276	11.55122	6.01E-28	4.02E-26	52.59076
GREM1	1.209267	7.927461	11.53783	6.83E-28	4.54E-26	52.46527
ZNF106	0.577054	8.34964	11.53419	7.07E-28	4.67E-26	52.43117
CNN3	1.077075	9.491759	11.52889	7.43E-28	4.89E-26	52.38155
PLEKHO2	0.535177	8.101243	11.52149	7.97E-28	5.21E-26	52.31228
AKR1B1	0.647776	9.239688	11.51514	8.46E-28	5.50E-26	52.25284
TIMP3	1.183522	9.697343	11.50203	9.58E-28	6.20E-26	52.13023
SPRY1	0.77398	7.808831	11.49065	1.07E-27	6.86E-26	52.0239
NID1	0.877322	7.300994	11.4786	1.20E-27	7.65E-26	51.91131
MMP19	0.567744	6.277654	11.47774	1.21E-27	7.67E-26	51.9033
YIPF5	0.735791	8.588211	11.47434	1.24E-27	7.88E-26	51.87152
PAFAH1B3	-0.73246	9.152935	-11.4688	1.31E-27	8.26E-26	51.82006
GYPC	0.822616	8.043856	11.46325	1.38E-27	8.66E-26	51.76802
NCAPD2	-0.80321	7.578669	-11.4606	1.42E-27	8.83E-26	51.7435
TRIM22	1.042123	9.461829	11.44692	1.61E-27	9.99E-26	51.61572
GIMAP6	0.952142	7.718523	11.42864	1.91E-27	1.18E-25	51.44545
CDC25C	-0.51711	4.591553	-11.417	2.13E-27	1.31E-25	51.33754
ABCA6	1.109419	5.282051	11.4131	2.22E-27	1.35E-25	51.30081
ATP10D	0.72293	7.535098	11.39666	2.59E-27	1.57E-25	51.14795
CTSO	0.895668	8.502666	11.38078	3.00E-27	1.81E-25	51.00033
CYR61	1.218786	9.33827	11.37026	3.31E-27	1.99E-25	50.90265
TPSAB1	1.085599	7.017221	11.36447	3.50E-27	2.09E-25	50.84898
BNC2	0.78738	6.660357	11.34398	4.24E-27	2.52E-25	50.65893
PHYHIP	0.772678	4.731162	11.34282	4.29E-27	2.54E-25	50.64822
TMEM140	0.593882	7.591097	11.28294	7.50E-27	4.42E-25	50.09418
GUCY1B3	0.533614	7.220418	11.2738	8.17E-27	4.79E-25	50.00982
IFITM3	0.74085	12.30111	11.26816	8.62E-27	5.03E-25	49.95769
RBMS1	0.720569	9.497623	11.26307	9.03E-27	5.25E-25	49.91074
CPVL	0.939349	7.548229	11.25188	1.00E-26	5.80E-25	49.80753
WDHD1	-0.64923	4.931984	-11.2509	1.01E-26	5.82E-25	49.7982
GPR153	0.558267	7.352033	11.24961	1.02E-26	5.87E-25	49.78666
CHRDL1	1.459827	7.425124	11.24744	1.05E-26	5.96E-25	49.76667
NCAPG	-1.1296	6.662853	-11.2418	1.10E-26	6.25E-25	49.71507
C3	1.108808	11.45328	11.2294	1.24E-26	6.98E-25	49.60045
COPZ2	0.824201	7.285111	11.22672	1.27E-26	7.12E-25	49.57576
ADAMTS2	0.804196	6.735704	11.21519	1.41E-26	7.89E-25	49.46964
CLEC11A	0.665009	6.909436	11.19801	1.66E-26	9.22E-25	49.31165
CAV2	0.780821	7.239201	11.18586	1.85E-26	1.03E-24	49.2
PARP8	0.768	6.949741	11.1847	1.87E-26	1.03E-24	49.18937
CDH5	0.746391	7.089033	11.18045	1.95E-26	1.07E-24	49.15039
PCSK5	1.068582	6.340446	11.17539	2.04E-26	1.12E-24	49.10388
RRNAD1	-0.66699	7.257051	-11.1725	2.10E-26	1.14E-24	49.07694

PLXNC1	0.640484	6.953429	11.13997	2.84E-26	1.53E-24	48.77906
CFI	0.88041	6.697277	11.12708	3.20E-26	1.70E-24	48.6611
GAS6	0.77405	8.758392	11.12688	3.20E-26	1.70E-24	48.65925
ASPM	-1.30135	6.272718	-11.1236	3.30E-26	1.75E-24	48.62923
NOP2	-0.68654	8.008462	-11.1159	3.55E-26	1.87E-24	48.55839
SH2B3	0.51476	7.651047	11.04352	6.92E-26	3.63E-24	47.89805
COL14A1	1.108937	6.094535	11.04253	6.98E-26	3.65E-24	47.88899
ENPP2	1.060692	8.315384	11.01895	8.67E-26	4.51E-24	47.67439
COL10A1	1.6383	9.279545	11.00418	9.93E-26	5.15E-24	47.5401
CD1C	1.006543	5.984288	10.99129	1.12E-25	5.72E-24	47.42307
CDC25A	-0.80632	5.318719	-10.9709	1.35E-25	6.87E-24	47.23806
SERINC1	0.665604	10.44907	10.96974	1.36E-25	6.91E-24	47.22744
ANKEF1	-0.86569	5.637827	-10.9341	1.89E-25	9.54E-24	46.90461
MCM7	-0.62634	8.623956	-10.924	2.07E-25	1.04E-23	46.81368
CECR1	1.037833	8.404794	10.89373	2.73E-25	1.37E-23	46.53975
GPRASP1	0.87319	6.489955	10.89145	2.79E-25	1.39E-23	46.51917
DHX9	-0.65205	7.405196	-10.8675	3.47E-25	1.72E-23	46.30284
GJA4	0.543127	6.754937	10.86412	3.58E-25	1.77E-23	46.27263
HLA-DRA	1.213015	11.70093	10.85398	3.93E-25	1.93E-23	46.18133
ACVR1	0.779468	8.810667	10.85393	3.93E-25	1.93E-23	46.18082
LGALS1	0.653038	12.78524	10.8531	3.96E-25	1.93E-23	46.17336
C14orf132	1.11188	7.080357	10.80908	5.91E-25	2.87E-23	45.77751
EMILIN1	0.870242	7.212106	10.80782	5.98E-25	2.90E-23	45.76623
STAT5A	0.604473	7.429377	10.79254	6.87E-25	3.31E-23	45.62902
PMF1	-0.50988	8.679042	-10.7507	1.00E-24	4.82E-23	45.25435
RAMP3	0.774914	7.247953	10.74742	1.03E-24	4.95E-23	45.22477
ID1	1.015518	8.543663	10.73712	1.13E-24	5.41E-23	45.13265
RARRES2	1.020524	9.260731	10.7249	1.27E-24	6.02E-23	45.0235
MAN1C1	0.624256	6.979746	10.72264	1.29E-24	6.12E-23	45.00329
CDC20	-1.28127	7.959298	-10.7176	1.35E-24	6.38E-23	44.95804
GART	-0.5399	6.26113	-10.7057	1.51E-24	7.05E-23	44.85192
PLCH1	-0.7464	4.510534	-10.7043	1.53E-24	7.11E-23	44.83942
HPGDS	0.856368	6.266139	10.70059	1.58E-24	7.32E-23	44.80642
CENPI	-0.86139	4.789084	-10.6975	1.62E-24	7.50E-23	44.77925
ATP8B2	0.748684	7.223345	10.68427	1.83E-24	8.42E-23	44.66093
THOC5	-0.56813	6.179043	-10.6701	2.08E-24	9.53E-23	44.53494
POLR3G	-0.70749	4.789003	-10.6565	2.35E-24	1.07E-22	44.41338
VGLL3	0.89149	6.113761	10.6539	2.40E-24	1.09E-22	44.39056
SSPN	0.737084	7.176197	10.633	2.90E-24	1.32E-22	44.20479
ZNF423	1.114988	7.555078	10.62928	3.00E-24	1.35E-22	44.17177
ALOX5AP	1.237615	8.579316	10.61361	3.45E-24	1.55E-22	44.03265
STIL	-1.06496	7.302342	-10.5987	3.95E-24	1.77E-22	43.90087
LTC4S	0.802878	5.473642	10.59811	3.97E-24	1.77E-22	43.89524
AKAP12	0.900691	6.713287	10.5727	4.98E-24	2.22E-22	43.67025

DPP4	0.803164	5.590365	10.54105	6.62E-24	2.92E-22	43.39044
LOX	0.804661	6.86001	10.51055	8.69E-24	3.82E-22	43.12137
NINJ2	0.830894	7.190428	10.47778	1.16E-23	5.10E-22	42.83288
XRCC2	-0.79946	7.049268	-10.433	1.73E-23	7.57E-22	42.4394
ESPL1	-0.80244	6.725874	-10.4274	1.82E-23	7.92E-22	42.39075
IL10RB	0.641791	8.043628	10.41699	2.00E-23	8.66E-22	42.29936
AURKA	-0.8532	6.60706	-10.4059	2.20E-23	9.52E-22	42.20202
SERPINH1	0.766828	9.924215	10.40112	2.30E-23	9.89E-22	42.16043
TRIM24	-0.51877	7.611612	-10.4004	2.31E-23	9.92E-22	42.15378
PAMR1	0.812145	6.122012	10.39875	2.35E-23	1.00E-21	42.13968
TMEM243	0.783157	8.574119	10.3953	2.42E-23	1.03E-21	42.10944
EVI2B	1.066858	8.232598	10.39233	2.48E-23	1.05E-21	42.08353
CRISPLD2	0.957296	8.325934	10.37801	2.82E-23	1.18E-21	41.95833
EBP	-0.56386	7.359938	-10.3779	2.82E-23	1.18E-21	41.9577
CALCRL	0.688617	6.213904	10.31239	5.03E-23	2.11E-21	41.38626
SEMA3C	0.92117	8.265107	10.28455	6.43E-23	2.66E-21	41.14431
BACH2	0.636763	5.990431	10.28415	6.45E-23	2.66E-21	41.14084
PARPBP	-0.82232	4.783975	-10.2694	7.34E-23	3.02E-21	41.01296
TTF2	-0.5935	5.38618	-10.2569	8.20E-23	3.36E-21	40.90438
PCYT1B	-0.5764	4.136442	-10.2539	8.41E-23	3.44E-21	40.87836
AGK	-0.52232	6.812468	-10.2435	9.22E-23	3.75E-21	40.78807
ITIH5	0.936891	6.369114	10.24318	9.24E-23	3.75E-21	40.78563
FABP4	1.833406	8.355378	10.23693	9.76E-23	3.95E-21	40.73154
KIF23	-0.98035	5.880915	-10.2104	1.23E-22	4.96E-21	40.50249
NEK2	-0.88204	6.338062	-10.2079	1.26E-22	5.06E-21	40.48028
IGFBP3	0.887521	9.163948	10.20001	1.35E-22	5.40E-21	40.41239
CSF1R	0.662157	8.09538	10.18895	1.49E-22	5.93E-21	40.31697
FST	0.847175	6.68768	10.18093	1.59E-22	6.34E-21	40.24778
GINS2	-0.80451	6.894012	-10.1769	1.65E-22	6.54E-21	40.21304
CCDC69	0.701373	6.213028	10.17481	1.68E-22	6.64E-21	40.19503
PLIN1	1.399026	6.896288	10.16766	1.79E-22	7.04E-21	40.13341
BUB1	-0.67554	4.659357	-10.1622	1.88E-22	7.34E-21	40.08609
EZH2	-1.00148	7.522	-10.1463	2.15E-22	8.37E-21	39.94997
WASF2	0.564627	8.793236	10.14089	2.26E-22	8.75E-21	39.90308
WISP1	0.851814	5.813488	10.11227	2.90E-22	1.12E-20	39.65726
DAAM2	0.587334	6.649562	10.10238	3.16E-22	1.22E-20	39.57237
C1S	1.063819	9.823386	10.09825	3.27E-22	1.26E-20	39.53701
HLX	0.525651	6.614543	10.08954	3.53E-22	1.35E-20	39.46238
GMFG	0.693588	8.317252	10.08711	3.61E-22	1.37E-20	39.44155
ITPKC	-0.50926	7.095471	-10.075	4.00E-22	1.52E-20	39.33795
ACVRL1	0.538386	6.495814	10.06656	4.31E-22	1.63E-20	39.26563
EPS8	0.66546	9.264861	10.06098	4.52E-22	1.71E-20	39.2179
RAB11FIP2	0.615821	7.87226	10.05949	4.58E-22	1.72E-20	39.20515
MAN2A1	0.574683	8.427256	10.03053	5.89E-22	2.20E-20	38.95782

KCND2	0.884493	5.216384	10.02622	6.11E-22	2.27E-20	38.92108
HIGD1B	0.762117	5.727643	10.02615	6.11E-22	2.27E-20	38.92048
PLS3	0.938103	10.39623	10.02536	6.15E-22	2.28E-20	38.91375
ACOT8	-0.53614	7.035454	-10.0199	6.45E-22	2.38E-20	38.86688
FMO4	0.619521	6.85752	9.992119	8.20E-22	3.00E-20	38.63053
MRPS15	-0.66434	7.894724	-9.99205	8.20E-22	3.00E-20	38.62996
GZMK	1.323298	7.159408	9.976605	9.37E-22	3.42E-20	38.4986
RPS6KA3	0.6395	7.727269	9.961546	1.07E-21	3.88E-20	38.37068
CHIC2	0.897818	8.016657	9.932283	1.37E-21	4.98E-20	38.12248
GPX3	0.927579	8.707543	9.931124	1.39E-21	5.01E-20	38.11266
AOC3	0.907302	7.629655	9.923554	1.48E-21	5.33E-20	38.04854
MCM3AP-AS1	-0.55976	5.31306	-9.9189	1.54E-21	5.51E-20	38.00915
PSAP	0.551916	11.97065	9.91215	1.63E-21	5.82E-20	37.95202
CSGALNACT1	0.824773	7.483483	9.908737	1.68E-21	5.98E-20	37.92314
PLK4	-0.59035	4.825697	-9.90699	1.70E-21	6.05E-20	37.9084
CCPG1	0.531411	6.130453	9.904671	1.74E-21	6.16E-20	37.88876
SLC15A3	0.731846	7.515096	9.899983	1.81E-21	6.39E-20	37.84912
BNIP3L	0.629835	10.09992	9.872925	2.28E-21	8.01E-20	37.62062
CCNJ	-0.59112	5.917222	-9.86944	2.35E-21	8.22E-20	37.59119
NAA10	-0.66607	8.653531	-9.86362	2.47E-21	8.62E-20	37.54211
NR3C1	0.554071	8.648072	9.853831	2.69E-21	9.34E-20	37.45964
PTGIR	0.722685	5.720321	9.848918	2.80E-21	9.71E-20	37.41825
TIMP1	0.665836	12.07244	9.844355	2.91E-21	1.01E-19	37.37983
LIPA	0.752211	10.23624	9.832715	3.22E-21	1.11E-19	37.28186
ADAM12	0.895907	7.086662	9.831295	3.25E-21	1.12E-19	37.26993
MYBL1	-0.95983	6.622592	-9.81944	3.60E-21	1.23E-19	37.17026
C10orf10	0.659343	7.802361	9.808762	3.94E-21	1.35E-19	37.08055
C3AR1	0.719987	8.517678	9.808658	3.95E-21	1.35E-19	37.07967
NUSAP1	-0.92372	7.838995	-9.80177	4.18E-21	1.42E-19	37.02186
GPR183	0.939784	6.979719	9.799616	4.26E-21	1.44E-19	37.00377
FAM49A	0.550209	6.227763	9.79378	4.48E-21	1.51E-19	36.9548
HSD17B6	1.049769	5.900638	9.790442	4.61E-21	1.55E-19	36.92681
TNN	1.097338	6.495261	9.787595	4.72E-21	1.58E-19	36.90293
KCNJ8	0.574467	6.860866	9.782457	4.93E-21	1.65E-19	36.85986
PRPF3	-0.6134	8.356186	-9.78131	4.98E-21	1.66E-19	36.85021
RBL1	-0.63112	4.97037	-9.77639	5.19E-21	1.73E-19	36.80899
ECT2	-0.78868	6.423952	-9.77363	5.31E-21	1.76E-19	36.78593
RERGL	1.154411	4.817805	9.770929	5.44E-21	1.80E-19	36.76328
MEIS2	0.955672	7.673199	9.753264	6.32E-21	2.08E-19	36.61544
GNG11	0.697653	7.541477	9.750494	6.47E-21	2.13E-19	36.59228
UBL3	0.867873	8.916214	9.747065	6.66E-21	2.18E-19	36.56361
OLR1	1.206016	6.311515	9.742847	6.90E-21	2.26E-19	36.52835
GLI1	0.831862	5.277628	9.73964	7.09E-21	2.31E-19	36.50156
TCN2	0.693381	6.975998	9.733037	7.50E-21	2.44E-19	36.4464

HIST1H2AL	-0.79315	4.035631	-9.71475	8.75E-21	2.84E-19	36.29376
MOSPD3	-0.58639	7.654349	-9.71045	9.08E-21	2.94E-19	36.25792
HOXA5	0.847255	6.571823	9.681587	1.16E-20	3.73E-19	36.01757
PLEKHO1	0.705447	7.801611	9.659242	1.40E-20	4.47E-19	35.83183
RAD51	-0.56326	5.251915	-9.65365	1.47E-20	4.68E-19	35.7854
FAXDC2	0.715982	7.071202	9.651862	1.49E-20	4.73E-19	35.77056
NUP54	-0.60822	5.906988	-9.64302	1.60E-20	5.07E-19	35.69718
SGPP1	0.699115	6.88505	9.635868	1.70E-20	5.37E-19	35.63788
DCN	1.203877	9.806107	9.623505	1.89E-20	5.94E-19	35.53543
TENM4	0.636532	6.261677	9.623288	1.89E-20	5.94E-19	35.53364
FABP2	-0.7737	4.169976	-9.62158	1.92E-20	6.01E-19	35.51947
CSF2RB	0.937604	7.431417	9.617077	1.99E-20	6.23E-19	35.48221
SLC38A2	0.500772	11.01286	9.612612	2.07E-20	6.45E-19	35.44524
CIDEA	0.839715	5.149905	9.605672	2.19E-20	6.82E-19	35.38782
GTSE1	-0.69724	6.425736	-9.59145	2.47E-20	7.66E-19	35.27024
ZDHHC13	-0.5912	6.982461	-9.57865	2.75E-20	8.48E-19	35.16452
EMX2	0.733065	4.568961	9.565204	3.08E-20	9.47E-19	35.05359
RASA1	0.617733	9.00704	9.55622	3.32E-20	1.02E-18	34.97954
TXNIP	0.757238	11.46656	9.55477	3.36E-20	1.03E-18	34.96759
SEC23A	0.638238	8.018766	9.548143	3.55E-20	1.08E-18	34.913
PINLYP	0.627615	5.639717	9.544517	3.66E-20	1.11E-18	34.88314
ITM2B	0.586923	11.89756	9.538322	3.85E-20	1.17E-18	34.83215
HIST1H1D	-0.69817	5.456494	-9.53789	3.87E-20	1.17E-18	34.82859
COL5A3	0.575692	6.670572	9.534859	3.96E-20	1.20E-18	34.80365
LINC01140	0.703521	5.89585	9.52887	4.17E-20	1.25E-18	34.75439
RASL12	0.577342	6.925513	9.495571	5.50E-20	1.65E-18	34.4809
CA9	-0.79804	6.25153	-9.4898	5.77E-20	1.73E-18	34.43355
LRRN3	0.826316	5.623979	9.487473	5.88E-20	1.76E-18	34.4145
CST7	0.797008	6.924711	9.485807	5.96E-20	1.78E-18	34.40084
TOMM40	-0.66625	8.194154	-9.47745	6.39E-20	1.89E-18	34.33239
FMOD	0.873349	9.116237	9.466335	7.01E-20	2.07E-18	34.24137
MRE11A	-0.51128	5.313711	-9.46192	7.27E-20	2.14E-18	34.20521
PARP4	0.559858	9.022223	9.460089	7.38E-20	2.17E-18	34.19025
ATP6V1B2	0.731801	9.206137	9.459141	7.44E-20	2.18E-18	34.1825
GAB2	0.781076	7.653393	9.441333	8.63E-20	2.52E-18	34.03694
MCTP1	0.605529	6.12783	9.440337	8.70E-20	2.53E-18	34.02881
PPIC	0.768436	9.383702	9.434305	9.14E-20	2.66E-18	33.97955
SH3GLB1	0.50576	9.548731	9.432372	9.29E-20	2.69E-18	33.96378
SASH1	0.675852	8.946805	9.432076	9.31E-20	2.69E-18	33.96136
GMDS	-0.65926	7.086848	-9.41849	1.04E-19	3.00E-18	33.85055
FTL	0.505761	13.38318	9.414326	1.08E-19	3.10E-18	33.81658
ENTPD1	0.771166	7.373242	9.409564	1.12E-19	3.21E-18	33.77777
COL1A1	0.995176	9.754949	9.404444	1.17E-19	3.34E-18	33.73607
VAX2	-0.641	5.856026	-9.4013	1.20E-19	3.42E-18	33.71046

RAB31	0.70185	10.78412	9.398916	1.22E-19	3.48E-18	33.69105
HJURP	-0.84563	6.431521	-9.39612	1.25E-19	3.56E-18	33.66829
SULF1	0.788428	9.579208	9.392555	1.29E-19	3.65E-18	33.63928
HHEX	0.730131	6.233091	9.38304	1.40E-19	3.94E-18	33.56188
HOXA4	0.615307	5.482919	9.363761	1.64E-19	4.61E-18	33.40524
FANCI	-0.6316	6.826518	-9.36186	1.66E-19	4.67E-18	33.38981
CHST7	0.694011	5.478688	9.357383	1.73E-19	4.84E-18	33.35347
GIMAP4	0.826056	7.738433	9.342046	1.96E-19	5.45E-18	33.2291
RFWD3	-0.64592	6.821099	-9.32782	2.20E-19	6.11E-18	33.11386
TAGLN	1.043248	10.04933	9.322099	2.31E-19	6.39E-18	33.06756
RHO	-0.50307	5.007898	-9.3098	2.55E-19	7.05E-18	32.96806
DOHH	-0.5297	5.799729	-9.2778	3.31E-19	9.14E-18	32.70971
CORIN	0.773947	5.028762	9.267511	3.61E-19	9.92E-18	32.6268
STEAP1	0.959158	7.874875	9.265803	3.66E-19	1.00E-17	32.61303
DTL	-0.92456	7.705663	-9.25179	4.10E-19	1.12E-17	32.5002
AGBL5	-0.52953	6.537684	-9.25068	4.14E-19	1.12E-17	32.49126
TTC27	-0.53012	7.501597	-9.24242	4.43E-19	1.20E-17	32.42484
SLC52A2	-0.56698	8.263234	-9.23588	4.67E-19	1.26E-17	32.37228
HLA-DMB	0.627918	9.86216	9.229847	4.90E-19	1.32E-17	32.3238
HSPG2	0.845653	7.461947	9.229264	4.93E-19	1.32E-17	32.31912
CAMKMT	-0.53036	5.315357	-9.22784	4.98E-19	1.33E-17	32.3077
RNF2	-0.67186	6.337205	-9.22677	5.03E-19	1.34E-17	32.29908
PKD2L1	0.901565	5.938798	9.209688	5.78E-19	1.54E-17	32.16201
SLC13A1	-0.52478	2.73193	-9.20683	5.91E-19	1.57E-17	32.13907
HLA-DMA	0.761104	9.656969	9.205371	5.98E-19	1.58E-17	32.1274
SHPK	-0.57368	6.6524	-9.19794	6.36E-19	1.67E-17	32.06784
CACNA2D3	0.758823	4.613443	9.190402	6.76E-19	1.77E-17	32.00747
CASQ2	0.734714	5.19712	9.186567	6.97E-19	1.83E-17	31.97677
ELMO1	0.692415	6.61512	9.157145	8.85E-19	2.31E-17	31.74155
OCA2	-0.71522	5.309399	-9.15698	8.86E-19	2.31E-17	31.74023
MS4A4A	0.875407	7.178131	9.14863	9.48E-19	2.46E-17	31.67358
DPEP1	0.737628	5.338106	9.141309	1.01E-18	2.60E-17	31.61518
MMP14	1.020002	7.119677	9.140015	1.02E-18	2.62E-17	31.60486
RGL1	0.594035	8.83498	9.139828	1.02E-18	2.62E-17	31.60337
CD1D	0.758489	5.046299	9.124888	1.15E-18	2.95E-17	31.48431
PHLDA2	-0.55698	6.093824	-9.11874	1.21E-18	3.09E-17	31.43539
PNRC1	0.657077	9.82703	9.10885	1.31E-18	3.34E-17	31.35667
TGFBR2	0.831032	7.677086	9.103358	1.37E-18	3.48E-17	31.313
TMEM212	-0.87076	5.190617	-9.086	1.57E-18	3.99E-17	31.17507
ADH1B	1.138354	6.279849	9.085817	1.58E-18	3.99E-17	31.17365
LINC00339	0.962782	6.370747	9.080747	1.64E-18	4.15E-17	31.13341
UTP14A	-0.61753	6.161933	-9.07235	1.76E-18	4.43E-17	31.06681
BYSL	-0.67039	7.76461	-9.06869	1.81E-18	4.56E-17	31.03778
FBLN2	1.105469	8.116326	9.066811	1.84E-18	4.62E-17	31.02289

FSTL1	1.042937	9.677124	9.060798	1.93E-18	4.83E-17	30.97525
MAFB	0.76927	10.35372	9.033987	2.39E-18	5.97E-17	30.76309
P2RY13	0.7745	5.284071	9.029477	2.48E-18	6.18E-17	30.72745
BTG2	0.858451	8.579707	9.025585	2.56E-18	6.36E-17	30.69669
MKI67	-0.73001	6.625438	-9.01192	2.85E-18	7.08E-17	30.58884
NSUN7	-0.58536	4.766377	-9.00467	3.02E-18	7.47E-17	30.53165
WLS	0.776492	7.631269	9.003907	3.04E-18	7.50E-17	30.52562
KLRB1	0.9424	6.156242	8.986826	3.49E-18	8.57E-17	30.39104
CYP20A1	0.550358	6.581035	8.986386	3.50E-18	8.58E-17	30.38758
CD52	0.968606	8.337885	8.975761	3.81E-18	9.32E-17	30.30397
MICALL2	-0.58072	6.52414	-8.97519	3.83E-18	9.32E-17	30.29947
METTL21B	0.636923	6.79801	8.974096	3.86E-18	9.39E-17	30.29087
DFNA5	0.776466	7.661663	8.970007	3.99E-18	9.68E-17	30.25872
ENTPD6	-0.60388	7.550862	-8.96179	4.26E-18	1.03E-16	30.19417
MS4A6A	0.774267	8.185482	8.945511	4.85E-18	1.17E-16	30.06633
TCL1B	0.857915	5.072235	8.939369	5.09E-18	1.23E-16	30.01816
GLIPR1	0.737302	8.068217	8.934245	5.30E-18	1.27E-16	29.97799
RPL11	0.703465	12.70533	8.933372	5.34E-18	1.28E-16	29.97114
ACSBG1	-0.5373	4.200961	-8.92144	5.87E-18	1.40E-16	29.87768
MAP3K7CL	0.641679	5.267278	8.915365	6.16E-18	1.47E-16	29.83012
MYCT1	0.509982	5.498303	8.912765	6.29E-18	1.50E-16	29.80977
NDNF	0.697653	5.708148	8.912256	6.32E-18	1.50E-16	29.80579
NOX4	0.687418	6.224091	8.906956	6.59E-18	1.55E-16	29.76434
UBE2C	-1.01169	8.714105	-8.89755	7.10E-18	1.67E-16	29.69081
FLAD1	-0.55606	8.101042	-8.896	7.19E-18	1.69E-16	29.67867
CENPM	-0.86995	6.98163	-8.88023	8.14E-18	1.91E-16	29.55558
RBFA	-0.6844	6.758619	-8.87695	8.36E-18	1.95E-16	29.52998
CENPN	-0.66715	5.963166	-8.87402	8.55E-18	2.00E-16	29.50714
TACC3	-0.83865	7.160687	-8.86702	9.04E-18	2.10E-16	29.45258
KDR	0.640205	7.152066	8.854937	9.95E-18	2.30E-16	29.35845
CCL19	1.229813	8.532582	8.853575	1.01E-17	2.32E-16	29.34785
PRC1	-0.9566	8.082051	-8.8514	1.02E-17	2.36E-16	29.33094
CD34	0.882547	7.067892	8.843846	1.09E-17	2.48E-16	29.27215
EPX	-0.63416	4.895146	-8.81286	1.39E-17	3.16E-16	29.03148
KLRG1	0.846315	5.75025	8.800786	1.52E-17	3.45E-16	28.93786
IRF8	0.721621	8.384907	8.799769	1.54E-17	3.47E-16	28.92997
FHOD3	0.771737	5.594242	8.799519	1.54E-17	3.47E-16	28.92804
TMPO	-0.51816	6.890168	-8.7987	1.55E-17	3.49E-16	28.9217
LRMP	0.765883	6.067823	8.785342	1.72E-17	3.86E-16	28.81826
LY96	0.966972	8.279378	8.785021	1.73E-17	3.86E-16	28.81578
TUBG1	-0.62465	8.760251	-8.77492	1.87E-17	4.17E-16	28.73764
PUS1	-0.59905	6.971389	-8.76921	1.95E-17	4.35E-16	28.69354
ADAMTS5	0.718852	6.981438	8.767183	1.99E-17	4.40E-16	28.67785
CEP55	-1.00908	6.864638	-8.76417	2.03E-17	4.50E-16	28.65461

PTGER4	0.61464	6.864432	8.759406	2.11E-17	4.66E-16	28.61779
NRXN1	-0.52079	3.952379	-8.75713	2.15E-17	4.73E-16	28.60024
CABYR	-0.58332	6.271478	-8.7549	2.19E-17	4.81E-16	28.58297
STX5	-0.53669	7.034342	-8.75223	2.23E-17	4.89E-16	28.5624
TRIM52	0.501439	6.725141	8.748447	2.30E-17	5.03E-16	28.53321
CNN1	0.729073	7.308663	8.737957	2.50E-17	5.44E-16	28.45234
HLA-DPA1	0.837367	10.78595	8.718534	2.91E-17	6.31E-16	28.30279
NCKAP1L	0.586577	6.965537	8.717863	2.92E-17	6.33E-16	28.29762
SPRY2	0.721455	7.438427	8.716181	2.96E-17	6.39E-16	28.28468
ROR2	0.569636	5.94638	8.704635	3.24E-17	6.99E-16	28.19592
MYBL2	-0.96363	6.862662	-8.70205	3.31E-17	7.12E-16	28.17607
PTGDS	0.853488	8.212446	8.700376	3.35E-17	7.20E-16	28.1632
MMRN1	0.753826	4.181627	8.693022	3.55E-17	7.61E-16	28.10674
FAS	0.57982	7.085151	8.691993	3.58E-17	7.65E-16	28.09884
LPL	1.035629	8.037258	8.690471	3.62E-17	7.73E-16	28.08716
FCER1G	0.934006	8.672303	8.683645	3.82E-17	8.14E-16	28.03479
PALLD	0.604319	9.244738	8.676324	4.04E-17	8.57E-16	27.97866
BGN	0.975087	9.402597	8.670511	4.23E-17	8.95E-16	27.93412
PCNT	-0.54356	7.079861	-8.66235	4.50E-17	9.52E-16	27.87162
TMEM47	0.775174	8.545712	8.661435	4.54E-17	9.57E-16	27.86462
EXOSC4	-0.51711	8.490568	-8.63108	5.74E-17	1.19E-15	27.6326
RNF19B	0.962679	6.980945	8.629589	5.81E-17	1.20E-15	27.6212
NACAD	0.633471	5.14487	8.628412	5.86E-17	1.21E-15	27.61222
AMPH	0.871536	6.099361	8.625337	6.00E-17	1.24E-15	27.58876
CHST8	-0.60431	6.190901	-8.62302	6.11E-17	1.26E-15	27.57109
SQLE	-0.71031	7.480879	-8.62099	6.21E-17	1.28E-15	27.5556
CELF2	0.541011	7.087281	8.596036	7.53E-17	1.54E-15	27.3655
ASPA	0.610696	4.426918	8.590686	7.85E-17	1.60E-15	27.32479
RPGRIP1L	-0.50581	5.0784	-8.58723	8.06E-17	1.64E-15	27.29849
VWF	0.663879	7.625358	8.584932	8.21E-17	1.66E-15	27.28104
E2F8	-0.82307	5.656533	-8.58215	8.39E-17	1.70E-15	27.25987
ZNF385D	0.703596	4.924102	8.580465	8.50E-17	1.72E-15	27.24709
FEZ1	0.53525	5.540123	8.579684	8.55E-17	1.72E-15	27.24115
AMPD1	0.706102	4.927151	8.576453	8.76E-17	1.77E-15	27.2166
SLC18A2	0.612245	4.528115	8.569537	9.24E-17	1.85E-15	27.16409
MACROD1	-0.59161	6.700135	-8.56175	9.81E-17	1.96E-15	27.105
COL21A1	0.771821	5.508947	8.552387	1.05E-16	2.10E-15	27.034
DNM1	0.554938	6.913509	8.538273	1.18E-16	2.34E-15	26.92708
IFITM2	0.593846	11.84015	8.536673	1.19E-16	2.36E-15	26.91497
MAN1A1	0.640316	8.108256	8.529057	1.26E-16	2.50E-15	26.85735
GSPT2	0.774199	7.095324	8.526176	1.29E-16	2.54E-15	26.83556
LYZ	1.03495	9.984262	8.523443	1.32E-16	2.59E-15	26.8149
SPATA7	0.61956	6.078342	8.495221	1.64E-16	3.20E-15	26.60182
PCDHA9	-0.7265	4.965145	-8.49205	1.68E-16	3.27E-15	26.57793

MEGF6	0.622954	6.352749	8.486058	1.76E-16	3.41E-15	26.53277
PLTP	0.792201	8.314298	8.480176	1.84E-16	3.55E-15	26.48846
TBC1D19	0.746058	5.897793	8.474754	1.91E-16	3.70E-15	26.44764
RGS2	0.796618	9.171032	8.473017	1.94E-16	3.74E-15	26.43457
DONSON	-0.55879	7.30872	-8.46987	1.99E-16	3.82E-15	26.41093
SLC7A5	-0.98315	8.056425	-8.46954	1.99E-16	3.82E-15	26.4084
PEX13	-0.53169	6.707525	-8.4644	2.07E-16	3.96E-15	26.36979
CBX8	-0.51567	5.38906	-8.45637	2.20E-16	4.21E-15	26.30939
EIF5A2	0.539256	5.147811	8.454133	2.24E-16	4.27E-15	26.2926
ZFHX4	0.833713	6.182335	8.452235	2.27E-16	4.33E-15	26.27834
SLC6A15	-0.56957	3.510738	-8.44302	2.44E-16	4.63E-15	26.20916
HOXA10	0.565584	6.158103	8.436464	2.56E-16	4.85E-15	26.15998
ARL2BP	0.637112	8.41265	8.435722	2.58E-16	4.87E-15	26.15442
SDPR	0.684568	5.260119	8.434932	2.60E-16	4.89E-15	26.1485
CCNE2	-0.74952	6.201526	-8.43215	2.65E-16	4.99E-15	26.12761
RALGDS	0.521485	8.09806	8.404282	3.28E-16	6.14E-15	25.919
HBB	1.201654	8.256196	8.394234	3.54E-16	6.60E-15	25.8439
DIO2	0.615229	6.2893	8.376578	4.04E-16	7.53E-15	25.71212
NIPSNAP3B	0.610989	4.77151	8.374778	4.10E-16	7.62E-15	25.69869
LMOD1	0.561148	5.796608	8.370386	4.24E-16	7.85E-15	25.66595
VENTX	0.677537	4.958308	8.36615	4.38E-16	8.10E-15	25.63439
GPNMB	0.731707	10.4162	8.363806	4.46E-16	8.23E-15	25.61693
MRGBP	-0.50082	7.128459	-8.35287	4.84E-16	8.91E-15	25.53553
ZAP70	0.656451	5.468953	8.342239	5.25E-16	9.60E-15	25.45644
UBE2E3	0.915593	9.610461	8.340394	5.32E-16	9.71E-15	25.44273
EVC	0.562948	6.004135	8.340281	5.32E-16	9.71E-15	25.44189
CPQ	0.543429	8.059871	8.335073	5.54E-16	1.01E-14	25.40319
ZNF492	-0.51366	4.662782	-8.33458	5.56E-16	1.01E-14	25.39951
LSP1	0.507141	7.633699	8.322155	6.10E-16	1.11E-14	25.30729
ENO3	-0.5486	4.744276	-8.31755	6.32E-16	1.15E-14	25.27309
TNFRSF17	1.341661	5.496112	8.314615	6.46E-16	1.17E-14	25.25136
CCL21	0.928106	6.549081	8.29544	7.47E-16	1.35E-14	25.10932
BLM	-0.75036	6.51226	-8.28766	7.92E-16	1.42E-14	25.05176
FKBP14	0.532844	7.376224	8.282857	8.21E-16	1.47E-14	25.01626
NR2F1	1.205367	7.769313	8.268564	9.14E-16	1.64E-14	24.91068
BST1	0.597816	5.181573	8.253522	1.02E-15	1.83E-14	24.79972
ADH1C	0.8249	4.829826	8.251959	1.03E-15	1.85E-14	24.7882
TRPC1	0.508502	5.540311	8.248023	1.07E-15	1.90E-14	24.75919
DNM3	0.555056	5.302516	8.247835	1.07E-15	1.90E-14	24.75781
MEF2C	0.600429	7.701813	8.217648	1.34E-15	2.35E-14	24.53573
LBH	0.550827	8.294635	8.217259	1.34E-15	2.35E-14	24.53287
ATP6V0D1	0.523121	9.83224	8.216902	1.35E-15	2.36E-14	24.53026
LAIR1	0.60669	6.728	8.216322	1.35E-15	2.36E-14	24.526
TNNT3	0.507913	5.640217	8.213872	1.38E-15	2.40E-14	24.50801

PPM1H	-0.61919	6.483339	-8.21011	1.42E-15	2.47E-14	24.48041
NR3C2	0.763044	5.930011	8.194342	1.59E-15	2.75E-14	24.36473
MLLT4-AS1	-0.66319	5.109676	-8.19396	1.60E-15	2.76E-14	24.36192
TYROBP	0.667419	9.839423	8.18549	1.70E-15	2.92E-14	24.29988
GPX7	0.654495	7.665777	8.181808	1.75E-15	2.99E-14	24.27291
TRIM68	0.577132	6.987765	8.176184	1.82E-15	3.11E-14	24.23176
TRIP13	-0.85959	7.529861	-8.174	1.85E-15	3.16E-14	24.21579
CBX7	0.649678	7.819751	8.170823	1.90E-15	3.23E-14	24.19254
SCEL	-0.6125	3.141004	-8.1585	2.08E-15	3.54E-14	24.10245
CACNA2D1	0.59287	5.788157	8.153411	2.16E-15	3.66E-14	24.06533
PIBF1	0.696633	7.015844	8.146668	2.27E-15	3.82E-14	24.01611
FOS	1.320681	8.963375	8.142611	2.34E-15	3.93E-14	23.98652
TMEM100	0.902096	5.504208	8.139383	2.40E-15	4.01E-14	23.96298
ARHGAP11A	-0.55406	3.957129	-8.13708	2.44E-15	4.07E-14	23.94618
DPEP2	0.725366	4.878161	8.136638	2.45E-15	4.08E-14	23.94298
NSUN5	-0.51163	8.982583	-8.1299	2.57E-15	4.29E-14	23.89385
SKP2	-0.67821	6.364343	-8.12844	2.60E-15	4.32E-14	23.88323
EXO1	-0.72848	6.169339	-8.12187	2.73E-15	4.53E-14	23.83539
RAMP2	0.571739	6.169354	8.121528	2.74E-15	4.53E-14	23.83293
PRPH2	0.607481	4.954299	8.114681	2.88E-15	4.76E-14	23.78312
PTGER2	0.591907	5.574407	8.111946	2.94E-15	4.85E-14	23.76323
GGT5	0.795927	6.49785	8.111717	2.94E-15	4.85E-14	23.76157
ZNF257	-0.65847	4.096618	-8.11006	2.98E-15	4.90E-14	23.74948
BRCA2	-0.5389	4.832082	-8.10208	3.16E-15	5.19E-14	23.69153
SLC1A2	-0.70689	4.95263	-8.10174	3.17E-15	5.20E-14	23.68908
SCN3A	0.664117	3.768562	8.099837	3.21E-15	5.27E-14	23.67524
CDO1	0.607348	5.816412	8.093545	3.36E-15	5.49E-14	23.62956
RIPPLY3	-0.66045	5.091476	-8.09293	3.38E-15	5.51E-14	23.62508
MORF4L1	0.520544	10.77914	8.089175	3.48E-15	5.65E-14	23.59785
PAM	0.56888	9.523978	8.087114	3.53E-15	5.73E-14	23.5829
GAS1	0.668948	7.054859	8.079661	3.73E-15	6.04E-14	23.52887
UBXN8	0.682266	7.010497	8.077693	3.78E-15	6.12E-14	23.5146
LILRP2	-0.54393	3.995121	-8.06754	4.08E-15	6.56E-14	23.4411
CD86	0.594357	6.603567	8.059172	4.34E-15	6.96E-14	23.38052
POLR2F	-0.53948	7.791275	-8.05781	4.38E-15	7.03E-14	23.37064
TSHZ2	0.750109	6.023682	8.04904	4.67E-15	7.46E-14	23.30728
CLEC10A	0.587563	6.273214	8.047123	4.74E-15	7.56E-14	23.29343
XYLT2	-0.64945	5.808582	-8.04366	4.86E-15	7.74E-14	23.26844
RCN3	0.526315	7.785535	8.034684	5.19E-15	8.25E-14	23.20362
STMN1	-0.67655	6.933164	-8.03073	5.34E-15	8.48E-14	23.17509
HSPB2	0.678774	6.010603	8.028832	5.42E-15	8.59E-14	23.16141
ADIPOQ	1.303711	7.559839	8.026633	5.51E-15	8.72E-14	23.14555
SLC6A3	0.654015	5.519355	8.021558	5.72E-15	9.00E-14	23.10897
PDE6C	-0.50104	2.514542	-8.00865	6.28E-15	9.86E-14	23.01605

GINS1	-0.84642	7.883781	-7.99819	6.78E-15	1.06E-13	22.94078
RACGAP1	-0.71769	8.576111	-7.99785	6.80E-15	1.06E-13	22.93833
SLC16A2	0.734789	6.548901	7.991255	7.14E-15	1.11E-13	22.89094
ARSE	0.626919	5.285032	7.972038	8.21E-15	1.27E-13	22.75302
HLF	0.787551	5.640693	7.96274	8.79E-15	1.35E-13	22.68638
RGS4	0.639246	6.11418	7.960504	8.93E-15	1.37E-13	22.67037
DHX38	0.6313	6.460043	7.958638	9.06E-15	1.38E-13	22.657
PPP1R16B	0.664522	6.289627	7.956582	9.19E-15	1.40E-13	22.64228
POU4F1	-0.53589	3.883778	-7.95367	9.39E-15	1.43E-13	22.62143
LAPTM5	0.652824	10.47466	7.952831	9.45E-15	1.44E-13	22.61544
PLXDC1	0.625992	7.306539	7.946222	9.91E-15	1.51E-13	22.56816
GNL2	-0.60649	8.774058	-7.94532	9.98E-15	1.52E-13	22.5617
SLA	0.661986	7.710599	7.936756	1.06E-14	1.61E-13	22.50049
FXYD2	-0.57024	5.250388	-7.92347	1.17E-14	1.77E-13	22.40566
CLIP3	0.571001	6.153244	7.921051	1.19E-14	1.79E-13	22.38837
APOBR	0.635431	5.246877	7.910821	1.28E-14	1.93E-13	22.31543
HOXA7	0.621419	5.961415	7.909682	1.29E-14	1.94E-13	22.30732
TPX2	-0.91505	7.774257	-7.90696	1.32E-14	1.98E-13	22.28792
SYNDIG1	0.668558	6.467391	7.905601	1.33E-14	1.99E-13	22.27825
DBNDD1	-0.55053	6.336761	-7.89902	1.40E-14	2.09E-13	22.23136
KIF20A	-0.81007	7.077959	-7.89695	1.42E-14	2.12E-13	22.21669
ASGR1	0.568335	4.145909	7.894736	1.44E-14	2.15E-13	22.20091
KIF4A	-0.8285	6.661959	-7.87458	1.67E-14	2.47E-13	22.0577
WFDC1	0.636187	6.059167	7.872715	1.69E-14	2.50E-13	22.04442
VSIG4	0.609508	7.911736	7.869863	1.73E-14	2.55E-13	22.02419
CMA1	0.59749	4.401736	7.868515	1.74E-14	2.57E-13	22.01462
SCN3B	0.500805	4.228125	7.864316	1.80E-14	2.65E-13	21.98483
BTN3A2	0.673033	7.966241	7.863963	1.80E-14	2.65E-13	21.98233
GHR	0.827667	7.198492	7.858908	1.87E-14	2.74E-13	21.94649
ZNF165	-0.66724	6.136309	-7.85661	1.90E-14	2.78E-13	21.93017
BTK	0.502844	6.195699	7.850857	1.98E-14	2.90E-13	21.88945
HRC	0.501059	4.371963	7.843209	2.09E-14	3.05E-13	21.83531
GP9	0.558505	5.434802	7.831417	2.28E-14	3.31E-13	21.75191
TAS2R10	-0.59502	4.147835	-7.83118	2.28E-14	3.31E-13	21.75021
CDT1	-0.65254	5.941431	-7.82764	2.34E-14	3.39E-13	21.72522
RAI2	0.824279	7.395231	7.825882	2.37E-14	3.42E-13	21.7128
ROR1	0.605176	5.709526	7.820028	2.47E-14	3.56E-13	21.67146
SYNC	0.641132	6.676564	7.818646	2.50E-14	3.59E-13	21.6617
PDGFC	0.867773	8.583978	7.817145	2.52E-14	3.62E-13	21.65111
DLGAP5	-0.98314	6.69812	-7.81708	2.52E-14	3.62E-13	21.65062
USP13	-0.51058	7.922306	-7.81528	2.56E-14	3.66E-13	21.63795
FCER1A	0.662489	5.566079	7.811189	2.63E-14	3.77E-13	21.60909
CX3CR1	0.898111	7.134675	7.806768	2.72E-14	3.88E-13	21.57791
F8	0.564289	7.296213	7.803472	2.78E-14	3.96E-13	21.55468

PSRC1	-0.55569	7.089467	-7.79633	2.93E-14	4.16E-13	21.50434
SH2D2A	0.720005	5.839148	7.794399	2.97E-14	4.22E-13	21.49076
CCNB1	-0.86523	8.021495	-7.78469	3.18E-14	4.50E-13	21.42247
UAP1L1	0.512579	5.989301	7.779423	3.31E-14	4.65E-13	21.3854
RHBDF2	-0.63625	7.488593	-7.77771	3.35E-14	4.69E-13	21.37333
CLCA3P	-0.54367	2.694251	-7.77424	3.43E-14	4.80E-13	21.349
F11	-0.55975	3.926344	-7.77119	3.51E-14	4.90E-13	21.32758
AURKB	-0.69577	5.780999	-7.7695	3.55E-14	4.95E-13	21.31566
TBKBP1	0.507763	5.243028	7.766897	3.62E-14	5.03E-13	21.2974
RGS5	0.766327	8.229895	7.75969	3.81E-14	5.27E-13	21.24682
SCNN1G	-0.56436	4.583763	-7.75887	3.83E-14	5.29E-13	21.2411
SKI	0.831004	7.700522	7.754323	3.96E-14	5.46E-13	21.20918
FOXM1	-0.92904	6.603657	-7.75361	3.98E-14	5.48E-13	21.20417
WDR3	-0.65262	7.051598	-7.75289	4.00E-14	5.50E-13	21.19916
FCGR2B	1.045614	7.527058	7.750716	4.06E-14	5.58E-13	21.1839
SPC25	-0.81933	5.353598	-7.74476	4.24E-14	5.80E-13	21.14219
LILRB5	0.5774	4.870514	7.738065	4.44E-14	6.08E-13	21.09529
CASP1	0.72205	7.551502	7.736004	4.51E-14	6.15E-13	21.08086
SGCE	0.712433	8.851416	7.734031	4.57E-14	6.23E-13	21.06706
PES1	-0.53154	6.875976	-7.72456	4.89E-14	6.66E-13	21.00085
CDCA3	-0.8111	6.890489	-7.72328	4.94E-14	6.71E-13	20.99187
LHX5	0.694391	4.529946	7.722766	4.96E-14	6.73E-13	20.98829
CYP3A5	-0.51722	3.934575	-7.71344	5.30E-14	7.17E-13	20.92315
CRY2	0.512822	7.156456	7.712515	5.33E-14	7.21E-13	20.91669
WASF3	0.565494	7.072629	7.709061	5.46E-14	7.37E-13	20.89258
ST20	-0.55539	6.428075	-7.70693	5.55E-14	7.47E-13	20.87768
LRRC15	0.924977	8.05777	7.695799	6.01E-14	8.07E-13	20.8001
ZNF407	-0.51187	4.526176	-7.68935	6.29E-14	8.43E-13	20.75515
TACC2	-0.56179	6.714367	-7.68901	6.30E-14	8.44E-13	20.75279
MYNN	-0.51399	6.469537	-7.68614	6.43E-14	8.60E-13	20.73279
PECAM1	0.638305	8.388427	7.680852	6.68E-14	8.92E-13	20.69603
DCTN6	0.652183	9.172987	7.658497	7.82E-14	1.04E-12	20.54068
PPP1R9A	-0.53284	6.410426	-7.65081	8.26E-14	1.09E-12	20.48737
ATAD2	-0.69447	7.595338	-7.64314	8.72E-14	1.15E-12	20.43415
INHBB	0.678262	8.200253	7.641019	8.85E-14	1.16E-12	20.41948
CHML	-0.56199	5.175484	-7.64072	8.87E-14	1.17E-12	20.41742
SLC6A7	0.661529	4.508266	7.634326	9.28E-14	1.22E-12	20.37313
PLA2G2A	0.904945	7.056723	7.633789	9.32E-14	1.22E-12	20.36941
PLAGL1	0.688886	7.890638	7.631498	9.47E-14	1.24E-12	20.35355
HMOX1	0.604389	8.143358	7.624709	9.93E-14	1.30E-12	20.30658
RAD54B	-0.60599	6.454126	-7.62209	1.01E-13	1.32E-12	20.28849
CCNA2	-0.65558	6.651419	-7.61109	1.09E-13	1.42E-12	20.21247
VWA5A	0.502117	7.734012	7.605155	1.14E-13	1.48E-12	20.1715
C7	0.827078	6.008439	7.590552	1.26E-13	1.63E-12	20.07079

ENC1	0.675165	8.402284	7.589646	1.27E-13	1.64E-12	20.06455
PKM	-0.64022	8.707348	-7.58553	1.31E-13	1.68E-12	20.03618
KIF11	-0.74117	6.774715	-7.58006	1.36E-13	1.74E-12	19.99856
RAB23	0.562401	7.046635	7.577814	1.38E-13	1.77E-12	19.98308
CAD	-0.56988	6.940557	-7.57659	1.39E-13	1.78E-12	19.97463
BFSP1	-0.66101	5.298924	-7.57408	1.42E-13	1.81E-12	19.9574
IL18R1	0.543442	4.944204	7.571671	1.44E-13	1.84E-12	19.94083
NCF4	0.542936	6.251626	7.563317	1.53E-13	1.94E-12	19.88341
PSTPIP1	0.609135	5.593223	7.562191	1.54E-13	1.95E-12	19.87568
SEC22B	-0.55344	10.03751	-7.55973	1.57E-13	1.98E-12	19.85875
FAM189B	-0.59633	7.247192	-7.55872	1.58E-13	2.00E-12	19.85183
RNASE3	0.610582	5.010927	7.551057	1.67E-13	2.10E-12	19.79924
AVL9	-0.50364	6.038921	-7.54437	1.75E-13	2.19E-12	19.75339
IL7R	0.83705	8.160879	7.53753	1.83E-13	2.29E-12	19.7065
LTN1	-0.56746	7.2901	-7.53707	1.84E-13	2.30E-12	19.70338
HIST1H4D	-0.6157	5.039844	-7.52826	1.95E-13	2.43E-12	19.64301
CRTAM	0.738158	5.055819	7.523573	2.02E-13	2.51E-12	19.61095
KIAA1614	0.627605	4.800731	7.522583	2.03E-13	2.52E-12	19.60418
MCM10	-0.83775	5.848219	-7.52155	2.05E-13	2.54E-12	19.59709
ITK	0.747145	6.159723	7.520889	2.06E-13	2.55E-12	19.5926
CD69	0.821721	6.448771	7.517438	2.11E-13	2.61E-12	19.569
GALNT10	0.52033	7.858462	7.515489	2.14E-13	2.64E-12	19.55568
LMAN1L	0.599719	4.805702	7.512341	2.18E-13	2.69E-12	19.53417
EHD2	0.712522	7.263656	7.51122	2.20E-13	2.71E-12	19.52651
DOK5	0.599891	6.567033	7.507649	2.26E-13	2.77E-12	19.50212
CCDC53	0.513315	8.65152	7.505964	2.28E-13	2.80E-12	19.49062
EMP3	0.599242	8.489506	7.49929	2.39E-13	2.93E-12	19.44507
MMP11	0.814556	7.746107	7.49553	2.45E-13	3.00E-12	19.41943
ZFP37	0.528757	5.144066	7.49248	2.51E-13	3.06E-12	19.39863
HDAC2	-0.5405	8.534964	-7.49168	2.52E-13	3.08E-12	19.39319
TIMP2	0.822752	9.574348	7.490506	2.54E-13	3.10E-12	19.38517
PBX3	0.517245	8.318048	7.478064	2.77E-13	3.36E-12	19.30043
EDNRB	0.573485	6.201976	7.473726	2.86E-13	3.46E-12	19.27091
LRRC8B	-0.54673	6.221569	-7.4709	2.91E-13	3.52E-12	19.2517
KIFC1	-0.63273	6.850021	-7.46371	3.06E-13	3.69E-12	19.20282
BIN2	0.75907	6.142946	7.460673	3.13E-13	3.77E-12	19.18217
HEATR1	-0.53321	7.710914	-7.45515	3.25E-13	3.91E-12	19.14466
NRGN	0.554897	5.221719	7.453165	3.29E-13	3.96E-12	19.13119
HN1	-0.613	9.263203	-7.44721	3.43E-13	4.10E-12	19.09076
SPRR2B	-0.6601	4.923511	-7.44068	3.59E-13	4.28E-12	19.04652
RIMS2	-0.52326	4.730266	-7.42746	3.93E-13	4.67E-12	18.95693
RAG2	-0.58448	2.866093	-7.41955	4.15E-13	4.90E-12	18.90346
RBM15	-0.50666	7.691926	-7.4063	4.55E-13	5.35E-12	18.81389
CWH43	-0.62638	4.025961	-7.40512	4.59E-13	5.39E-12	18.80593

SLC2A1	-0.79125	6.4851	-7.39981	4.76E-13	5.57E-12	18.7701
MNDA	0.79304	7.561543	7.395182	4.91E-13	5.73E-12	18.73889
PPP3R1	0.660412	6.98768	7.395084	4.92E-13	5.73E-12	18.73823
P2RY14	0.543572	6.840744	7.395074	4.92E-13	5.73E-12	18.73816
ANGPT4	0.556691	4.322461	7.390443	5.08E-13	5.91E-12	18.70694
WNT2	0.739889	7.032118	7.383813	5.31E-13	6.15E-12	18.66227
PTPRM	0.598195	8.135702	7.381329	5.41E-13	6.25E-12	18.64554
ATP2C2	-0.51958	6.26424	-7.37966	5.47E-13	6.31E-12	18.63432
BRIP1	-0.67523	4.934987	-7.37899	5.49E-13	6.33E-12	18.62982
APOD	1.244367	10.17163	7.378098	5.53E-13	6.37E-12	18.62379
ARMCX2	0.952129	8.557872	7.37635	5.59E-13	6.43E-12	18.61203
ERMAP	0.530363	5.857779	7.372497	5.74E-13	6.59E-12	18.5861
SNRNP40	0.58288	7.8203	7.360928	6.22E-13	7.12E-12	18.50833
STMN2	0.631945	5.190789	7.358071	6.34E-13	7.24E-12	18.48914
HMGB3	-0.64964	8.052382	-7.34404	6.98E-13	7.95E-12	18.39499
AGPAT4	0.502261	5.380052	7.339596	7.20E-13	8.18E-12	18.3652
CRYBB2	0.58863	4.675174	7.332033	7.58E-13	8.59E-12	18.31453
SLC16A4	0.724404	6.572958	7.319349	8.26E-13	9.34E-12	18.22966
GALR3	0.727527	5.546484	7.301053	9.36E-13	1.06E-11	18.10745
CCDC121	-0.56067	4.94505	-7.28827	1.02E-12	1.14E-11	18.02224
PGLYRP1	0.614888	4.475489	7.285087	1.04E-12	1.17E-11	18.00102
SH3BGRL	0.542468	10.87295	7.280356	1.08E-12	1.20E-11	17.96951
ZNF80	-0.59672	3.072548	-7.27979	1.08E-12	1.21E-11	17.96577
NTHL1	-0.50866	7.374749	-7.27178	1.14E-12	1.27E-11	17.91245
CCNB2	-0.82347	6.584169	-7.26291	1.21E-12	1.34E-11	17.85349
HSPA14	-0.53229	7.891527	-7.26113	1.23E-12	1.35E-11	17.84168
RUNDC3B	0.620645	5.31115	7.261039	1.23E-12	1.35E-11	17.84107
LILRB2	0.554647	5.857635	7.255374	1.28E-12	1.40E-11	17.80345
FXYD6	0.546309	7.981342	7.250386	1.32E-12	1.45E-11	17.77035
RAD21L1	-0.50433	2.94263	-7.22048	1.62E-12	1.75E-11	17.57227
GADD45A	0.756855	8.442485	7.219433	1.63E-12	1.77E-11	17.56538
IGK	1.068615	6.551243	7.214039	1.69E-12	1.83E-11	17.52973
SULT2B1	-0.50149	6.322281	-7.20708	1.77E-12	1.92E-11	17.48381
CYP1B1	0.654257	8.108801	7.206766	1.77E-12	1.92E-11	17.48171
IKBKG	0.576873	7.24336	7.201332	1.84E-12	1.98E-11	17.44585
RHBG	0.685916	5.488747	7.198813	1.87E-12	2.01E-11	17.42924
GPR162	0.523877	4.785444	7.197894	1.88E-12	2.02E-11	17.42318
SLC43A1	0.511444	6.876184	7.195918	1.91E-12	2.04E-11	17.41015
SERPINE1	0.762897	6.692391	7.193944	1.93E-12	2.07E-11	17.39714
CXCL14	1.114981	9.465915	7.19287	1.95E-12	2.08E-11	17.39006
PLEKHB1	-0.59382	5.682583	-7.18807	2.01E-12	2.15E-11	17.35843
PAF1	-0.54589	8.049607	-7.18632	2.04E-12	2.17E-11	17.34692
HCLS1	0.66227	8.454018	7.180212	2.12E-12	2.26E-11	17.30671
LGMN	0.631312	9.833427	7.174868	2.20E-12	2.33E-11	17.27155

COL17A1	0.764991	4.695175	7.17033	2.27E-12	2.40E-11	17.24172
GRIP2	0.547132	5.306483	7.16013	2.43E-12	2.56E-11	17.17472
LRRC17	0.885579	6.493014	7.147767	2.64E-12	2.77E-11	17.09361
COASY	-0.54442	8.618348	-7.11737	3.23E-12	3.35E-11	16.89468
CLEC2B	0.864186	7.969741	7.111361	3.36E-12	3.48E-11	16.85547
SLC9A3R1	-0.67222	9.779794	-7.10775	3.45E-12	3.55E-11	16.83193
LOC101927770	-0.66111	4.865809	-7.09609	3.72E-12	3.83E-11	16.75592
NDN	0.820212	7.496508	7.094894	3.75E-12	3.86E-11	16.74809
CD247	0.651091	6.361144	7.092164	3.82E-12	3.93E-11	16.73031
KCNK5	-0.60412	6.317028	-7.0876	3.94E-12	4.04E-11	16.70062
RPE65	-0.50234	3.012924	-7.0818	4.10E-12	4.18E-11	16.66284
TMC7	-0.64501	4.365125	-7.07667	4.24E-12	4.32E-11	16.62948
OXT	0.553293	4.821334	7.075752	4.26E-12	4.34E-11	16.62353
CD40LG	0.615724	4.645338	7.073728	4.32E-12	4.39E-11	16.61038
MGLL	0.557284	8.729573	7.072077	4.37E-12	4.42E-11	16.59965
ZNF562	-0.57796	6.895565	-7.06683	4.52E-12	4.57E-11	16.56558
F2RL2	0.80292	6.09145	7.056814	4.83E-12	4.87E-11	16.50058
IGLL1	0.510831	5.326669	7.056602	4.84E-12	4.87E-11	16.4992
DEPDC1	-0.73545	5.518359	-7.05036	5.05E-12	5.06E-11	16.45877
ACKR4	0.62863	5.118814	7.039983	5.40E-12	5.41E-11	16.39154
FKBP10	0.874442	6.690516	7.039654	5.42E-12	5.42E-11	16.38941
PPARG	0.594168	6.153681	7.038798	5.45E-12	5.44E-11	16.38387
CNTN1	0.555321	5.359451	7.029703	5.78E-12	5.77E-11	16.32505
ROBO1	0.594675	8.706054	7.020215	6.16E-12	6.14E-11	16.26375
PRPH	0.661477	5.562284	7.019728	6.18E-12	6.15E-11	16.26061
BCL9	0.635936	7.102083	7.011985	6.50E-12	6.45E-11	16.21064
AGRP	0.581134	5.268537	7.006377	6.75E-12	6.68E-11	16.17448
APLNR	0.607638	7.387976	7.003496	6.88E-12	6.80E-11	16.15591
NUAK1	0.641435	8.703251	7.001288	6.98E-12	6.90E-11	16.14168
ATP7B	0.739638	6.209256	6.998084	7.13E-12	7.04E-11	16.12105
CD2	0.752154	7.877363	6.997095	7.17E-12	7.07E-11	16.11468
MFHAS1	0.504266	7.388941	6.98423	7.81E-12	7.66E-11	16.03191
G0S2	0.721605	8.277674	6.976629	8.21E-12	8.04E-11	15.98306
SPDYE2	-0.56162	6.844103	-6.97647	8.21E-12	8.04E-11	15.98207
ZWINT	-0.73318	8.91854	-6.96955	8.60E-12	8.40E-11	15.9376
IL25	0.527212	4.556254	6.966469	8.77E-12	8.56E-11	15.91785
SHARPIN	-0.51284	7.821014	-6.96601	8.80E-12	8.57E-11	15.9149
KIF15	-0.73868	5.765888	-6.96036	9.13E-12	8.87E-11	15.87866
ITGAV	0.633416	10.638	6.957478	9.30E-12	9.02E-11	15.8602
POU2F3	-0.61141	4.565421	-6.95569	9.41E-12	9.12E-11	15.84873
CCND2	0.564197	6.663222	6.946747	9.98E-12	9.63E-11	15.79149
KLF2	0.531159	8.038075	6.934714	1.08E-11	1.04E-10	15.71454
BHMT2	0.506566	5.51423	6.92941	1.12E-11	1.07E-10	15.68066
HIST1H3B	-0.83185	4.514396	-6.90794	1.29E-11	1.22E-10	15.54375

SDC2	0.523528	8.440387	6.906484	1.30E-11	1.23E-10	15.53446
MYL3	0.548207	4.750748	6.906189	1.30E-11	1.23E-10	15.53258
MYL7	0.510967	4.26163	6.903924	1.32E-11	1.25E-10	15.51816
MOCOS	-0.55567	6.797205	-6.89963	1.36E-11	1.28E-10	15.49084
GLRA3	-0.5013	3.56989	-6.88244	1.52E-11	1.43E-10	15.38157
C1QA	0.751351	8.749876	6.87935	1.55E-11	1.45E-10	15.36196
PNOC	0.629887	5.24906	6.873023	1.61E-11	1.51E-10	15.32181
PDE1B	0.580679	4.809859	6.871263	1.63E-11	1.53E-10	15.31065
PCDHB11	0.599717	4.482655	6.86826	1.66E-11	1.56E-10	15.29162
BTC	-0.50345	4.429254	-6.86648	1.68E-11	1.57E-10	15.28037
ASNS	-0.66278	6.835617	-6.86294	1.72E-11	1.60E-10	15.25792
CTSS	0.646285	8.304086	6.852655	1.84E-11	1.71E-10	15.19281
LXN	0.784591	8.516794	6.851494	1.86E-11	1.72E-10	15.18546
NUBPL	0.503332	6.820699	6.851419	1.86E-11	1.72E-10	15.18499
KLF4	0.556669	7.369183	6.844269	1.94E-11	1.80E-10	15.13978
SLC5A2	0.573512	5.237496	6.843944	1.95E-11	1.80E-10	15.13773
GPR137B	0.546638	8.994001	6.841711	1.98E-11	1.82E-10	15.12362
SLC12A5	-0.62902	4.162669	-6.84139	1.98E-11	1.83E-10	15.12159
CCL5	0.804458	8.520577	6.839158	2.01E-11	1.85E-10	15.10749
SHBG	0.565294	4.655459	6.838729	2.02E-11	1.85E-10	15.10479
CAV3	0.620326	5.191344	6.83732	2.03E-11	1.87E-10	15.09589
PLAC8	0.839346	6.53997	6.83616	2.05E-11	1.88E-10	15.08857
CYP27B1	-0.58069	5.324465	-6.83513	2.06E-11	1.89E-10	15.08205
GSTTP1	0.521545	4.294089	6.8321	2.10E-11	1.92E-10	15.06294
TFAP2B	1.214024	7.165357	6.823016	2.23E-11	2.03E-10	15.00565
APRT	-0.5032	8.751769	-6.816	2.33E-11	2.12E-10	14.96148
HTR5A	0.522338	4.184701	6.808549	2.45E-11	2.22E-10	14.91456
CD180	0.511606	6.288285	6.805203	2.50E-11	2.26E-10	14.89351
CD3E	0.795051	5.95934	6.801708	2.56E-11	2.31E-10	14.87154
TTR	0.583501	5.041453	6.789209	2.77E-11	2.50E-10	14.79303
TAC1	0.884403	4.338171	6.788109	2.79E-11	2.51E-10	14.78613
ICK	-0.61564	5.705719	-6.78443	2.86E-11	2.57E-10	14.76308
IL10RA	0.541314	8.100197	6.781323	2.92E-11	2.61E-10	14.74357
SCG5	0.757144	6.801807	6.781195	2.92E-11	2.61E-10	14.74277
ADCK4	-0.51696	5.637157	-6.77978	2.95E-11	2.64E-10	14.73392
GRP	0.898037	6.910225	6.777376	2.99E-11	2.67E-10	14.71883
HOXB2	0.76983	8.598383	6.777313	2.99E-11	2.67E-10	14.71843
C5AR1	0.593495	6.558453	6.774749	3.04E-11	2.71E-10	14.70237
FAM107A	0.599909	6.07059	6.77247	3.09E-11	2.75E-10	14.68809
CYBRD1	1.015348	8.778563	6.768629	3.16E-11	2.80E-10	14.66404
PF4	0.535793	4.741872	6.762205	3.30E-11	2.91E-10	14.62385
FPR3	0.6625	6.985719	6.759309	3.36E-11	2.96E-10	14.60574
FBXO2	-0.52276	6.146362	-6.75697	3.41E-11	3.00E-10	14.59109
CORO1A	0.596933	8.33725	6.755825	3.43E-11	3.02E-10	14.58396

RHBDD3	-0.53036	6.798671	-6.74809	3.61E-11	3.16E-10	14.53562
LRP8	-0.57941	6.386336	-6.73877	3.83E-11	3.34E-10	14.4775
NGFR	0.508878	5.132401	6.736331	3.89E-11	3.39E-10	14.46228
CD33	0.620119	5.337465	6.733365	3.96E-11	3.45E-10	14.4438
PRKCQ	0.584374	5.388456	6.723989	4.21E-11	3.65E-10	14.3854
PSORS1C2	0.55409	5.028957	6.714583	4.47E-11	3.85E-10	14.32689
IGFBP4	0.764672	10.0804	6.712774	4.52E-11	3.89E-10	14.31564
TRAP1	-0.75089	7.818894	-6.70805	4.66E-11	4.00E-10	14.28627
LRRTM4	-0.54064	3.223883	-6.70721	4.68E-11	4.02E-10	14.28108
PLA2G2F	-0.53388	4.503051	-6.70459	4.76E-11	4.08E-10	14.26478
PRR16	0.546918	5.10492	6.703924	4.78E-11	4.09E-10	14.26067
CD163	0.544593	6.989613	6.702603	4.82E-11	4.13E-10	14.25246
UNG	-0.61056	8.05588	-6.702	4.84E-11	4.14E-10	14.24869
KCNS1	-0.72303	5.988707	-6.69827	4.96E-11	4.23E-10	14.22557
NFKBIL1	0.542296	6.088163	6.679781	5.57E-11	4.73E-10	14.111
CCL7	-0.69312	4.549381	-6.67293	5.82E-11	4.92E-10	14.06865
HSD17B10	-0.50254	10.12915	-6.67152	5.87E-11	4.96E-10	14.05992
BCHE	0.818708	4.373977	6.671267	5.88E-11	4.97E-10	14.05834
POLB	-0.73114	7.881176	-6.66636	6.07E-11	5.12E-10	14.02801
EVI2A	0.755849	7.87693	6.664697	6.13E-11	5.16E-10	14.01774
RBMS3	0.531078	6.314923	6.660577	6.30E-11	5.29E-10	13.99229
RRAGB	0.50022	5.849962	6.659811	6.33E-11	5.31E-10	13.98756
TIGD6	-0.5303	4.32891	-6.65967	6.33E-11	5.31E-10	13.98667
IRS2	0.612463	8.70149	6.658895	6.36E-11	5.33E-10	13.98191
SPAG5	-0.57568	7.131624	-6.65082	6.70E-11	5.60E-10	13.93208
CRTAP	0.525539	8.33238	6.647599	6.83E-11	5.70E-10	13.91224
DHRS11	-0.50559	6.621668	-6.64332	7.02E-11	5.85E-10	13.88586
BTN3A3	0.503124	7.379521	6.639042	7.21E-11	6.00E-10	13.85952
GPC4	0.707068	6.869607	6.634413	7.43E-11	6.17E-10	13.83103
QPCTL	-0.62205	5.105519	-6.6322	7.53E-11	6.25E-10	13.81741
GINS4	-0.57088	5.068879	-6.62996	7.64E-11	6.33E-10	13.80365
DPY19L2P2	-0.60416	4.311874	-6.62364	7.95E-11	6.57E-10	13.76478
PLAU	0.524487	7.801385	6.620827	8.09E-11	6.69E-10	13.74751
TLR2	0.607812	7.582062	6.61597	8.34E-11	6.88E-10	13.71768
FKBP4	-0.83694	7.853252	-6.61273	8.51E-11	7.02E-10	13.69782
ZNF140	0.583896	7.3589	6.612306	8.54E-11	7.03E-10	13.6952
HELLS	-0.57422	5.886209	-6.608	8.77E-11	7.21E-10	13.6688
DSPP	-0.65717	4.882938	-6.60684	8.83E-11	7.26E-10	13.66169
MDN1	-0.55915	6.211738	-6.60589	8.89E-11	7.29E-10	13.65583
KAT2B	0.527517	7.227728	6.596243	9.44E-11	7.73E-10	13.59675
ATP8B4	0.522875	4.85657	6.585725	1.01E-10	8.22E-10	13.53239
MS4A2	0.558403	4.492299	6.575884	1.07E-10	8.67E-10	13.47226
INVS	-0.50083	4.714569	-6.56939	1.12E-10	9.01E-10	13.43263
FUT8	0.545965	7.960291	6.560414	1.18E-10	9.49E-10	13.37788

STAP2	-0.51639	8.143446	-6.55824	1.20E-10	9.61E-10	13.36463
CTSZ	0.654502	7.51018	6.553183	1.24E-10	9.90E-10	13.33384
ABCG2	0.544557	5.465005	6.551238	1.25E-10	1.00E-09	13.322
LIN28A	-0.60876	3.862112	-6.54355	1.31E-10	1.05E-09	13.27524
MELK	-0.86463	7.899854	-6.54279	1.32E-10	1.05E-09	13.27063
SETBP1	0.589951	7.706236	6.541484	1.33E-10	1.06E-09	13.26267
PLXDC2	0.741583	8.461314	6.52619	1.46E-10	1.16E-09	13.16979
GPR18	0.766341	5.497298	6.524071	1.48E-10	1.17E-09	13.15694
TK1	-0.59445	7.625311	-6.52042	1.52E-10	1.20E-09	13.1348
ZNF835	0.612418	4.40025	6.519794	1.52E-10	1.20E-09	13.13101
DNMT3B	-0.57692	6.93622	-6.5155	1.56E-10	1.23E-09	13.10501
POLQ	-0.50256	5.558299	-6.50204	1.70E-10	1.33E-09	13.02355
VRK1	-0.5397	7.793208	-6.49679	1.76E-10	1.37E-09	12.9918
HYAL1	0.508352	4.719269	6.496054	1.76E-10	1.38E-09	12.98733
PHGDH	-0.59436	8.041248	-6.48623	1.87E-10	1.46E-09	12.928
FAM53B	-0.55883	6.446345	-6.48534	1.88E-10	1.46E-09	12.92267
DLX4	-0.50772	4.792928	-6.48167	1.93E-10	1.50E-09	12.9005
AASS	0.589152	5.220162	6.471594	2.05E-10	1.59E-09	12.83978
CCNE1	-0.52398	5.90784	-6.46362	2.16E-10	1.66E-09	12.79176
KIF18B	-0.54792	6.956907	-6.46351	2.16E-10	1.66E-09	12.79114
RAX	0.527374	4.950773	6.462701	2.17E-10	1.67E-09	12.78625
FMO3	0.631154	4.939158	6.458736	2.22E-10	1.70E-09	12.76241
APOBEC3B	-0.77238	7.485678	-6.45584	2.26E-10	1.73E-09	12.745
PPEF1	0.59879	5.402883	6.452459	2.31E-10	1.77E-09	12.72468
HSD11B1	0.529821	5.969453	6.44128	2.47E-10	1.88E-09	12.65758
FNDC4	0.558028	5.901898	6.425107	2.73E-10	2.07E-09	12.56068
KCNJ3	-0.81827	4.417163	-6.41754	2.86E-10	2.16E-09	12.51539
SCML1	0.568131	6.330506	6.408468	3.03E-10	2.27E-09	12.4612
PLEKHF1	0.51919	7.017451	6.406992	3.05E-10	2.29E-09	12.45238
SIX3	-0.54582	4.285687	-6.40459	3.10E-10	2.32E-09	12.43805
IRF9	0.566553	9.0503	6.403617	3.12E-10	2.33E-09	12.43224
CCL2	0.756427	8.79973	6.391591	3.35E-10	2.50E-09	12.36053
TPSG1	0.544525	5.859952	6.385733	3.48E-10	2.58E-09	12.32564
PDLIM3	0.504616	6.502821	6.375467	3.70E-10	2.74E-09	12.26457
SCG2	0.55224	4.89306	6.374323	3.73E-10	2.76E-09	12.25777
ADAMTS1	0.566185	6.828053	6.362064	4.02E-10	2.96E-09	12.18496
SCARB1	-0.51387	6.048132	-6.35757	4.13E-10	3.04E-09	12.15833
SH3BP5	0.544943	9.16238	6.348872	4.35E-10	3.19E-09	12.10675
GJA1	0.812395	10.81035	6.346032	4.43E-10	3.24E-09	12.08993
NR0B2	0.513919	4.842912	6.341809	4.54E-10	3.32E-09	12.06493
SH3BP1	0.525863	5.634444	6.332985	4.79E-10	3.50E-09	12.01274
ZNF544	-0.6344	7.337939	-6.32978	4.89E-10	3.56E-09	11.99381
COL16A1	0.594134	8.670938	6.329241	4.90E-10	3.57E-09	11.99062
DHCR7	-0.5199	8.04822	-6.32773	4.95E-10	3.60E-09	11.9817

CKS2	-0.64665	9.924307	-6.32715	4.97E-10	3.62E-09	11.97825
ITGB2	0.562891	7.97072	6.325022	5.03E-10	3.66E-09	11.9657
CD36	0.761897	6.728531	6.32164	5.13E-10	3.73E-09	11.94574
MT1M	0.743678	5.445465	6.320891	5.16E-10	3.74E-09	11.94132
EGR1	0.786346	9.558469	6.314262	5.37E-10	3.89E-09	11.90222
NAP1L2	0.640859	5.037751	6.308781	5.55E-10	4.01E-09	11.86993
ISYNA1	-0.6831	6.776465	-6.30332	5.74E-10	4.12E-09	11.83778
EGFL6	0.752357	6.54786	6.290455	6.20E-10	4.44E-09	11.76211
HIST1H2AM	-0.5799	5.517721	-6.28433	6.43E-10	4.59E-09	11.72613
NEFH	0.636743	5.60487	6.276071	6.76E-10	4.81E-09	11.67767
CHCHD3	-0.67548	7.454441	-6.2537	7.74E-10	5.47E-09	11.54666
PIM2	0.631287	6.771044	6.252965	7.77E-10	5.49E-09	11.54239
CTSW	0.709357	5.502985	6.248041	8.00E-10	5.65E-09	11.51362
ERI2	-0.70604	6.277366	-6.24547	8.13E-10	5.73E-09	11.49859
RND3	0.574982	9.45366	6.242218	8.29E-10	5.82E-09	11.47962
ITGA7	0.551644	5.47794	6.225482	9.16E-10	6.39E-09	11.38206
C5orf42	-0.60285	4.712352	-6.22429	9.23E-10	6.43E-09	11.37514
NUP93	-0.55537	6.826186	-6.21969	9.48E-10	6.60E-09	11.34837
ZFY	-0.54398	3.37027	-6.21824	9.57E-10	6.65E-09	11.33993
REG3A	-0.52306	4.302957	-6.21593	9.70E-10	6.74E-09	11.3265
MME	0.731576	6.265964	6.206328	1.03E-09	7.11E-09	11.27068
SFN	-0.62407	9.301794	-6.20624	1.03E-09	7.11E-09	11.27019
ADIRF	0.853272	9.354447	6.205793	1.03E-09	7.12E-09	11.26758
TFCP2L1	-0.55936	6.558803	-6.20368	1.04E-09	7.20E-09	11.2553
IBTK	-0.53988	6.402736	-6.19667	1.09E-09	7.49E-09	11.21462
CCR2	0.520719	5.467009	6.191707	1.12E-09	7.70E-09	11.18586
BUB1B	-0.63056	7.289002	-6.19008	1.13E-09	7.77E-09	11.17645
SLCO2B1	0.551655	6.62974	6.186758	1.15E-09	7.92E-09	11.15719
FIP1L1	-0.50521	6.988352	-6.18423	1.17E-09	8.02E-09	11.14255
STAT4	0.507381	6.23142	6.18411	1.17E-09	8.02E-09	11.14186
ARL15	0.642247	6.615747	6.178259	1.21E-09	8.27E-09	11.10801
SRGN	0.574448	9.298116	6.175401	1.23E-09	8.39E-09	11.09148
PLA1A	0.539638	5.201216	6.166533	1.30E-09	8.82E-09	11.04023
IGFBP7	0.562363	9.870514	6.166176	1.30E-09	8.83E-09	11.03818
KIF21B	0.612613	5.076025	6.154592	1.40E-09	9.44E-09	10.97134
ITGA2	0.557874	7.539842	6.151939	1.42E-09	9.55E-09	10.95605
RELN	0.522044	5.587662	6.115892	1.76E-09	1.17E-08	10.74887
TLR4	0.521735	5.980448	6.109643	1.82E-09	1.21E-08	10.71306
KPTN	-0.5204	7.146384	-6.09717	1.96E-09	1.29E-08	10.64169
SUSD4	-0.61323	6.475373	-6.0924	2.02E-09	1.33E-08	10.6144
COX7A1	0.619976	7.944629	6.083177	2.13E-09	1.40E-08	10.56176
KRT9	0.527574	4.970124	6.075209	2.23E-09	1.46E-08	10.51632
NUP153	-0.51069	7.95089	-6.07354	2.25E-09	1.47E-08	10.50683
NOSIP	-0.52418	9.091857	-6.06097	2.42E-09	1.58E-08	10.43525

SYCP2	-0.58823	5.437829	-6.04335	2.69E-09	1.73E-08	10.33519
POP7	-0.5746	9.167324	-6.03292	2.86E-09	1.83E-08	10.27607
TFAP2A	-0.57067	7.680849	-6.0315	2.88E-09	1.85E-08	10.26803
SMPDL3B	-0.50919	5.632799	-6.02954	2.91E-09	1.86E-08	10.2569
MYOD1	0.536077	4.669786	6.02836	2.93E-09	1.88E-08	10.25023
QPCT	0.747435	6.970843	6.025132	2.99E-09	1.91E-08	10.23196
MAGEH1	0.595439	8.464747	6.023061	3.02E-09	1.93E-08	10.22025
BANF1	-0.52437	10.14742	-6.02201	3.04E-09	1.94E-08	10.21428
KDELC1	0.516633	6.586901	5.996702	3.53E-09	2.23E-08	10.07145
KIF2C	-0.63574	6.698963	-5.98895	3.69E-09	2.32E-08	10.02781
CADPS2	0.559306	8.263075	5.964875	4.24E-09	2.65E-08	9.892553
E2F4	0.514046	6.845433	5.964573	4.25E-09	2.65E-08	9.890858
FGFR3	-0.53741	5.794196	-5.95536	4.48E-09	2.79E-08	9.839221
LCK	0.52661	6.711861	5.953841	4.52E-09	2.81E-08	9.830726
PDZRN4	0.587782	4.196027	5.949656	4.63E-09	2.87E-08	9.807306
SOCS2	0.505287	6.48701	5.938602	4.93E-09	3.05E-08	9.745507
FAM198B	0.669801	7.858005	5.936671	4.99E-09	3.08E-08	9.734727
POSTN	0.731918	9.089889	5.934289	5.05E-09	3.12E-08	9.721428
CHRNB1	0.527739	5.581063	5.924352	5.35E-09	3.29E-08	9.665997
DUSP6	0.630409	8.741811	5.909489	5.83E-09	3.57E-08	9.58324
IL12RB2	-0.5949	4.597323	-5.90782	5.88E-09	3.60E-08	9.573969
SERPINB3	-0.53204	3.57268	-5.90393	6.02E-09	3.67E-08	9.552362
C1QTNF3	0.534961	6.684159	5.902365	6.07E-09	3.70E-08	9.543641
CBX6	0.503259	8.466992	5.900673	6.13E-09	3.73E-08	9.534242
ABLIM3	0.538008	6.521138	5.900471	6.14E-09	3.73E-08	9.533121
NXN	0.571637	9.461731	5.892194	6.43E-09	3.91E-08	9.487178
CLDN3	-0.64291	8.918182	-5.89177	6.45E-09	3.91E-08	9.484843
POLR3B	-0.60528	6.155231	-5.882	6.82E-09	4.12E-08	9.430679
11-Sep	0.545334	7.659163	5.88024	6.89E-09	4.16E-08	9.420927
UBE3C	-0.71358	6.624233	-5.87166	7.23E-09	4.35E-08	9.373449
SUV39H2	-0.55974	5.425899	-5.86653	7.45E-09	4.47E-08	9.345106
ZNF428	0.579945	5.671118	5.864131	7.55E-09	4.53E-08	9.331844
PRR11	-0.75011	9.486623	-5.86135	7.67E-09	4.59E-08	9.316483
CYP24A1	-0.5948	3.733717	-5.83883	8.72E-09	5.16E-08	9.192347
SGK1	0.605043	9.651658	5.831998	9.06E-09	5.35E-08	9.154783
SCGB2A2	1.658154	10.11289	5.828315	9.25E-09	5.46E-08	9.134543
NCSTN	-0.61388	7.682601	-5.82768	9.29E-09	5.47E-08	9.13107
FAM64A	-0.52692	6.442533	-5.81767	9.83E-09	5.77E-08	9.076094
FILIP1L	0.532467	7.125824	5.80005	1.09E-08	6.32E-08	8.979601
CD19	0.555427	5.278504	5.799405	1.09E-08	6.34E-08	8.976074
EFEMP1	0.624872	8.11612	5.788661	1.16E-08	6.70E-08	8.917358
MTHFD2	-0.53867	8.734532	-5.78801	1.16E-08	6.72E-08	8.913815
IGLL3P	0.737992	9.768072	5.783176	1.19E-08	6.89E-08	8.887424
BIRC5	-0.60544	6.55865	-5.78169	1.20E-08	6.93E-08	8.87929

MATN3	0.798302	5.307735	5.773691	1.26E-08	7.22E-08	8.835715
SUZ12	-0.74042	7.853402	-5.75961	1.36E-08	7.80E-08	8.759104
HIST1H3F	-0.6466	4.012648	-5.75263	1.42E-08	8.07E-08	8.721177
NDC80	-0.72255	6.641558	-5.74445	1.48E-08	8.43E-08	8.676768
HGS	-0.52704	7.67515	-5.72975	1.61E-08	9.10E-08	8.597172
ABCA8	0.654779	5.396614	5.726399	1.64E-08	9.25E-08	8.579022
RCBTB1	-0.50499	5.933431	-5.71977	1.70E-08	9.55E-08	8.543186
KLK11	0.818564	5.909895	5.703706	1.86E-08	1.04E-07	8.456517
EPN3	-0.53104	6.95686	-5.70318	1.87E-08	1.04E-07	8.453682
FKBP9	0.518658	9.337669	5.701808	1.88E-08	1.05E-07	8.446289
CLDN5	0.522073	7.233562	5.701223	1.89E-08	1.05E-07	8.44314
IL2RB	0.511446	7.414917	5.700671	1.89E-08	1.05E-07	8.440166
HIC1	0.602237	4.830156	5.69381	1.97E-08	1.09E-07	8.403229
DIP2A	-0.50887	6.732379	-5.68268	2.09E-08	1.15E-07	8.343381
SPINK1	-0.61898	4.432165	-5.6784	2.14E-08	1.18E-07	8.320434
HMGB2	-0.6461	8.473547	-5.67629	2.17E-08	1.19E-07	8.309089
CEMIP	0.520143	5.706686	5.675419	2.18E-08	1.20E-07	8.304425
NKG7	0.628485	6.351292	5.67417	2.19E-08	1.20E-07	8.297725
NBEA	0.553499	6.690772	5.66804	2.27E-08	1.24E-07	8.264857
RRM2	-0.88549	8.507525	-5.66343	2.33E-08	1.27E-07	8.240154
SELE	0.560521	5.406289	5.647988	2.53E-08	1.37E-07	8.15758
NBAS	-0.54415	7.190024	-5.64303	2.60E-08	1.41E-07	8.131081
DIAPH3	-0.60317	4.339839	-5.63441	2.73E-08	1.47E-07	8.08513
KIF14	-0.61496	6.126381	-5.63279	2.76E-08	1.48E-07	8.076519
CXCR2	-0.50979	4.422081	-5.62663	2.85E-08	1.53E-07	8.043662
SSTR3	0.539415	4.982122	5.625685	2.87E-08	1.54E-07	8.038659
TTK	-0.75349	6.799006	-5.61251	3.08E-08	1.65E-07	7.96858
PHKG2	-0.56937	6.149035	-5.61157	3.10E-08	1.65E-07	7.963591
ANKS1B	-0.51037	4.344597	-5.60594	3.19E-08	1.70E-07	7.933705
CDH1	-0.64312	8.509061	-5.60291	3.25E-08	1.73E-07	7.917673
PCDH8	-0.55334	3.48199	-5.59993	3.30E-08	1.75E-07	7.901873
CLCN6	0.504062	5.985207	5.575062	3.78E-08	1.98E-07	7.770299
MSLN	-0.75803	4.671882	-5.56696	3.95E-08	2.06E-07	7.727571
KRT20	-0.53426	4.011919	-5.56057	4.09E-08	2.13E-07	7.693871
CYP7B1	-0.58223	5.39033	-5.55806	4.15E-08	2.16E-07	7.680667
MMP13	0.745641	6.059382	5.550403	4.32E-08	2.24E-07	7.640366
CDCA8	-0.59141	6.665567	-5.54024	4.57E-08	2.36E-07	7.58698
GDF10	0.543743	4.57712	5.536955	4.65E-08	2.40E-07	7.569723
CRISP2	-0.55938	3.840504	-5.53176	4.78E-08	2.47E-07	7.542483
ECM1	0.544974	8.145318	5.531114	4.80E-08	2.48E-07	7.539092
SAC3D1	-0.50023	8.475097	-5.5192	5.12E-08	2.62E-07	7.476698
HOXC13	0.530015	5.97216	5.519015	5.13E-08	2.63E-07	7.475727
LIAS	0.539245	6.159876	5.517656	5.16E-08	2.64E-07	7.468617
MYH11	0.509973	6.226414	5.503153	5.59E-08	2.84E-07	7.392849

HOXB7	0.514682	6.868215	5.499208	5.71E-08	2.90E-07	7.372267
EGR3	0.647104	7.670859	5.495767	5.81E-08	2.95E-07	7.354326
LY86	0.584901	7.992506	5.490021	5.99E-08	3.03E-07	7.324391
PKIA	0.5358	5.775553	5.47106	6.64E-08	3.33E-07	7.225814
CD8A	0.535237	7.314496	5.470639	6.65E-08	3.34E-07	7.22363
EGR2	0.605071	8.165613	5.465452	6.84E-08	3.43E-07	7.196721
ATP6V0C	0.60301	9.903233	5.446956	7.55E-08	3.76E-07	7.100944
MS4A1	0.683986	5.981247	5.433821	8.10E-08	4.02E-07	7.033105
KIAA0101	-0.63078	8.180942	-5.41281	9.06E-08	4.44E-07	6.924901
SCUBE2	1.073502	8.748075	5.403151	9.54E-08	4.65E-07	6.875287
MAD2L1	-0.55009	6.788551	-5.39118	1.02E-07	4.93E-07	6.813905
GZMA	0.642764	6.892182	5.385505	1.05E-07	5.07E-07	6.784851
SELL	0.633133	6.998906	5.376091	1.10E-07	5.29E-07	6.736714
CTNNAL1	0.537622	7.643752	5.363621	1.18E-07	5.62E-07	6.673067
SNX29P2	-0.50921	4.434256	-5.35393	1.24E-07	5.89E-07	6.623719
ORC6	-0.66283	7.752109	-5.34288	1.31E-07	6.21E-07	6.567493
PIK3R4	-0.55057	5.446701	-5.34199	1.32E-07	6.24E-07	6.562973
DUSP4	0.762237	8.497033	5.337063	1.35E-07	6.38E-07	6.537962
LIMA1	0.510567	8.964772	5.325388	1.44E-07	6.76E-07	6.478758
CAMK2N1	0.679898	8.430173	5.324193	1.45E-07	6.79E-07	6.472706
SUCLG1	-0.56372	8.323079	-5.30248	1.62E-07	7.58E-07	6.362937
CDH19	-0.51534	4.066593	-5.29677	1.67E-07	7.79E-07	6.334159
CNGB3	-0.52117	4.22462	-5.29358	1.70E-07	7.91E-07	6.318047
HPRT1	-0.65295	7.766874	-5.29087	1.72E-07	8.02E-07	6.304427
NPTX2	0.507261	5.122752	5.282422	1.80E-07	8.36E-07	6.261908
PTTG1	-0.72972	8.955671	-5.28167	1.81E-07	8.39E-07	6.258119
HOXC8	-0.51141	5.750634	-5.25944	2.03E-07	9.35E-07	6.14658
VSNL1	0.503585	6.079054	5.242696	2.21E-07	1.01E-06	6.062824
SKA1	-0.50864	4.398562	-5.23236	2.33E-07	1.06E-06	6.011247
UCN	-0.60745	5.463488	-5.21708	2.53E-07	1.14E-06	5.935166
KIF18A	-0.50571	5.206838	-5.21323	2.58E-07	1.16E-06	5.916044
CENPU	-0.56337	7.496682	-5.19795	2.79E-07	1.25E-06	5.840207
EXTL2	0.579202	7.368327	5.191905	2.88E-07	1.29E-06	5.810287
PTTG3P	-0.52888	5.175569	-5.17808	3.09E-07	1.37E-06	5.741951
RAB3GAP2	-0.64117	4.334332	-5.17116	3.20E-07	1.42E-06	5.707805
TDP1	-0.5212	6.757758	-5.16336	3.33E-07	1.47E-06	5.669353
MXI1	0.579675	9.612465	5.146698	3.62E-07	1.58E-06	5.587397
UQCRC1	-0.59331	9.967174	-5.14041	3.74E-07	1.63E-06	5.556561
OBP2A	0.502457	5.129318	5.123424	4.08E-07	1.76E-06	5.473344
RBP4	0.514876	5.23343	5.091592	4.79E-07	2.05E-06	5.318113
INHBA	0.51373	7.656146	5.078496	5.12E-07	2.18E-06	5.254506
CARS2	-0.60972	6.535211	-5.04778	5.98E-07	2.51E-06	5.105908
IGFBP2	0.681553	8.63791	5.04578	6.04E-07	2.54E-06	5.096264
HPGD	0.649263	5.419022	5.042411	6.14E-07	2.58E-06	5.080021

RTN1	0.51731	7.061953	5.017401	6.96E-07	2.90E-06	4.959757
TCEAL2	0.501342	4.603983	5.01559	7.02E-07	2.92E-06	4.951069
LYVE1	0.564438	4.9359	5.013124	7.11E-07	2.95E-06	4.939243
C8orf4	0.7881	7.976466	4.998108	7.66E-07	3.16E-06	4.867355
CTNNB1	0.524341	9.535378	4.992878	7.86E-07	3.24E-06	4.842365
UGT8	-0.60344	4.684808	-4.98932	8.00E-07	3.30E-06	4.825357
HIST1H1C	-0.64838	9.006367	-4.9882	8.04E-07	3.31E-06	4.820012
ZNF160	-0.75386	7.659492	-4.98535	8.16E-07	3.36E-06	4.806417
SOWAHC	0.548611	8.171058	4.975182	8.58E-07	3.52E-06	4.757983
ZNF665	0.787863	7.829338	4.975092	8.58E-07	3.52E-06	4.757558
B4GALT3	-0.50136	7.399429	-4.96377	9.08E-07	3.70E-06	4.703701
REPIN1	-0.50955	8.366362	-4.95137	9.65E-07	3.91E-06	4.644869
LINC00328	-0.61896	3.69074	-4.932	1.06E-06	4.26E-06	4.553245
EPHX2	0.526887	6.375941	4.904887	1.21E-06	4.82E-06	4.425508
RAC3	0.594484	5.285643	4.900334	1.24E-06	4.91E-06	4.404127
HIST1H4H	-0.61468	6.611164	-4.89218	1.29E-06	5.09E-06	4.365867
FMO2	0.522804	7.348885	4.882359	1.35E-06	5.32E-06	4.319882
ZNF813	-0.6062	7.051223	-4.86307	1.49E-06	5.79E-06	4.229815
GUSB	-0.5088	8.635473	-4.85746	1.53E-06	5.93E-06	4.203648
P2RX5	0.541005	5.839978	4.842366	1.64E-06	6.33E-06	4.133467
TMEM209	-0.52753	6.761134	-4.82946	1.75E-06	6.69E-06	4.073602
CDK1	-0.59961	7.189365	-4.82631	1.78E-06	6.79E-06	4.059036
TWSG1	0.567354	7.487726	4.786474	2.15E-06	8.13E-06	3.875316
ZBTB16	0.598022	6.390891	4.778708	2.23E-06	8.42E-06	3.839662
PTN	0.521205	7.003349	4.771179	2.32E-06	8.71E-06	3.805152
ANXA1	0.649885	9.443129	4.750559	2.55E-06	9.55E-06	3.710892
CD48	0.51236	7.468759	4.739006	2.70E-06	1.01E-05	3.658246
DSG1	-0.52348	3.781693	-4.72471	2.89E-06	1.07E-05	3.593271
CLTC	-0.52705	7.556901	-4.68966	3.41E-06	1.25E-05	3.434717
ANKRD36	-0.75446	6.425487	-4.68899	3.42E-06	1.25E-05	3.431695
LMO3	-0.57825	5.106309	-4.65367	4.04E-06	1.46E-05	3.273056
ZNF7	-0.50078	6.207457	-4.648	4.14E-06	1.49E-05	3.247695
RPL27A	-0.57005	8.736489	-4.64034	4.30E-06	1.54E-05	3.213464
XYLB	-0.57855	4.233774	-4.63946	4.31E-06	1.55E-05	3.209554
CFD	0.620148	9.085904	4.633839	4.43E-06	1.58E-05	3.184468
NF1	-0.50839	4.16304	-4.63355	4.43E-06	1.58E-05	3.183197
SCGB1D2	1.226206	7.859455	4.600572	5.17E-06	1.83E-05	3.036659
TLK1	-0.5069	5.926944	-4.59402	5.33E-06	1.88E-05	3.007662
GSTT2	0.515704	5.508003	4.582484	5.62E-06	1.97E-05	2.956704
TFB2M	-0.53715	6.84173	-4.57372	5.85E-06	2.05E-05	2.91808
TMEM134	-0.5134	7.084387	-4.55234	6.46E-06	2.24E-05	2.824119
CHIT1	0.524707	5.163226	4.547249	6.61E-06	2.29E-05	2.8018
KIT	0.540085	7.961993	4.540577	6.81E-06	2.36E-05	2.772594
GPR171	0.563187	5.585343	4.513435	7.71E-06	2.64E-05	2.654188

CD3D	0.515288	8.023247	4.504149	8.05E-06	2.75E-05	2.613835
PARD6B	-0.56346	5.339925	-4.42308	1.16E-05	3.87E-05	2.264823
SLC27A2	-0.55484	5.960176	-4.3816	1.40E-05	4.60E-05	2.088529
HMMR	-0.5293	6.570596	-4.36202	1.52E-05	4.98E-05	2.005816
HIST1H2BM	-0.70228	4.531805	-4.35313	1.58E-05	5.17E-05	1.968407
TRIB1	-0.68022	7.862968	-4.33866	1.69E-05	5.47E-05	1.907651
HIST1H1E	-1.04664	5.607766	-4.32979	1.76E-05	5.67E-05	1.870488
S100A4	0.578831	10.28925	4.326349	1.78E-05	5.76E-05	1.856106
PLAT	0.519603	8.932192	4.317771	1.85E-05	5.96E-05	1.820275
WNT5A	0.521428	7.285172	4.315131	1.87E-05	6.02E-05	1.809257
CRYAB	0.526973	8.584909	4.297558	2.02E-05	6.45E-05	1.736098
TGFBR3	0.528011	8.779286	4.291256	2.08E-05	6.61E-05	1.709931
CD37	0.63219	6.114857	4.282733	2.16E-05	6.85E-05	1.674599
AMIGO2	0.579715	8.996325	4.267336	2.31E-05	7.28E-05	1.610934
EPCAM	-0.53426	11.30263	-4.2624	2.36E-05	7.41E-05	1.590588
AKR1C3	0.612938	8.204496	4.244603	2.55E-05	7.95E-05	1.517333
PSD3	0.535276	7.357475	4.223965	2.78E-05	8.62E-05	1.432767
FABP5	0.512211	9.381477	4.218217	2.85E-05	8.83E-05	1.409279
MEX3C	-0.52711	6.636636	-4.20755	2.99E-05	9.22E-05	1.365794
CGA	-0.53826	4.304083	-4.19175	3.20E-05	9.81E-05	1.301519
PMEPA1	0.584745	8.634196	4.158738	3.68E-05	0.000112	1.168037
GRIA2	-0.69654	4.037961	-4.15262	3.78E-05	0.000115	1.143423
TECR	-0.50277	8.429266	-4.10247	4.67E-05	0.000139	0.942768
LY6D	-0.51433	5.024446	-4.10115	4.69E-05	0.00014	0.937532
FOSB	0.671531	6.35935	4.061103	5.55E-05	0.000163	0.779035
C1orf54	0.517205	8.710455	4.057465	5.63E-05	0.000165	0.764712
HLA-DPB1	0.564224	9.999116	4.007049	6.94E-05	0.0002	0.56743
CRABP2	-0.51258	10.0464	-3.96624	8.21E-05	0.000234	0.409455
CCL4	0.544238	7.374806	3.954958	8.59E-05	0.000244	0.366048
PTPRT	0.506947	5.920532	3.951666	8.71E-05	0.000247	0.353405
AR	0.517628	8.029001	3.945795	8.92E-05	0.000252	0.330881
CST1	0.618996	5.803149	3.934027	9.36E-05	0.000264	0.28583
HSPD1	-0.59497	9.064064	-3.89385	0.00011	0.000306	0.13299
LRRC59	-0.75097	7.728183	-3.87925	0.000117	0.000323	0.077803
ARL4A	0.530971	8.237741	3.878218	0.000117	0.000325	0.073923
ART3	-0.57214	4.416928	-3.86793	0.000122	0.000338	0.03517
NRTN	-0.50567	4.265155	-3.85725	0.000127	0.000351	-0.00493
MKL2	-0.62068	7.302046	-3.82835	0.000143	0.00039	-0.11299
TIMM13	-0.50131	7.54953	-3.77538	0.000176	0.000473	-0.30903
HSD17B12	-0.64688	7.499334	-3.67174	0.000263	0.000685	-0.68502
COMMD8	0.503052	8.554181	3.659268	0.000276	0.000716	-0.72959
FBXW12	-0.58152	7.203081	-3.64442	0.000292	0.000754	-0.78247
BMP4	0.50986	5.818141	3.607265	0.000336	0.000858	-0.91389
RPLP2	-0.60515	5.694836	-3.59845	0.000347	0.000885	-0.94489

MORF4L2	-0.51921	9.987908	-3.55988	0.000401	0.001008	-1.07964
PTBP1	-0.56656	8.507317	-3.49146	0.000517	0.001264	-1.31521
IGHM	0.645896	7.975014	3.486089	0.000527	0.001287	-1.33354
CXCL9	0.609539	9.271134	3.477982	0.000543	0.001322	-1.36112
MMP1	-0.74055	6.826843	-3.47182	0.000555	0.001348	-1.38204
TCN1	0.63621	6.27866	3.42181	0.000665	0.001588	-1.55055
SCGB2A1	0.735127	6.71795	3.365666	0.000814	0.00191	-1.73694
CYP4B1	0.511744	6.275682	3.352009	0.000854	0.001995	-1.78183
KRT14	0.660456	9.074429	3.343665	0.00088	0.002047	-1.80916
MLPH	0.55485	10.35431	3.308083	0.000997	0.002291	-1.92501
IGLJ3	0.599899	7.400054	3.304163	0.001011	0.00232	-1.9377
CXCL10	0.56589	9.387481	3.279499	0.001102	0.002512	-2.01721
SERPINB6	-0.52212	7.436353	-3.27252	0.001129	0.002567	-2.03959
MMP2	0.583235	8.016881	3.224958	0.00133	0.002974	-2.191
NDUFA10	-0.55885	7.051817	-3.1929	0.001484	0.003286	-2.29182
RPS11	-0.53404	9.107415	-3.17266	0.00159	0.003499	-2.355
CXCL11	0.518836	6.352282	3.1721	0.001593	0.003504	-2.35673
NAT1	0.70807	9.3357	3.105909	0.001989	0.004298	-2.56055
PIP	0.754671	10.10035	3.051496	0.00238	0.005069	-2.72497
ELF5	-0.51987	6.227691	-3.00008	0.002814	0.005902	-2.87777
ISG15	-0.50525	10.32223	-2.99271	0.002882	0.006033	-2.89947
PABPC1	-0.52522	10.77376	-2.9769	0.003032	0.006324	-2.94582
LTF	0.633389	9.738503	2.924345	0.003585	0.007367	-3.09823
CLCA2	0.500025	5.306494	2.924109	0.003588	0.00737	-3.09891
KRT6A	-0.57457	5.040791	-2.85255	0.00449	0.009004	-3.30217
CXCL13	0.596973	7.689947	2.832362	0.00478	0.00953	-3.35863
BMPR1B	-0.52139	6.099847	-2.82044	0.004958	0.009833	-3.39178
S100A8	-0.53404	6.753451	-2.81862	0.004986	0.009885	-3.39683
CLGN	0.502263	6.151551	2.759395	0.005972	0.011635	-3.55943
MMP12	-0.53601	6.152958	-2.74035	0.006325	0.01226	-3.61102
