# Supplementary material for: ATP2C2 Has Potential to Define Tumor Microenvironment in Breast Cancer
Source: Front Immunol. 2021 Apr 14;12:657950. doi: 10.3389/fimmu.2021.657950 (PMC8079766; doi:10.3389/fimmu.2021.657950)
Supplement: Supplementary file 3 [file DataSheet_3.docx]

Supplementary Material

# Supplementary Figures and Tables

## Supplementary Figures

**
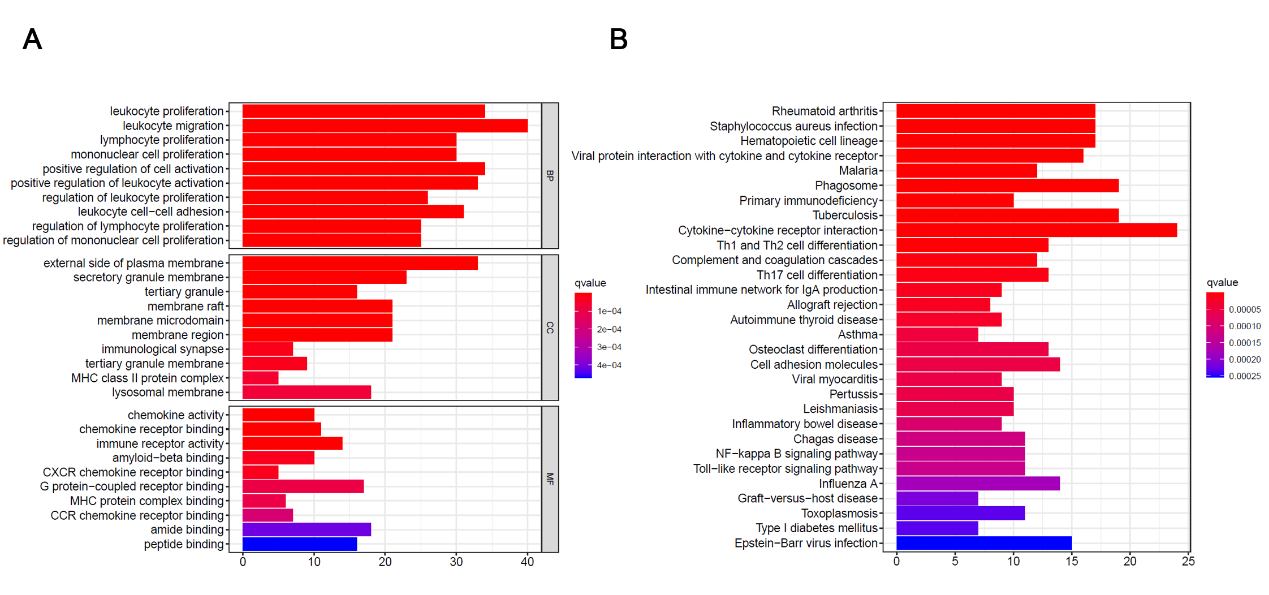
**

**Supplementary Figure 1.** Functional enrichment of DEGs. (A) GO analysis. (B) KEGG analysis.


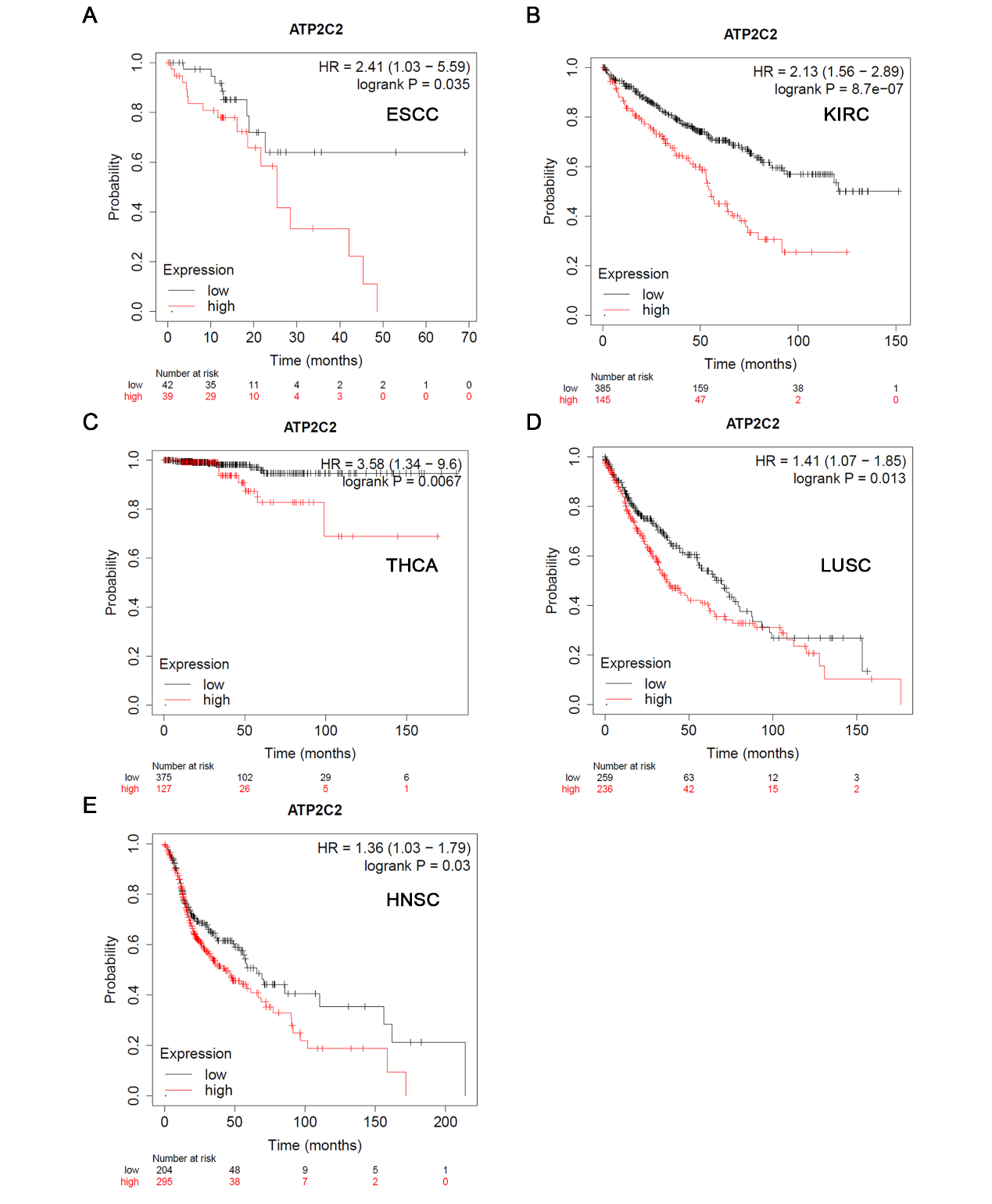


**Supplementary Figure 2.** Kaplan–Meier estimates of OS in various cancer patients with high- and low-expression of ATP2C2 (A–E).


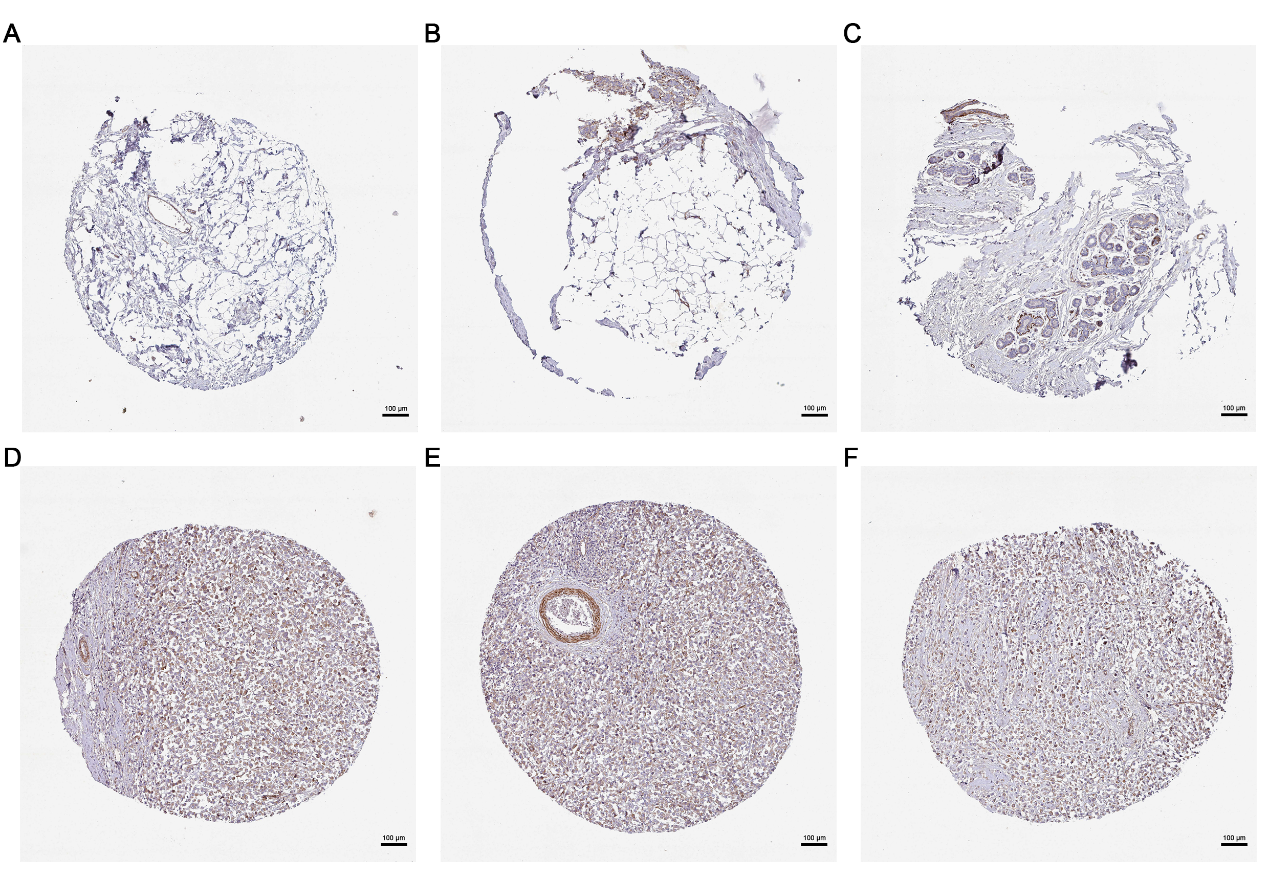


**Supplementary Figure 3.** Validation of ATP2C2 on a translational level using the Human Protein Atlas database. (A–F) The upper one is normal breast and under one is BRCA tissues. Scale bars, 100 μm.

**
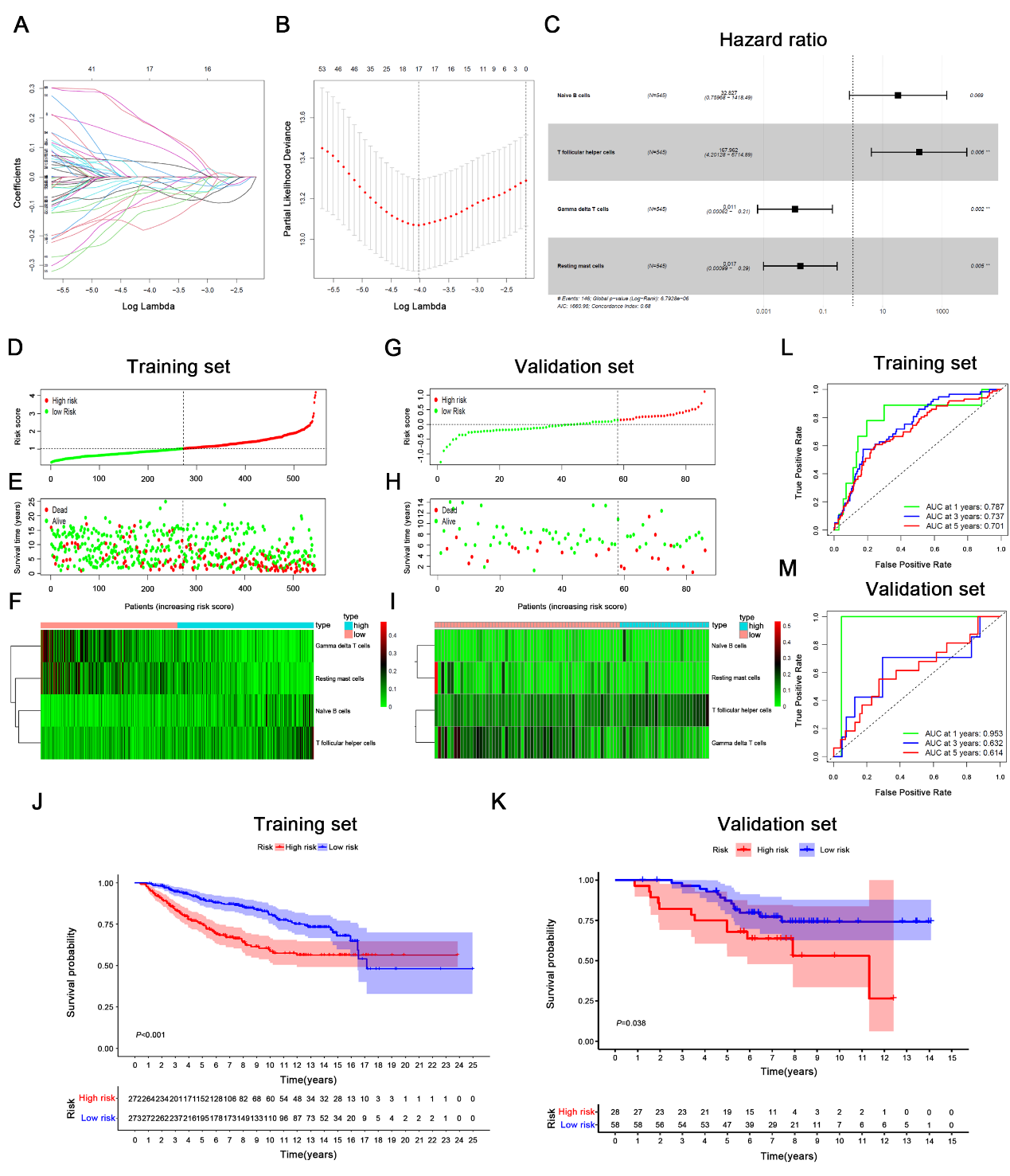
**

**Supplementary Figure 4.** Construction and validation of the immunoscore model based on four TICs (naïve B cells, Tfh cells, γδ T cells, and resting mast cells). (A) LASSO coefficients. (B) Plots of the cross-validation error rates. The dashes signify the value of the minimal error and greater λ value. (C) Cox proportional hazards model integrated by 4 different types of immune cells. (D, G) Risk score distribution in training set (D) and validation set (G). (E, H) Survival overview in training set (E) and validation set (H). (F, I) Expression profile of four TICs in training set (F) and validation set (I). (J, K) Kaplan–Meier curves for OS by immunoscore group in the training set (J) and validation set (K). The differences between the two curves were determined by the two-side log-rank test. (L, M) Immunoscore measured by time‐dependent ROC curves in the training set (L) and validation set (M).

**
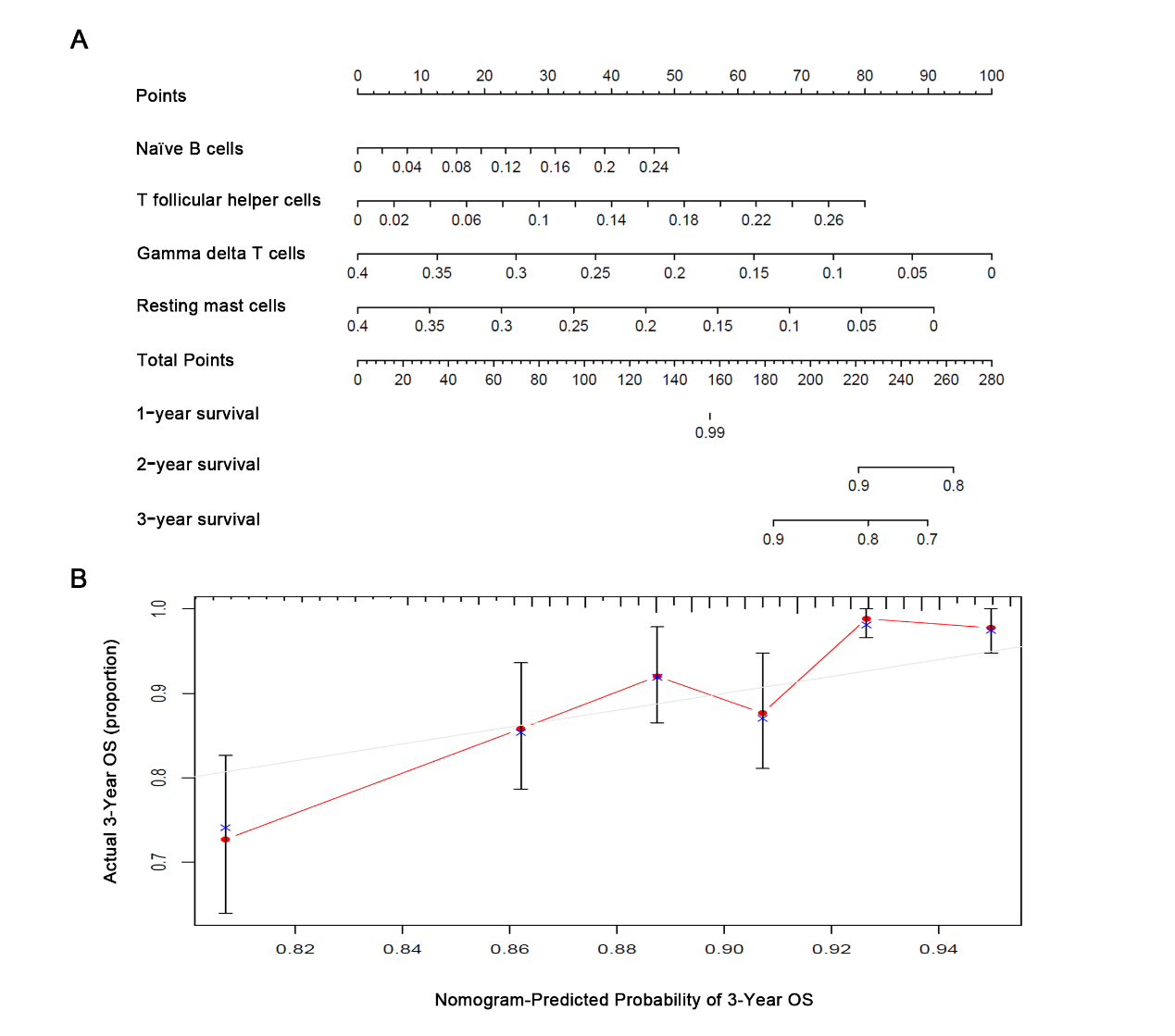
**

**Supplementary Figure 5.** Construction and validation of the nomogram model based on prognosis related immune cells. (A) Nomogram for predicting patients’ outcome based on four TICs in Panel. (B) The calibration curve of the nomogram to predict the probability of OS at 3years.


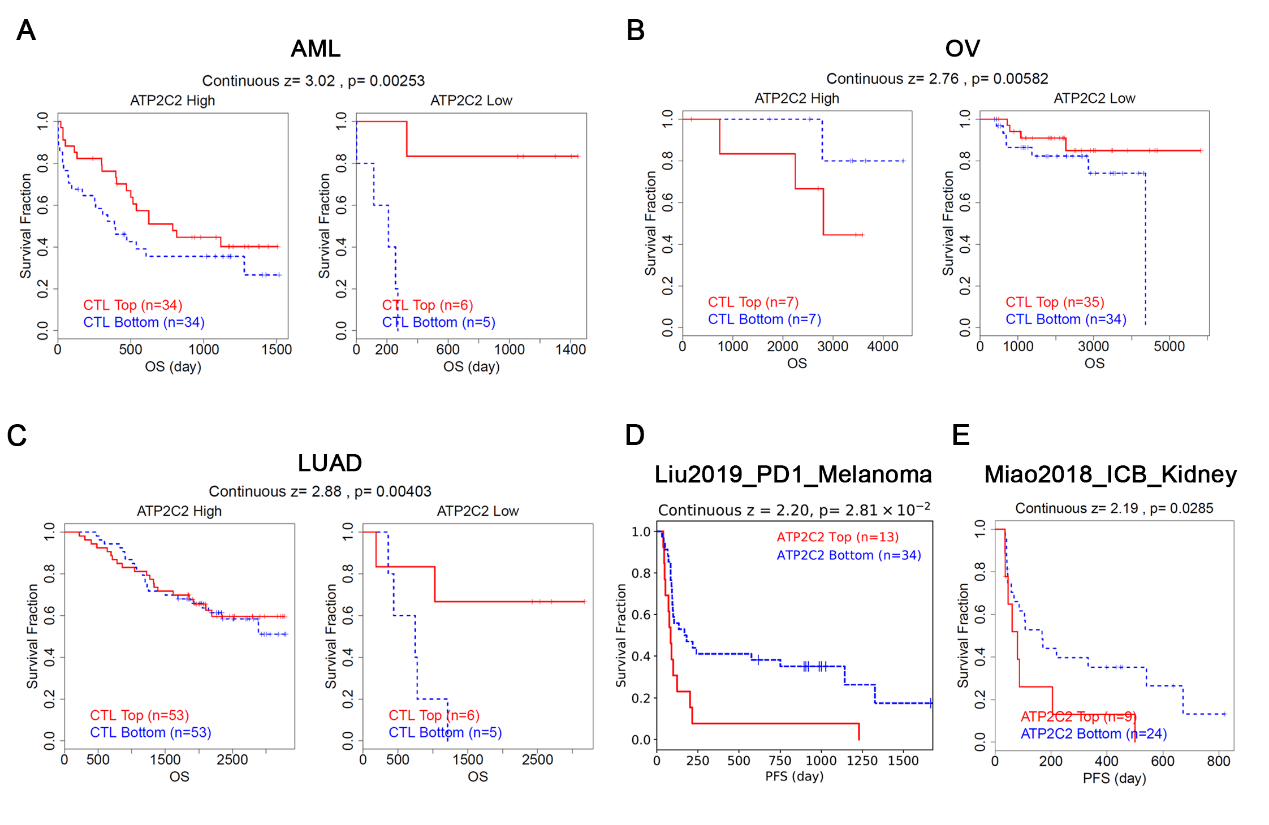


**Supplementary Figure 6.** Validation of ATP2C2 as a regulator of tumor immune escape. (A–C) The association between the CTL level and OS for AML, OV, and LUCD with different ATP2C2 levels. (D, E) Kaplan-Meier plots of patients with top half and bottom half ATP2C2 expression levels, using the data from ICIs studies.

## Supplementary Tables

**Supplementary Table 1.** Expression profiles and the forest plot of hazard ratio (HR) showing the prognostic values of the 67 DEGs associated with OS of BRCA.

| Variables | Univariate analysis | | | |
| --- | --- | --- | --- | --- |
|  | HR | HR.95L | HR.95H | *P*-value |
| ADD3 | 0.675352 | 0.588947 | 0.774433 | 1.91E-08 |
| ADRB2 | 0.694529 | 0.59355 | 0.812688 | 5.43E-06 |
| ALDH1A1 | 0.761825 | 0.680143 | 0.853317 | 2.59E-06 |
| AMPD1 | 0.667753 | 0.567648 | 0.785511 | 1.10E-06 |
| ANKRD36 | 1.243344 | 1.152146 | 1.341762 | 2.10E-08 |
| ATP2C2 | 1.355827 | 1.139192 | 1.61366 | 0.00061 |
| C5orf42 | 1.297322 | 1.138244 | 1.478633 | 9.62E-05 |
| CASP1 | 0.742945 | 0.645626 | 0.854935 | 3.36E-05 |
| CCL4 | 0.842725 | 0.771762 | 0.920214 | 0.000138 |
| CCR2 | 0.63567 | 0.531319 | 0.760515 | 7.34E-07 |
| CD2 | 0.809047 | 0.715607 | 0.914688 | 0.000714 |
| CD3D | 0.802226 | 0.715436 | 0.899544 | 0.000162 |
| CD48 | 0.756038 | 0.66543 | 0.858985 | 1.76E-05 |
| CECR1 | 0.814652 | 0.721871 | 0.919358 | 0.000891 |
| CELF2 | 0.635174 | 0.513906 | 0.785059 | 2.68E-05 |
| CFI | 0.706641 | 0.59965 | 0.832723 | 3.39E-05 |
| CLEC10A | 0.729402 | 0.610534 | 0.871413 | 0.000508 |
| CLEC2B | 0.745272 | 0.670243 | 0.8287 | 5.61E-08 |
| CRTAM | 0.745094 | 0.653977 | 0.848906 | 9.81E-06 |
| CXCL12 | 0.782237 | 0.687692 | 0.889781 | 0.000186 |
| EPN3 | 1.308122 | 1.14079 | 1.499998 | 0.00012 |
| EVI2A | 0.811138 | 0.726861 | 0.905187 | 0.000184 |
| FBXW12 | 0.865545 | 0.800883 | 0.935428 | 0.000267 |
| FMO1 | 0.797531 | 0.709821 | 0.89608 | 0.000141 |
| FXYD1 | 0.819269 | 0.729398 | 0.920213 | 0.000772 |
| GIMAP4 | 0.780832 | 0.67648 | 0.901281 | 0.000725 |
| GIMAP6 | 0.762792 | 0.665068 | 0.874875 | 0.000108 |
| GPR18 | 0.803458 | 0.713031 | 0.905353 | 0.000328 |
| GPR65 | 0.822345 | 0.732137 | 0.923668 | 0.000969 |
| GYPC | 0.63785 | 0.540679 | 0.752484 | 9.70E-08 |
| GZMA | 0.790278 | 0.705329 | 0.885459 | 4.98E-05 |
| GZMK | 0.829597 | 0.755702 | 0.910718 | 8.68E-05 |
| HCLS1 | 0.721927 | 0.623786 | 0.835509 | 1.24E-05 |
| HOXC8 | 1.270148 | 1.119694 | 1.440819 | 0.000201 |
| HSD11B1 | 0.732213 | 0.620936 | 0.863432 | 0.000211 |
| HTR2B | 0.8122 | 0.727017 | 0.907363 | 0.000234 |
| IL18R1 | 0.720233 | 0.605696 | 0.856429 | 0.000204 |
| IL2RB | 0.752944 | 0.637143 | 0.889791 | 0.000867 |
| IL7R | 0.791841 | 0.701678 | 0.893591 | 0.000154 |
| IRF9 | 0.720738 | 0.625748 | 0.830147 | 5.58E-06 |
| ITK | 0.782813 | 0.67927 | 0.90214 | 0.000718 |
| ITM2A | 0.758258 | 0.677109 | 0.849132 | 1.65E-06 |
| KLRB1 | 0.717439 | 0.637383 | 0.80755 | 3.78E-08 |
| KLRG1 | 0.723382 | 0.639879 | 0.817781 | 2.29E-07 |
| LGMN | 1.270249 | 1.103945 | 1.461607 | 0.000834 |
| LIPA | 0.68098 | 0.579268 | 0.800551 | 3.24E-06 |
| LXN | 0.792621 | 0.712497 | 0.881756 | 1.92E-05 |
| LY86 | 0.75935 | 0.682942 | 0.844306 | 3.62E-07 |
| LY96 | 0.80794 | 0.725002 | 0.900366 | 0.000114 |
| OLFML1 | 0.719367 | 0.630428 | 0.820854 | 9.99E-07 |
| PARD6B | 1.237131 | 1.122413 | 1.363574 | 1.82E-05 |
| PLAC8 | 0.767716 | 0.68472 | 0.860773 | 5.95E-06 |
| POU2F3 | 1.406345 | 1.216056 | 1.626409 | 4.28E-06 |
| RNASE6 | 0.771117 | 0.685711 | 0.867162 | 1.43E-05 |
| RNF2 | 1.373387 | 1.153795 | 1.634772 | 0.000358 |
| SELP | 0.630942 | 0.53003 | 0.751066 | 2.23E-07 |
| SERPINF1 | 0.763194 | 0.65702 | 0.886525 | 0.000406 |
| SLA | 0.745239 | 0.643871 | 0.862566 | 8.08E-05 |
| SLCO2B1 | 1.264435 | 1.105395 | 1.446357 | 0.000624 |
| STEAP1 | 0.772622 | 0.683644 | 0.873182 | 3.59E-05 |
| TM6SF1 | 0.689092 | 0.56427 | 0.841525 | 0.00026 |
| TMEM243 | 0.676692 | 0.573958 | 0.797815 | 3.34E-06 |
| TNFRSF17 | 0.86052 | 0.790558 | 0.936674 | 0.000516 |
| TREM2 | 0.809975 | 0.717914 | 0.913841 | 0.000618 |
| TRIM22 | 0.707233 | 0.621562 | 0.804711 | 1.46E-07 |
| UCN | 1.266006 | 1.127005 | 1.422152 | 7.04E-05 |
| ZNF813 | 1.302961 | 1.185483 | 1.432081 | 4.03E-08 |

*HR* hazard ratio
